# Mitochondrial HSF1 triggers mitochondrial dysfunction and neurodegeneration in Huntington's disease

**DOI:** 10.15252/emmm.202215851

**Published:** 2022-06-07

**Authors:** Chunyue Liu, Zixing Fu, Shanshan Wu, Xiaosong Wang, Shengrong Zhang, Chu Chu, Yuan Hong, Wenbo Wu, Shengqi Chen, Yueqing Jiang, Yang Wu, Yongbo Song, Yan Liu, Xing Guo

**Affiliations:** ^1^ State Key Laboratory of Reproductive Medicine Key Laboratory of Human Functional Genomics of Jiangsu Province Department of Neurobiology Interdisciplinary InnoCenter for Organoids School of Basic Medical Sciences Nanjing Medical University Nanjing China; ^2^ State Key Laboratory of Reproductive Medicine Interdisciplinary InnoCenter for Organoids Institute for Stem Cell and Neural Regeneration School of Pharmacy Nanjing Medical University Nanjing China; ^3^ State Key Laboratory of Magnetic Resonance and Atomic and Molecular Physics Key Laboratory of Magnetic Resonance in Biological Systems Wuhan Center for Magnetic Resonance, Innovation Academy for Precision Measurement Science and Technology Chinese Academy of Sciences Wuhan China; ^4^ Department of Pharmacology Shenyang Pharmaceutical University Shenyang China; ^5^ Department of Endocrinology Sir Run Run Hospital Nanjing Medical University Nanjing Jiangsu China

**Keywords:** heat shock transcription factor 1, human striatal organoids, Huntington's disease, mitochondrial DNA, single‐stranded DNA‐binding protein 1, Metabolism, Neuroscience

## Abstract

Aberrant localization of proteins to mitochondria disturbs mitochondrial function and contributes to the pathogenesis of Huntington’s disease (HD). However, the crucial factors and the molecular mechanisms remain elusive. Here, we found that heat shock transcription factor 1 (HSF1) accumulates in the mitochondria of HD cell models, a YAC128 mouse model, and human striatal organoids derived from HD induced pluripotent stem cells (iPSCs). Overexpression of mitochondria‐targeting HSF1 (mtHSF1) in the striatum causes neurodegeneration and HD‐like behavior in mice. Mechanistically, mtHSF1 facilitates mitochondrial fission by activating dynamin‐related protein 1 (Drp1) phosphorylation at S616. Moreover, mtHSF1 suppresses single‐stranded DNA‐binding protein 1 (SSBP1) oligomer formation, which results in mitochondrial DNA (mtDNA) deletion. The suppression of HSF1 mitochondrial localization by DH1, a unique peptide inhibitor, abolishes HSF1‐induced mitochondrial abnormalities and ameliorates deficits in an HD animal model and human striatal organoids. Altogether, our findings describe an unsuspected role of HSF1 in contributing to mitochondrial dysfunction, which may provide a promising therapeutic target for HD.

## Graphical abstract

Inhibition of mitochondrial HSF1(mtHSF1) restores mitochondrial function and slows neurodegeneration in Huntington's Disease (HD) mice, suggesting that mtHSF1 might be a therapeutic target for treating HD.

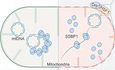

**The paper explained**

**Problem**

Huntington’s disease (HD) is a neurodegenerative disease caused by genetic mutation of huntingtin (htt), which disrupts protein distribution and causes striatal medium spiny neural degeneration. So far, there is no effective treatment of HD. We previously found the deficits in mitochondrial quality control caused by mislocalized proteins are closely associated with HD. Heat shock transcription factor (HSF) 1 is a predominantly cytosolic transcription factor, which contributes to the pathogenesis of HD. However, it remains elusive whether HSF1 directly regulates mitochondrial function independent of its role in regulating nuclear gene expression. Moreover, whether HSF1 could be a potential therapeutic target for the treatment of HD is also unclear.

**Results**

We first found HSF1 accumulates in the mitochondria of various HD models. Overexpression of mitochondria‐targeting HSF1 (mtHSF1) in the striatum causes neurodegeneration and HD‐like behavior. Mechanistically, mtHSF1 facilitates mitochondrial fission by activating dynamin‐related protein 1 (Drp1) phosphorylation at S616. Moreover, mtHSF1 suppresses single‐stranded DNA‐binding protein 1 (SSBP1) oligomer formation, which results in mitochondrial DNA (mtDNA) deletion. Furthermore, to block Drp1/HSF1 binding that may reduce HSF1 accumulation in mitochondria, we next design and synthesize a unique peptide inhibitor, DH1, according to the region of homology between Drp1 and HSF1. DH1 remarkably abolishes HSF1‐induced mitochondrial abnormalities and ameliorates deficits in an HD animal model and human striatal organoids.

**Impact**

We remarkably show that HSF1 accumulates on mitochondria and causes neurodegeneration and movement deficits. Notably, treatment with DH1 improved neurotoxicity and animal behavior, indicating that blocking HSF1 translocation to mitochondria may slow the pathogenesis of HD. Indeed, our results reveal an unsuspected role of HSF1 in mitochondria, which may provide a therapeutic target for HD.

## Introduction

Huntington's disease (HD) is a neurodegenerative disease caused by genetic mutation of huntingtin (htt) (Tabrizi *et al*, 2020). Mutant huntingtin (mtHtt) disrupts protein distribution, resulting in the dysfunction of physiological processes and subsequent neurodegeneration. Valosin‐containing protein (VCP) recruitment to mitochondria triggers excessive mitophagy and neurodegeneration in HD (Guo *et al*, 2016). The tumor suppressor P53 shuttles from the nucleus to the mitochondria and activates mitochondrial fission (Guo *et al*, 2014). These findings indicate that defects in mitochondrial quality control caused by mislocalized proteins are closely associated with HD. Therefore, identifying proteins that are mislocalized to mitochondria and revealing the underlying mechanisms may be useful for HD treatment.

Heat shock transcription factor (HSF) 1 is a predominantly cytosolic transcription factor that is inhibited by Hsp40, Hsp70, Hsp90, and TRiC under physiological conditions (Neef *et al*, 2014; Gomez‐Pastor *et al*, 2018). Upon activation, phosphorylated HSF1 assembles into a homotrimer and accumulates in the nucleus to regulate gene expression (Morimoto, 1998; Akerfelt *et al*, 2010). Previous studies have revealed that HSF1 deficiency contributes to the pathogenesis of HD. The transcriptional activity of HSF1, a master regulator of heat shock protein (HSP), is suppressed by mtHtt in R6/2 mice (Labbadia *et al*, 2011). Hyperactivation of the E3 ligase Fbxw7 promotes HSF1 polyubiquitination and proteasome‐mediated degradation (Kourtis *et al*, 2015). In addition, recent studies have demonstrated that deletion of HSF1 elicits mitochondrial dysfunction by inducing oxidative stress, attenuating respiration activity, and inhibiting mitochondrial biogenesis (Homma *et al*, 2007; Ma *et al*, 2015; Qiao *et al*, 2017; Intihar *et al*, 2019). However, it remains elusive whether HSF1 directly regulates mitochondrial function independent of its role in regulating nuclear gene expression.

Here, we show that HSF1 accumulates on mitochondria and causes neurodegeneration and movement deficits. We observed that mitochondria‐targeting HSF1 (mtHSF1) shortens mitochondria by evoking dynamin‐related protein 1 (Drp1) phosphorylation at S616. In addition, expression of mtHSF1 results in decreased single‐stranded DNA‐binding protein 1 (SSBP1) oligomerization and subsequent mitochondrial DNA (mtDNA) deletion. Furthermore, blockade of HSF1 mitochondrial localization by DH1 reduces mitochondrial dysfunction and neurotoxicity in HD.

## Results

### HSF1 associates with mitochondria in models of HD

**Figure 1:**
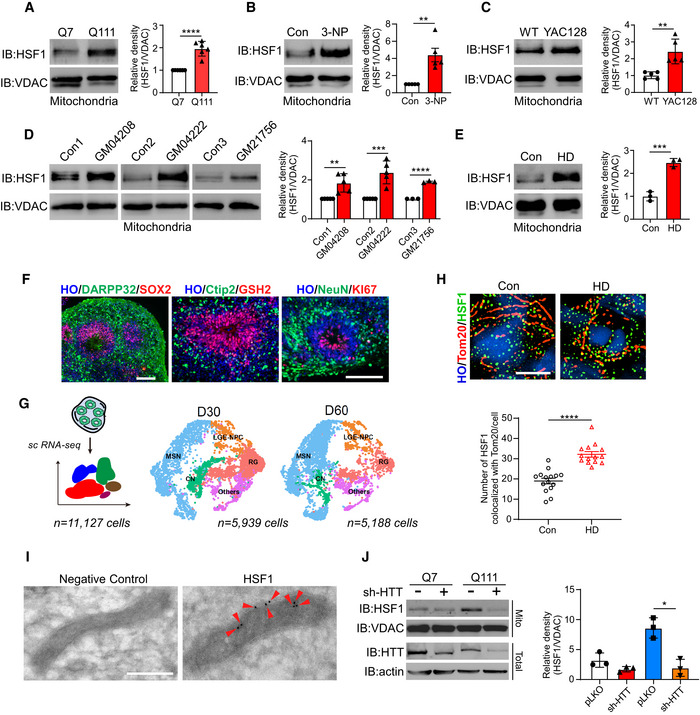
HSF1 associates with mitochondria in models of HD Mitochondria‐enriched fractions were isolated from HdhQ7 or HdhQ111 cells. mtHSF1 was examined by WB analysis. VDAC was analyzed as a loading control (*n* = 6 biological replicates).HdhQ7 WT striatal cells were exposed to 5 mM 3‐NP for 2 h. mtHSF1 levels were determined by immunoblotting. VDAC was used as a loading control (*n* = 5 biological replicates).HSF1 was measured in the mitochondria‐enriched fractions of the HD transgenic YAC128 mice (9 months old) or age‐matched littermates (*n* = 5 biological replicates).mtHSF1 was analyzed in HD patient fibroblasts (GM04208, GM04222, and GM21756) and fibroblasts from healthy individuals (*n* = 5 for Con1/ GM04208 and Con2/ GM04222 groups; *n* = 3 for Con3/ GM21756 group).Representative immunoblot of HSF1 levels in mitochondria isolated from patient‐derived iPSCs and control iPSCs (*n* = 3 biological replicates).Representative images of striatal organoids. Left, immunostaining for the MSN marker DARPP32 (green) and the radial glial marker SOX2 (red) in striatal organoids at D60. Middle, immunostaining for the LGE progenitor markers Ctip2 (green) and GSH2 (red) at D30. Right panel, immunostaining for the neuronal marker NeuN (green) and the proliferation marker Ki67 (red) at D30. The images are representative of 12 independent differentiation experiments of H9 and IMR90‐4 cells. HO, Hoechst (blue). The scale bar represents 100 µm.Left, schematic of the procedure by which the striatal organoids were dissociated for scRNA‐seq. Right, UMAP visualization of single‐cell RNA expression in striatal organoids at D30 (*n* = 5,939 cells) and D60 (*n* = 5,188 cells).Representative images and scatterplot confirming the increased HSF1 (green) translocation to mitochondria (Tom20, red). The data were obtained from 3 independent biological experiments. Con: *n* = 15 organoids; HD: *n* = 14 organoids. Two‐tailed unpaired *t*‐test; means ± SEMs. Images were taken using structured illumination microscopy (SIM). The scale bar represents 5 μm.The subcellular localization of HSF1 in HD striatal organoids was observed by immunoelectron microscopy. The arrows (red) mark HSF1 in mitochondria. The negative control lacked the HSF1 antibody. The scale bar represents 200 nm.HTT was knocked down in HdhQ7 or HdhQ111 cells by lentiviral infection. The protein levels of HSF1 were detected in mitochondrial fractions (*n* = 3 biological replicates). Data information: The data are the means ± SEMs from at least three independent biological experiments; unpaired Student’s *t*‐test was used in (A–E) and (H); one‐way ANOVA followed by Tukey’s multiple comparison test was used in (J). *P < 0.05, **P < 0.01, ***P < 0.001, and ****P < 0.0001. Source data are available online for this figure.

**Figure EV1:**
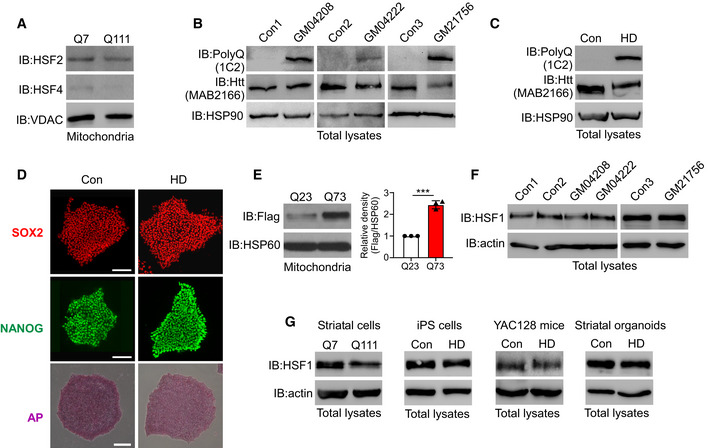
HSF1 associates with mitochondria AMitochondria‐enriched fractions were isolated from HdhQ7 or HdhQ111 cells. HSF2 and HSF4 protein levels in the mitochondria were detected by WB analysis (*n* = 3 biological replicates).B, CTotal lysates were harvested from control iPSCs, HD iPSCs, control fibroblasts, and HD patient fibroblasts. Immunoblot analysis of htt was performed with 1C2 and MAB2166 (*n* = 3 biological replicates).DCharacterization of hiPSCs by immunostaining for the pluripotency markers SOX2 and NANOG and staining for alkaline phosphatase (AP) (*n* = 2 biological replicates). The scale bar represents 100 μm.EFlag‐HSF1 was transfected with Myc‐Q23 or Myc‐Q73 into HEK293 cells. Mitochondria‐localized HSF1 was examined by WB analysis (*n* = 3 biological replicates). The data are the means ± SEMs; unpaired Student’s *t*‐test was used. ***P < 0.001.FTotal HSF1 levels in fibroblasts were measured by WB analysis (*n* = 2 biological replicates).GTotal HSF1 protein levels were tested in the indicated samples by immunoblotting (*n* = 3 biological replicates). Source data are available online for this figure.

**Figure EV2:**
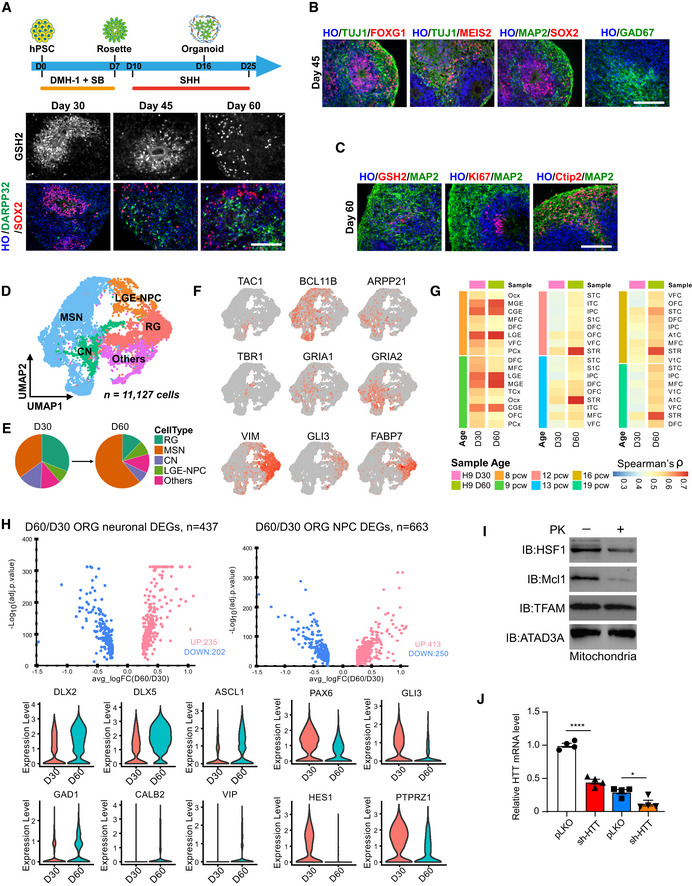
Construction of mtHSF1 and the procedure for human striatal organoid differentiation Schematic of the protocol for differentiating human striatum‐like organoids from hPSCs and immunostaining for the LGE progenitor marker GSH2 and the MSN marker DARPP32 at D30, D45, and D60. The scale bar represents 100 μm.Identification of telencephalon and striatum markers by immunostaining. The scale bar represents 100 μm.Confirmation of maturation of striatal organoids by robust MAP2 staining and sparse GSH2 and KI67 staining. The scale bar represents 100 μm.UMAP visualization of single‐cell RNA expression in striatal organoids (*n* = 11,127 cells).Cell‐type compositions of human striatal organoids at D30 and D60.UMAP plots showing the gene expression patterns of representative marker genes for each cell type. The relative expression level is indicated by the color from gray to red.Correlation with BrainSpan dataset of the developing human brain (PCW 8–19).Volcano and violin plots for cell type‐specific genes differentially expressed in neuronal (left) cells and neural progenitor cells (right) from striatal organoids at D30 and D60.Mitochondria‐enriched fractions isolated from HdhQ111 cells were exposed to proteinase K for 20 min at 3 µg/ml. Protein levels were detected with the indicated antibodies.The htt gene expression was measured by real‐time PCR (*n* = 3 biological replicates). Data information: The data are the means ± SEMs; unpaired Student’s *t*‐test was used. *P < 0.05 and ****P < 0.0001. Source data are available online for this figure.

We first isolated mitochondria‐enriched fractions from HdhQ7 or HdhQ111 cells, which are commonly used model cell lines for HD research. Western blot (WB) analysis showed that endogenous HSF1 levels in mitochondria were dramatically higher in HdhQ111 cells than in HdhQ7 cells (Fig 1A). However, other members of the HSF family, including HSF2 and HSF4, were not affected by the presence of mtHtt (Fig EV1A). Wild‐type (WT) striatal cells exposed to 3‐nitropropionic acid (3‐NP), an inducer of HD‐like symptoms, strongly promoted HSF1 association with mitochondria (Fig 1B). Consistently, we observed higher HSF1 levels in mitochondria in YAC128 HD transgenic mice than in their age‐matched littermates (Fig 1C). Moreover, elevated binding of HSF1 with mitochondria was confirmed in three lines of HD patient fibroblasts (GM04208, GM04222, and GM21756) and fibroblasts from healthy individuals (Figs 1D and EV1B). Induced pluripotent stem cells (iPSCs) derived from a HD patient carrying 75 CAG repeats were confirmed by WB and pluripotency staining (Fig EV1C and D). There were significantly higher HSF1 levels in mitochondria in HD iPSCs (HDUE003) than in control iPSCs (IMR90‐4) (Fig 1E). Overexpression of exon 1 of htt with 73 polyglutamine repeats sharply increased HSF1 translocation to mitochondria (Fig EV1E). Notably, the total protein levels of HSF1 were not different between the HD models and their controls (Fig EV1F and G). To further confirm the results in human tissues, we established a method to generate human striatal organoids for research on the etiology of HD (Fig EV2A). The striatal organoids expressed markers for medium spiny neurons (MSNs, DARPP32+) and lateral ganglionic eminences (LGEs, GSH2+), as well as telencephalic markers (Figs 1F and, EV2A–C), indicating a striatal fate. We evaluated the characteristics of the striatal organoids by performing 10x Genomics single‐cell RNA sequencing (scRNA‐seq) on day 30 and day 60 (Fig 1G). Uniform Manifold Approximation and Projection (UMAP) visualization showed that the number of cells in the subcluster of MSN and LGE progenitors was significantly increased in day (D) 60 organoids (Figs 1G and EV2D–F). Moreover, comparison of our scRNA‐seq data with the BrainSpan database showed a positive correlation between D60 striatal organoids and the fetal striatum at 12 to 19 weeks post‐conception (Fig EV2G). Furthermore, differentially expressed gene (DEG) analysis revealed upregulation of striatal genes but downregulation of neural progenitor genes in the D60 striatal organoids (Fig EV2H). Overall, we generated human striatal organoids confirmed by immunohistochemistry and scRNA‐seq. HD striatal organoids exhibited increased colocalization of HSF1 and mitochondria compared with WT striatal organoids (Fig 1H). Treatment with proteinase K resulted in the digestion of the outer mitochondrial membrane (OMM) protein Mcl1 and approximately 60% of mtHSF1, suggesting that part of HSF1 localizes to the OMM (Fig EV2I). Immunogold electron microscopy further determined that HSF1 localizes to the OMM, inner membrane space, and matrix (Fig 1I). Deletion of mtHtt in HdhQ111 cells with shRNA against Htt reduced the association between HSF1 and mitochondria (Figs 1J and EV2J). Taken together, these data suggest that HSF1 accumulates on mitochondria in the models of HD.

### mtHSF1 disturbs dynamic balance by promoting Drp1 phosphorylation

**Figure 2:**
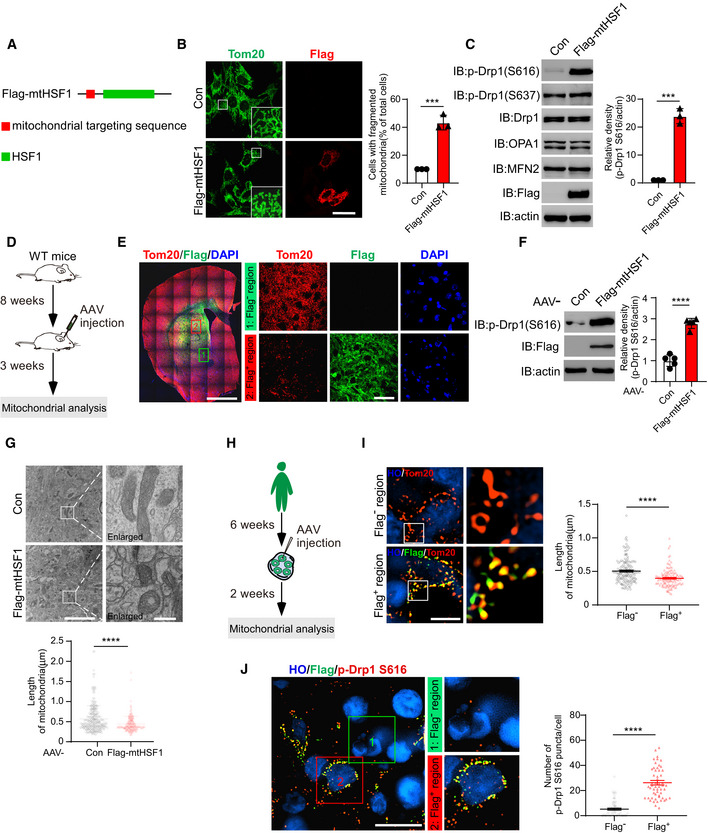
mtHSF1 disturbs the dynamic balance by promoting Drp1 phosphorylation Sketch of mitochondrial targeting by HSF1.HdhQ7 cells were transfected with Flag‐mtHSF1 and stained with anti‐Tom20 (green) and anti‐Flag (red) antibodies. Mitochondrial morphology was examined by confocal microscopy. Scatterplot with bar shows the percentage of cells with mitochondrial fragmentation. The scale bar represents 40 µm (*n* = 3 biological replicates; at least 100 cells per group were counted).The total protein levels of p‐Drp1 S616, p‐Drp1S637, Drp1, OPA1, MFN2, Flag‐mtHSF1, and actin (loading control) were determined by WB analysis (*n* = 3 biological replicates).Timeline overview of mitochondrial analysis in mice. AAV‐Con and AAV‐mtHSF1 were injected into the striata of WT mice at 8 weeks of age. Mitochondrial morphology was analyzed after 3 weeks.Brain sections were stained with an anti‐Tom20 antibody (red), an anti‐Flag antibody (green), and DAPI (blue). Mitochondrial morphology was analyzed in Flag^+^ or Flag^‐^ regions by confocal microscopy. The scale bar represents 2 mm, and the enlarged image scale bar is 40 µm (*n* = 6 mice/group).Striata injected with AAVs were lysed. p‐Drp1 S616 was tested by immunoblotting (*n* = 5 mice/group).Mitochondrial morphology was analyzed by electron microscopy (EM) in mice injected with AAV‐Con or AAV‐mtHSF1. Scatterplot shows the length of mitochondria. The scale bar represents 4 µm, and the enlarged image scale bar is 400 nm (*n* = 3 mice/group). AAV‐Con group, *n* = 268 mitochondria were measured; AAV‐Flag‐mtHSF1 group, *n* = 179 mitochondria were measured.Timeline overview of mitochondrial analysis in striatal organoids. AAV‐mtHSF1 was injected into the WT striatal organoids at 6 weeks. Mitochondrial analysis was performed after 2 weeks.Representative images and scatterplot of mitochondrial length (Tom20, red) in Flag^+^ or Flag^‐^ cells. The data were obtained from 3 independent biological experiments. Flag^‐^: *n* = 154 mitochondria; Flag^+^: *n* = 132 mitochondria. The scale bar represents 5μm.Representative images and scatterplot showing that the number of p‐Drp1 S616 puncta in Flag^+^ cells was greater than that in Flag^‐^ cells. The data were obtained from 3 independent biological experiments. Flag^‐^: *n* = 70 cells from 10 organoids; Flag^+^: *n* = 55 cells from 10 organoids. The images were obtained using structured illumination microscopy (SIM). The scale bar represents 10 µm. Data information: The data are the means ± SEMs from at least three independent biological experiments; unpaired Student’s *t*‐test was used. ***P < 0.001 and ****P < 0.0001. Source data are available online for this figure.

**Figure EV3:**
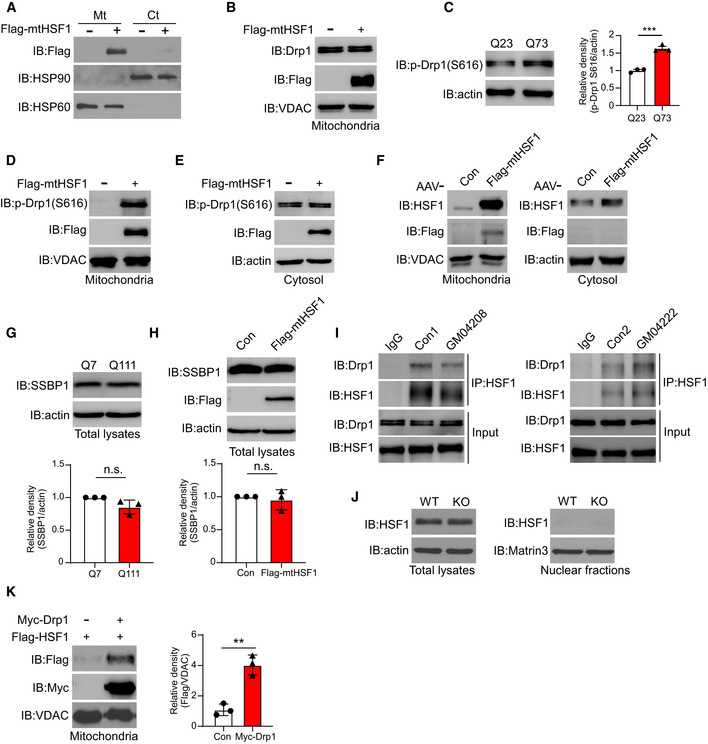
HSF1‐induced mitochondrial dysfunction is mediated by Drp1 and SSBP1 AAn empty vector or Flag‐mtHSF1 was transfected into HEK293 cells. Isolated mitochondria‐enriched fractions were analyzed by WB analysis (*n* = 2 biological replicates).BFlag‐mtHSF1 was transfected into HdhQ7 cells. Drp1 protein levels were determined in the mitochondria‐enriched fractions by immunoblotting (*n* = 3 biological replicates).CMyc‐Q23 or Myc‐Q73 was transfected into HdhQ7 cells. The p‐Drp1 S616 levels was examined by WB analysis (*n* = 3 biological replicates). The data are the means ± SEMs; unpaired Student’s *t*‐test was used. ***P < 0.001.D, EThe protein levels of p‐Drp1(S616) were determined in mitochondria‐enriched fractions or cytosolic fractions by WB analysis (*n* = 3 biological replicates).FAAV‐Con or AAV‐mtHSF1 was injected into WT mice. The sublocalization of mtHSF1 was determined with anti‐HSF1 or anti‐Flag antibodies (*n* = 2 biological replicates).GSSBP1 protein levels in HdhQ7 or HdhQ111 cells were determined by WB analysis (*n* = 3 biological replicates).HFlag‐mtHSF1 was transfected into HdhQ7 cells. The total levels of SSBP1 were tested by immunoblotting (*n* = 3 biological replicates).IPatient fibroblasts and control fibroblasts were harvested and subjected to immunoprecipitation with an anti‐HSF1 antibody. The immunoprecipitates were analyzed by WB analysis (*n* = 2 biological replicates).JLeft: total protein from WT or Drp1‐KO MEFs was immunoblotted with an anti‐HSF1 antibody. Right: nuclear fractions of WT or Drp1‐KO MEFs were collected and analyzed by immunoblotting (*n* = 2 biological replicates).KFlag‐HSF1 was transfected with Myc‐Drp1 or an empty vector into HEK293 cells. HSF1 was analyzed in the mitochondria‐enriched fractions by immunoblotting (*n* = 3 biological replicates). Data information: The data are the means ± SEMs; unpaired Student’s *t*‐test was used. **P < 0.01. Source data are available online for this figure.

To determine the effect of mtHSF1 on mitochondrial morphology, we constructed Flag‐tagged mtHSF1 (Flag‐mtHSF1) by fusing a mitochondrial targeting sequence (MTS) to the N‐terminus of HSF1 to mimic the accumulation of HSF1 on mitochondria in HD (Figs 2A and EV3A). We observed shortened and swollen mitochondria in striatal cells expressing Flag‐mtHSF1, suggesting that the mitochondria were fragmented (Fig 2B). To understand the molecular basis of the observed mitochondrial fragmentation, we examined the protein levels of mitochondrial dynamics‐related proteins. The presence of Flag‐mtHSF1 had no effect on the total protein levels of Drp1, optic atrophy 1 (OPA1), mitofusin 2 (MFN2), or mitochondria‐localized Drp1 (Figs 2C and EV3B).

Overexpression of Httex1‐Q73 promotes the phosphorylation of Drp1 at the S616 residue (p‐Drp1 S616), which is a key step for Drp1 activation and mitochondrial fission (Fig EV3C). Strikingly, the p‐Drp1 S616 levels were elevated in mitochondria in Flag‐mtHSF1‐expressing cells (Figs 2C and EV3D and E). To verify that mtHSF1 affects mitochondrial morphology *in vivo*, we delivered Flag‐mtHSF1 to the striatum in WT mice with an adeno‐associated virus (AAV)‐mediated gene expression system for three weeks (Figs 2D and EV3F). The mitochondria in the Flag^‐^ area exhibited a tubular morphology, whereas the expression of mtHSF1 impaired the mitochondrial network (Fig 2E). In addition, mice injected with the mtHSF1‐expressing AAV (AAV‐mtHSF1) showed shorter mitochondria and higher p‐Drp1 S616 levels than mice injected with the control AAV (AAV‐Con) (Fig 2F and G). Next, we injected AAV‐mtHSF1 into the striatal organoids (Fig 2H) and found that the mitochondria in Flag^+^ cells were shorter than those in Flag^‐^ cells (Fig 2I). Immunostaining analysis showed that expression of mtHSF1 dramatically increased p‐Drp1 S616 levels (Fig 2J), consistent with the results found in cell cultures and mice. Thus, mtHSF1 induces mitochondrial fragmentation by activating Drp1 phosphorylation *in vitro* and *in vivo*.

### mtHSF1 triggers mtDNA deletion and cell death

**Figure 3:**
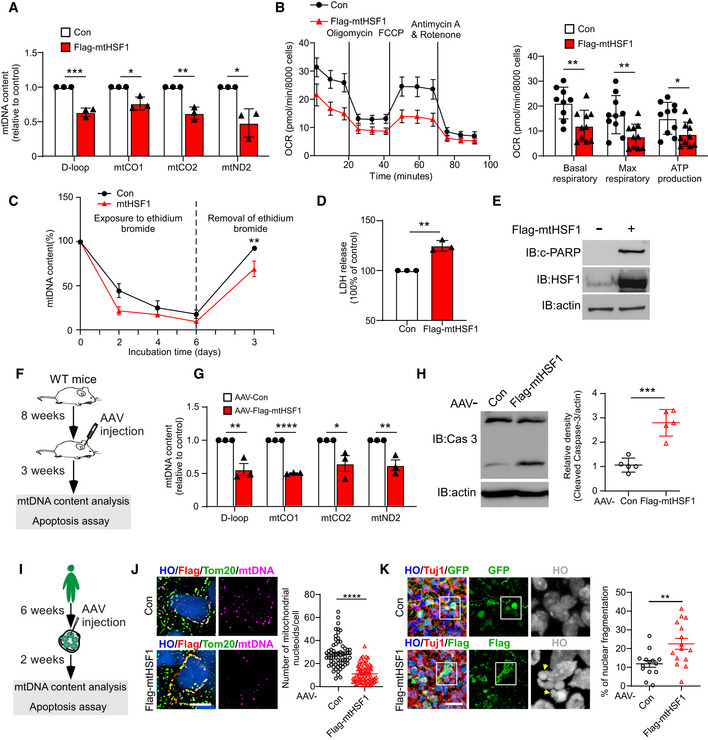
mtHSF1 triggers mtDNA deletion and cell death AThe mtDNA content was examined with primers of D‐loop, mtCO1, mtCO2 and mtND2 by qPCR in control or Flag‐mtHSF1‐expressing striatal cells. Scatterplot with bar shows the relative percentage of mtDNA content (*n* = 3 biological replicates).BMeasurement of mitochondrial respiratory activity by Seahorse analysis (*n* = 9–10 biological replicates)CCells were treated with EtBr for six days to delete mtDNA. mtDNA repopulation efficiency was measured by qPCR after withdrawal of EtBr (*n* = 3 biological replicates).D, ECell death was determined by detection of LDH release into the medium and cleavage of PARP. Scatterplot with bar shows the relative percentage of LDH release (*n* = 3 biological replicates).FTimeline overview of mtDNA and cell death analysis in mice.GqPCR analysis of the mtDNA content of AAV‐Con‐injected mice or AAV‐mtHSF1‐injected mice. Scatterplot with bar shows the relative percentage of mtDNA content (*n* = 3 mice/group).HCleaved‐caspase‐3 in AAV‐Con‐ or AAV‐mtHSF1‐injected mice was detected by WB analysis (*n* = 5 mice/group).ITimeline overview of mtDNA and cell death analysis in striatal organoids.JRepresentative images and scatterplot showing the number of mtDNA (purple) colocalized with mitochondria (Tom20, green) in striatal organoids injected with AAV‐Con or AAV‐mtHSF1. The data were obtained from 3 independent biological experiments. Con: *n* = 65 cells from 20 organoids; Flag‐mtHSF1: *n* = 70 cells from 20 organoids. Two‐tailed unpaired *t*‐test was used. Images were captured by structured illumination microscopy (SIM). The scale bar represents 5 μm.KThe typical nuclear fragmentation morphology of AAV‐Con‐ (GFP) or AAV‐mtHSF1‐injected striatal organoids was measured. Scatterplot shows the quantitative result of nuclear fragmentation. The arrows (yellow) mark fragmented nuclei. Two‐tailed unpaired *t*‐test was used. The data were obtained from 3 independent biological experiments. Con: *n* = 14 organoids; Flag‐mtHSF1: *n* = 15 organoids. The scale bar represents 20 µm. Data information: The data are the means ± SEMs from at least three independent biological experiments; unpaired Student’s *t*‐test was used. *P < 0.05, **P < 0.01, ***P < 0.001, and ****P < 0.0001. Source data are available online for this figure.

Abnormalities in mitochondrial dynamics have been linked to defects in oxidative phosphorylation and energy metabolism (MacVicar & Lane, 2014). mtDNA‐encoded proteins compose complexes implicated in the electron transport system and ATP production (Falkenberg *et al*, 2007). We first evaluated the mtDNA content and found that expression of Flag‐tagged mtHSF1 greatly reduced the mtDNA content in WT striatal cells (Fig 3A). We next measured mitochondrial respiration and found that the presence of mtHSF1 significantly reduced the mitochondrial maximal respiratory rate, basal respiratory rate, and ATP content (Fig 3B). Treatment with ethidium bromide (EtBr) for six days caused mtDNA deletion (Fig 3C). After withdrawing EtBr, we found that stably overexpressing mtHSF1 repressed mtDNA repopulation efficiency (Fig 3C). Moreover, overexpression of Flag‐mtHSF1 enhanced the release of lactate dehydrogenase (LDH) and cleavage of PARP, which are markers of cell death (Fig 3D and E). Similarly, decreased mtDNA copy numbers were observed in mtHSF1‐expressing mice and human striatal organoids (Fig 3F and G, and I, and J). An elevated cell death rate was confirmed via assessment of caspase‐3 cleavage and nuclear fragmentation, respectively (Fig 3H and K). Taken together, these results demonstrate that mtHSF1 triggers mtDNA deletion and subsequent neuronal cell death.

### mtHSF1 suppresses SSBP1 oligomerization

**Figure 4:**
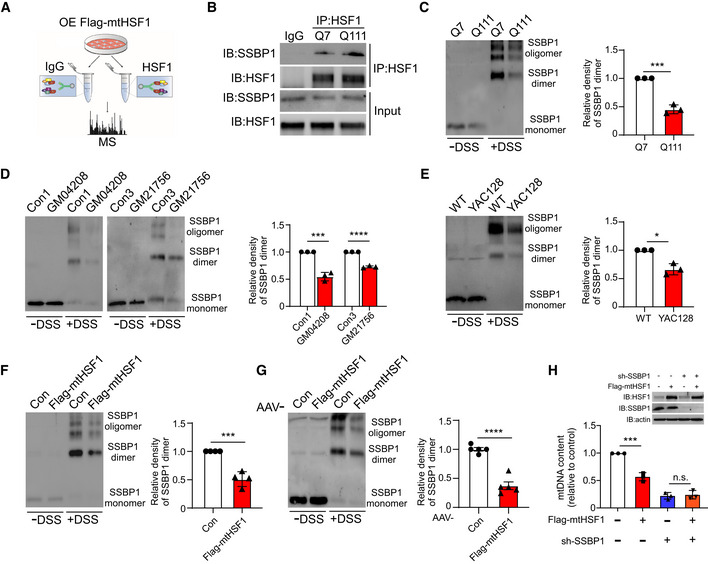
mtHSF1 suppresses SSBP1 oligomerization Mitochondria‐enriched fractions were subjected to immunoprecipitation with IgG or an anti‐HSF1 antibody. The immunoprecipitates were then analyzed by mass spectrometry.Total cell lysates were subjected to immunoprecipitation with an anti‐HSF1 antibody followed by WB analysis (*n* = 3 biological replicates).HdhQ7 or HdhQ111 cell extracts were incubated with DSS (1 mM) for 30 min at RT. SSBP1 oligomers were detected by immunoblotting with an anti‐SSBP1 antibody (*n* = 3 biological replicates).SSBP1 oligomers in control and patient fibroblasts (GM04208 and GM21756) were analyzed (*n* = 3 biological replicates).SSBP1 oligomers were measured in WT mice or YAC128 mice (*n* = 3 mice/group).Cells were transfected with an empty vector or Flag‐mtHSF1 for 48 h. The total cell lysates were harvested and cross‐linked with DSS. SSBP1 oligomers were tested by immunoblotting (*n* = 4 biological replicates).Flag‐mtHSF1 was expressed in the striata of WT mice for three weeks. SSBP1 oligomers were determined by immunoblotting (*n* = 5 mice/group).Flag‐mtHSF1 or sh‐SSBP1 was delivered into cells via lentiviral infection. The expression of SSBP1 and mtHSF1 was detected by WB analysis. The mtDNA content was examined by qPCR analysis (*n* = 3 biological replicates). Data information: The data are the means ± SEMs from at least three independent biological experiments; unpaired Student’s *t*‐test was used in (C–G), and one‐way ANOVA followed by Tukey’s multiple comparison test was used in (H). *P < 0.05, ***P < 0.001, and ****P < 0.0001, n.s., not significant. Source data are available online for this figure.

In order to better define the underlying molecular mechanism of mtHSF1‐induced mtDNA deletion, we performed mass spectrometry‐based analysis to identify the proteins that form a complex with mtHSF1 on mitochondria (Fig 4A). A total of 17 unique proteins were identified (Appendix Table S1). Among these proteins, SSBP1, a nuclear‐encoded mitochondrial protein, is essential for maintaining mtDNA stability and replication (Korhonen *et al*, 2004; Ruhanen *et al*, 2010; Miralles Fuste *et al*, 2014; Kaur *et al*, 2018). We validated the interaction between HSF1 and SSBP1 and found elevated binding affinity in HdhQ111 striatal cells (Fig 4B). However, there was little difference in total SSBP1 protein content between HD striatal cells or mtHSF1‐expressing cells and the corresponding control cells (Fig EV3G and H). SSBP1 assembles into multimeric complexes, and dimers/tetramers of SSBP1 are critical for mtDNA replication (Del Dotto *et al*, 2020; Piro‐Megy *et al*, 2020). We thus hypothesized that mtHSF1 may affect the oligomerization of SSBP1. To test this possibility, we first examined SSBP1 oligomers in HD cell cultures or HD animal models. The levels of SSBP1 dimers/tetramers were markedly decreased in HD striatal cells, HD patient fibroblasts, and HD YAC128 mice compared with related controls (Fig 4C–E). Furthermore, the overexpression of Flag‐mtHSF1 in WT striatal cells reduced the oligomerization of SSBP1 (Fig 4F). Consistent with the results in striatal cells, the dimer/tetramer levels of SSBP1 were dramatically decreased in the striatal tissues of WT mice injected with AAV‐mtHSF1 relative to those of mice injected with AAV‐Con (Fig 4G). Downregulation of SSBP1 with shRNA abolished the mtHSF1‐induced reduction in mtDNA content (Fig 4H), suggesting that SSBP1 is required for mtHSF1‐triggered mtDNA deletion. Thus, we conclude that mtHSF1 binds to SSBP1 on mitochondria and disrupts its oligomer formation, which in turn results in suppression of mtDNA replication.

### mtHSF1 causes neurodegeneration and HD‐like behavior

**Figure 5:**
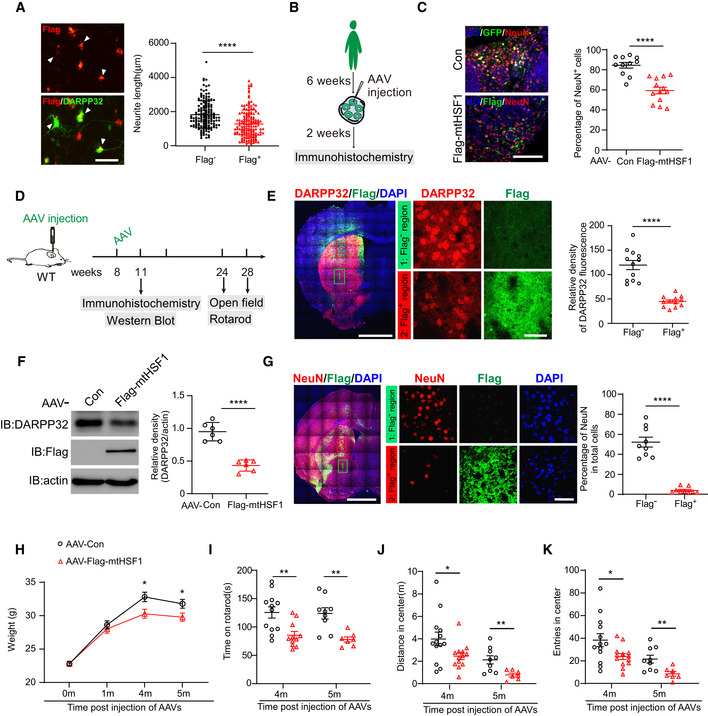
mtHSF1 causes neurodegeneration and HD‐like behavior APrimary neurons expressing Flag‐mtHSF1 were stained with anti‐DARPP32 (green) and anti‐Flag (red) antibodies. The arrows (white) mark Flag^+^ or Flag^‐^ primary neurons. The neurite length of Flag^+^ or Flag^‐^ neurons was measured by ImageJ and is shown by scatterplot. The scale bar represents 50 µm (*n* = 155 for Flag^‐^ group and *n* = 154 for Flag^+^ group).BSchematic diagram illustrating the timeline of viral injection into striatal organoids.CHuman striatal organoids were injected with AAV‐Con (GFP) or AAV‐mtHSF1 at D42. Scatterplot shows the percentage of NeuN^+^ cells (red) among Flag^+^ or GFP^+^ cells (green) after injection for 2 weeks. The data were obtained from 3 independent biological experiments. Con: *n* = 11 organoids; Flag‐mtHSF1: *n* = 13 organoids. The scale bar represents 100 µm.DTimeline overview of AAV injection and tests. Immunohistochemistry and WB analyses were performed 3 weeks post‐injection. Behavior was tested after 16 weeks or 20 weeks of AAV injection.ERepresentative images of immunostaining with DARPP32 (red) and Flag (green). The DARPP32 fluorescence density was analyzed in Flag^+^ or Flag^‐^ sections by confocal microscopy and is shown by scatterplot. Thickness: 30 µm. The scale bar is 2 mm, and the enlarged image scale bar is 40 µm (*n* = 12 mice/group).FThe striatum of AAV‐Con‐ or AAV‐mtHSF1‐injected mice was harvested. The DARPP32 protein levels were tested by WB analysis (*n* = 6 mice/group).GThe percentage of NeuN^+^ cells in Flag^+^ or Flag^‐^ sections was determined by confocal microscopy and is shown by scatterplot. Thickness: 10 µm. (*n* = 9 mice/group).H–KMouse weight and motor activity were recorded post‐AAV injection (*n* = 7–13 mice/group). Data information: The data are the means ± SEMs; (A–G) are from at least three independent biological experiments; unpaired Student’s *t*‐test was used in (A), (C), and (E–K). *P < 0.05, **P < 0.01, and ****P < 0.0001. Source data are available online for this figure.

Striatal MSN loss is a well‐established pathological change in HD patients and animal models (Reiner *et al*, 1988; Albin *et al*, 1990; Cowan *et al*, 2008; Zhang *et al*, 2008). To investigate the relevance of mtHSF1 to neurodegeneration, we expressed mtHSF1 in cultured primary MSNs and human striatal organoids with an AAV delivery system. Qualitative analysis showed that the expression of mtHSF1 shortened the neurites of primary neurons and reduced the numbers of NeuN^+^ cells in human striatal organoids (Fig 5A–C). Next, we injected AAV‐Con or AAV‐mtHSF1 into the striata of WT mice (Fig 5D). The immunofluorescence density of DARPP32 and the number of NeuN^+^ cells in the Flag^+^ area were greatly decreased compared with those in the adjacent Flag^‐^ area (Fig 5E and G). Immunoblot analysis consistently indicated that the protein levels of DARPP32 were reduced by approximately 50% in mtHSF1‐expressing mouse striatal extracts (Fig 5F). Furthermore, we evaluated the body weights of these mice and found that mtHSF1‐expressing mice were lighter than their littermates expressing the control vector (Fig 5H). Animal behavior was assessed via accelerating rotarod test and open‐field test. Sustained expression of mtHSF1 in WT mice resulted in defects in motor activity, which are critical features of HD in animals (Fig 5I–K). Altogether, these results suggest that mtHSF1 contributes to neuropathology and movement deficits in HD.

### DH1 reduces the association between HSF1 and mitochondria by interfering with Drp1/HSF1 binding

**Figure 6:**
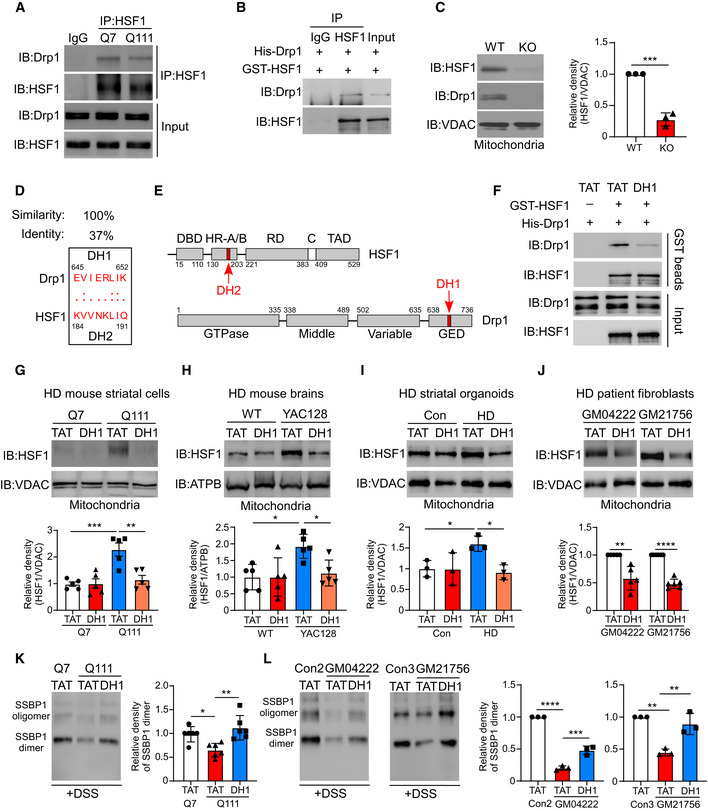
DH1 reduces association between HSF1 and mitochondria by interfering with Drp1/HSF1 binding ATotal lysates of HdhQ7 or HdhQ111 cells were subjected to immunoprecipitation with an anti‐HSF1 antibody followed by immunoblotting (*n* = 3 biological replicates).BDrp1 and HSF1 binding *in vitro* was analyzed by WB analysis (*n* = 3 biological replicates).CMitochondrial fractions were isolated from WT or Drp1‐KO MEFs. The protein levels of Drp1 and HSF1 were tested by immunoblotting (*n* = 3 biological replicates).DSequence analysis of Drp1 and HSF1 was performed with the L‐ALIGN sequence alignment software.ESketch of the domains of Drp1 and HSF1. DH1 in Drp1 and the corresponding region DH2 in HSF1 are marked in red.FGST pulldown assay of Drp1 and HSF1 in the presence or absence of DH1 (*n* = 3 biological replicates).G–J(G) HdhQ7 or HdhQ111 cells (1 μM/day, 3 days), (H) YAC128 or age‐matched littermates (3 mg/kg/day, 3 months), (I) striatal organoids differentiated from patient iPSCs or control iPSCs (1 μM/day, 7 days), and (J) patient fibroblasts (1 μM/day, 5 days) were treated with DH1 or TAT. mtHSF1 was measured by immunoblotting (*n* = 3–5 biological replicates).K, L(K) HdhQ7 or HdhQ111 and (L) patient fibroblasts were treated with DH1 or TAT (1 μM/day, 3 days). SSBP1 oligomers were analyzed by immunoblotting. (*n* = 3–6 biological replicates). Data information: The data are the means ± SEMs from at least three independent biological experiments; unpaired Student’s *t*‐test was used in C, and one‐way ANOVA followed by Tukey’s multiple comparison test was used in (G–L). *P < 0.05, **P < 0.01, ***P < 0.001, and ****P < 0.0001. Source data are available online for this figure.

Recent studies, including ours, have shown that hyperactivated Drp1 translocates to mitochondria in HD (Song *et al*, 2011; Reddy & Shirendeb, 2012; Guo *et al*, 2013; Zhao *et al*, 2019). P53, a nuclear transcription factor, is recruited to mitochondria via formation of a complex with Drp1 (Guo *et al*, 2014). These findings prompted us to hypothesize that Drp1 drives HSF1 translocation to mitochondria. To test our hypothesis, we performed coimmunoprecipitation (Co‐IP) analysis and found that Drp1 coprecipitated with HSF1 in striatal cells (Fig 6A). The interaction between HSF1 and Drp1 was confirmed in two lines of HD patient fibroblasts (GM04208 and GM04222) and in healthy control fibroblasts (Fig EV3I). GST pulldown assays revealed that recombinant GST‐HSF1 interacted with a recombinant His‐Drp1 protein directly (Fig 6B). Next, we examined whether Drp1 is required for HSF1 association with mitochondria. HSF1 levels in the mitochondria were almost nonexistent in Drp1‐knockout (KO) mouse embryonic fibroblasts (MEFs) (Fig 6C), whereas the total protein levels and nuclear protein levels of HSF1 were not affected (Fig EV3J). Conversely, transfection of Myc‐Drp1 into HEK293 cells strongly promoted HSF1 accumulation in mitochondria (Fig EV3K). Collectively, these data demonstrated that the mitochondrial fission protein Drp1 is required for HSF1 translocation to mitochondria.

**Figure EV4:**
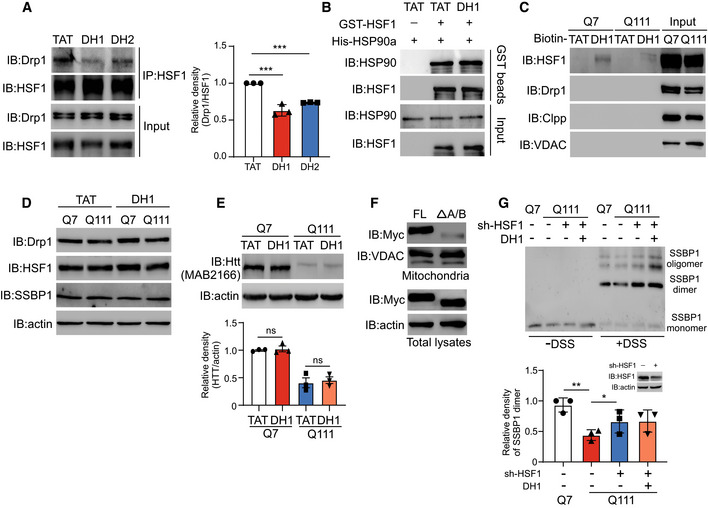
DH1 reduces binding of Drp1 and HSF1 AHdhQ111 cells were treated with TAT, DH1, or DH2 (1 μM, 3 days). Total proteins were extracted and subjected to immunoprecipitation with an anti‐HSF1 antibody. The immunoprecipitates were analyzed by WB analysis (*n* = 3 biological replicates). The data are the means ± SEMs; one‐way ANOVA followed by Tukey’s multiple comparison test was used. ***P < 0.001.BThe recombinant protein GST‐HSF1 was incubated with DH1 or TAT and then mixed with His‐HSP90a. The immunoprecipitates were analyzed by WB analysis (*n* = 2 biological replicates).CCell extracts were collected from HdhQ7 or HdhQ111 cells, treated with biotin‐labeled TAT or DH1 (10 μM), and incubated with streptavidin beads. The immunoprecipitates were analyzed by WB analysis (*n* = 2 biological replicates).D, EHdhQ7 or HdhQ111 cells were treated with TAT or DH1 (1 μM, 3 days). Immunoblot analysis was performed to examine the total levels of Drp1, HSF1, HTT, and SSBP1 (*n* = 3 biological replicates).FFlag‐HSF1 or Flag‐HSF1ΔA/B was transfected into HdhQ7 cells. Isolated mitochondria‐enriched fractions were analyzed by immunoblotting (*n* = 3 biological replicates).GHSF1‐knockdown cells or control cells were treated with DH1 or TAT (1 μM, 3 days). SSBP1 oligomers were examined by immunoblotting (*n* = 3 biological replicates). Data information: The data are the means ± SEMs; one‐way ANOVA followed by Dunnett's multiple comparisons test was used for (A); Tukey’s multiple comparison test was used for (E and G). *P < 0.05, **P < 0.01, and ***P < 0.001. Source data are available online for this figure.

To determine whether mtHSF1 is a potential target for the treatment of HD, we sought to develop a peptide inhibitor to reduce the association between HSF1 and mitochondria. Since Drp1 binds to HSF1 and promotes its mitochondrial translocation, blocking Drp1/HSF1 binding may reduce HSF1 accumulation in mitochondria. Accordingly, we carried out sequence analysis with L‐ALIGN sequence alignment software and identified one region of homology between Drp1 (human, NP_036192) and HSF1 (human, NP_005517) (Fig 6D). We hypothesized that this region may be required for Drp1/HSF1 binding and synthesized two peptides corresponding to the region, which were named DH1 and DH2 (Fig 6E). The amino acids of DH1 and DH2 were linked to the cell‐permeating TAT peptide, which is a part of the HIV1 protein transduction domain. We treated HdhQ111 cells with DH1 or DH2 and found that DH1 had a better inhibitory effect than DH2 on Drp1/HSF1 binding (Fig EV4A). Next, a GST pulldown assay confirmed that DH1 reduced the binding affinity of recombinant Drp1 and HSF1 proteins (Fig 6F). Importantly, DH1 had no effect on the binding affinity of HSF1/HSP90a, indicating the selectivity of DH1 (Fig EV4B). To assess the specificity of the peptide DH1, we performed a pulldown assay via incubation of biotin‐labeled DH1 or TAT with cell extracts. Biotin‐DH1 specifically bound to HSF1 but not to other proteins, such as VDAC, Clpp, and Drp1 (Fig EV4C). We used several complementary models to examine the role of DH1 in blocking the association between HSF1 and mitochondria. Treatment with DH1 reduced HSF1 translocation to mitochondria in HD striatal cells, YAC128 mice, human striatal organoids, and two lines of patient fibroblasts (Fig 6G–J), whereas the total protein levels of HSF1, Drp1, and HTT were not affected by the presence of DH1 (Fig EV4D and E). Given that DH1 corresponds to the A/B domain of HSF1, deletion of the A/B domain sharply reduced the association between HSF1 and mitochondria (Fig EV4F). Conceivably, blockade of HSF1 mitochondrial localization by DH1 rescued SSBP1 oligomerization (Fig 6K and L). Notably, the downregulation of HSF1 abolished the effect of DH1 on SSBP1 oligomer formation, indicating that HSF1 is required for mitochondrial protection of DH1 (Fig EV4G). Taken together, our results demonstrate that DH1 functions as a peptide inhibitor to suppress HSF1 mitochondrial localization and correct SSBP1 oligomer formation by interfering with Drp1/HSF1 binding.

### DH1 reduces mitochondrial dysfunction and neurotoxicity

**Figure EV5:**
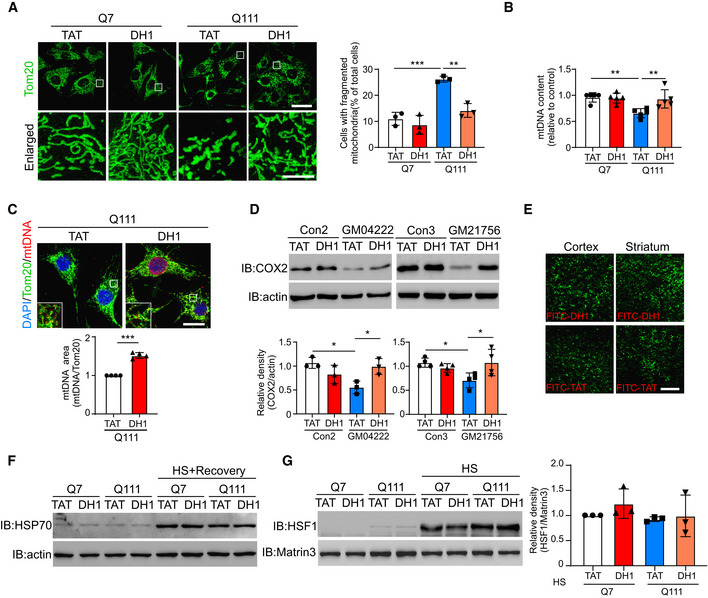
DH1 improves mitochondrial function AHdhQ7 or HdhQ111 cells were treated with TAT or DH1 for 3 days (1 μM). The cells were stained with an anti‐TOM20 antibody. Mitochondrial morphology was observed by microscopy. Scatterplot with bar shows the percentage of cells with fragmented mitochondria (*n* = 3 biological replicates). The scale bar represents 40 µm, and the enlarged image scale bar is 4 µm.B, CHdhQ7 or HdhQ111 cells were treated with TAT or DH1 for 5 days (1 μM). mtDNA content was measured by qPCR (*n* = 5 biological replicates) or immunostaining with an anti‐DNA antibody (*n* = 4 biological replicates) and is shown by scatterplot. The scale bar represents 20 μm.DPatient fibroblasts and control fibroblasts were treated with TAT or DH1 for 3 days (1 μM). COX2 protein levels were tested by WB analysis (*n* = 3–4 biological replicates).EFITC‐conjugated DH1 or TAT was injected intraperitoneally into WT mice (3 mg/kg, each) for 5 days. Brain sections were observed by microscopy. The scale bar is 100 µm.FHdhQ7 or HdhQ111 cells were treated with TAT or DH1 for 3 days (1 μM). The cells were cultured at 42°C for 1 h and then allowed to recover for 3 h. HSP70 levels was examined by immunoblotting (*n* = 2 biological replicates).GTAT‐ or DH1‐treated cells were cultured at 42°C for 1 h. Nuclear fractions were isolated and measured by WB analysis (*n* = 3 biological replicates). Data information: The data are the means ± SEMs from at least three independent biological experiments; one‐way ANOVA followed by Tukey’s multiple comparison test was used. *P < 0.05, **P < 0.01, and ***P < 0.001. Source data are available online for this figure.

**Figure 7:**
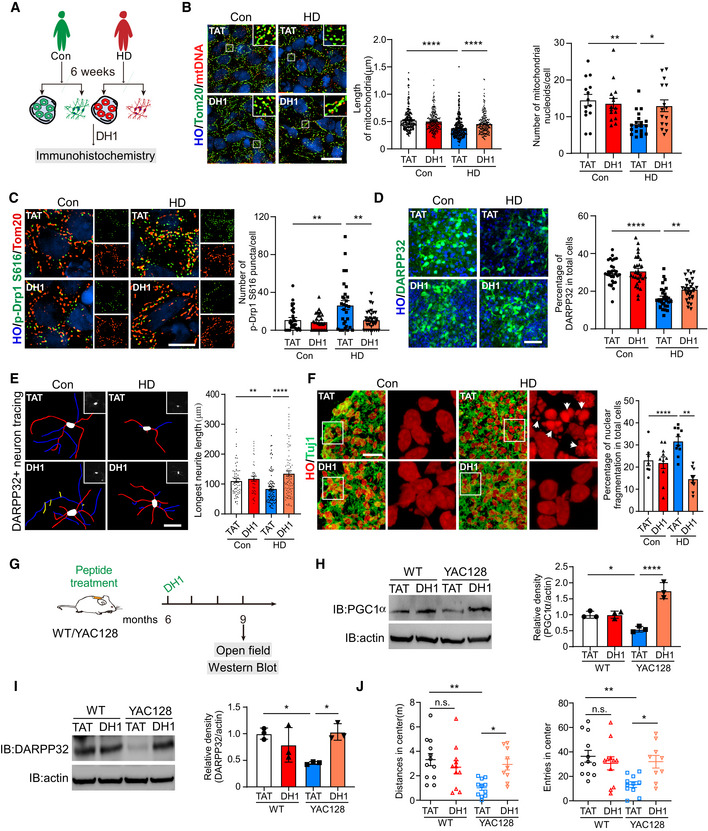
DH1 reduces mitochondrial dysfunction and neurotoxicity ASchematic showing the DH1 treatment procedure for striatal organoids.BImmunostaining for mitochondria (Tom20, green) and mtDNA (anti‐DNA, red) in striatal organoids treated with DH1 or TAT at D60. Scatterplots with bar show the length of mitochondria and the number of mtDNA colocalized with mitochondria. The data were obtained from 3 independent biological experiments. Left, *n* = 222–311 mitochondria per group; right, *n* = 14–20 organoids per group. Images were taken using structured illumination microscopy (SIM). The scale bar represents 10 μm.CImmunostaining for mitochondria (Tom20, red) and p‐Drp1 S616 (green) in striatal organoids treated with DH1 or TAT at D60. Scatterplot with bar shows the number of p‐Drp1 S616 per cell in organoids. The data were obtained from 3 independent biological experiments. *n* = 29–34 cells from at least 10 organoids. The scale bar represents 5 μm.DScatterplot with bar shows the ratio of MSNs (DARPP32, green) in striatal organoids in the presence or absence of DH1. The data were obtained from 3 independent biological experiments. *n* = 30 organoids per group. The scale bar represents 50 μm.ERepresentative tracing images of DARPP32^+^ neurons treated with DH1 or TAT at D45. Scatterplot with bar shows the results of longest neurite length. The data were obtained from 3 independent biological experiments. *n* = 45–92 neurons per group. The scale bar represents 20 μm.FRepresentative images and scatterplot with bar of nuclear fragmentation in striatal organoids treated with DH1. The arrows (white) mark fragmented nuclei. The data were obtained from 3 independent biological experiments. *n* = 7–10 organoids per group. The scale bar represents 20 μm.GSchematic showing the DH1 treatment procedure for WT/HD YAC128 mice.H, I(H) PGC1α and (I) DARPP32 protein levels were determined by immunoblotting (*n* = 3 biological replicates).JMouse behavior was tested after DH1 treatment (*n* = 9–12 mice/group). Data information: The data are the means ± SEMs from at least three independent biological experiments; one‐way ANOVA followed by Tukey’s multiple comparison test was used. *P < 0.05, **P < 0.01, and ****P < 0.0001. Source data are available online for this figure.

Given that mtHSF1 elicited mitochondrial fragmentation and mtDNA deletion, we hypothesized that blocking the association between HSF1 and mitochondria should improve abnormal mitochondrial events. Compared with HdhQ111 cells treated with TAT, HdhQ111 cells exposed to DH1 exhibited elongated mitochondria (Fig EV5A). qPCR analysis or immunostaining assays indicated that treatment with DH1 rescued the mtDNA content (Fig EV5B and C). The levels of cytochrome c oxidase subunit II (COX2), a subunit of mitochondrial complex IV encoded by mtDNA, were elevated by DH1 in two lines of HD patient fibroblasts (Fig EV5D). We further excluded the possibility that DH1‐mediated improvement of mitochondrial function depends on HSF1 nuclear translocation and HSF1 transcriptional activation (Fig EV5F and G). To investigate the effects of DH1 in human tissues, we generated striatal organoids from HD and control iPSCs (Fig 7A). Consistent with previous observations in mtHSF1‐overexpressing striatal organoids, we observed reduced mitochondrial lengths accompanied by increased p‐Drp1 S616 levels in HD striatal organoids (Fig 7B and C). Importantly, these mitochondrial abnormalities were corrected by the presence of DH1 (Fig 7B and C). In addition, treatment with DH1 increased the number of mitochondrial nucleoids, suggesting that DH1 improves mtDNA replication (Fig 7B). Notably, the proportion of DARPP32^+^ neurons was reduced in HD striatal organoids (Fig 7D), which reflects the typical phenotype of HD with a diminished MSN population in the striatum (Creus‐Muncunill & Ehrlich, 2019). With DH1 treatment, the percentage of DARPP32^+^ neurons was increased in HD striatal organoids (Fig 7D). Moreover, the neurite length of DARPP32^+^ neurons was augmented by the presence of DH1 (Fig 7E). The neuroprotective effect of DH1 was also confirmed via quantification of neurons with typical fragmented nuclear morphology (Fig 7F). Next, we implanted osmotic pumps delivering DH1 or TAT into the backs of mice (Fig 7G). FITC‐conjugated DH1 was observed in the cortex and striatum, suggesting that DH1 crosses the blood–brain barrier (BBB) (Fig EV5E). The levels of PGC1α, a key regulator of mitochondrial biogenesis, were elevated in YAC128 mice treated with DH1 (Fig 7H). DARPP32 levels in the YAC128 mice were rescued by the presence of DH1 (Fig 7I). Of note, continuous treatment with DH1 improved motor deficits, as tested by open‐field test (Fig 7J). Therefore, our results demonstrate that DH1 slows neurodegeneration by restoring mitochondrial function.

## Discussion

The malfunction of heat shock response has been reported in various HD models. However, recent studies including ours show that the total protein levels of HSF1 are not changed between HD models and their controls, suggesting that mtHtt‐suppressed heat shock response is not caused by impairing the stability of HSF1 (Labbadia *et al*, 2011; Gomez‐Paredes *et al*, 2021). mtHtt disrupts the binding affinity of HSF1 and the promoters of downstream genes, which may contribute to the pathogenesis of HD (Labbadia *et al*, 2011). Multiple lines of evidence have demonstrated that HSF1 is involved in the regulation of mitochondrial function. HSF1‐KO mice exhibit perturbed chaperone expression, enhanced O_2_^‐^generation, and mitochondrial protein oxidation (Yan *et al*, 2002, 2005). Oxidative adenine nucleotide translocase 1 (ANT1) promotes mitochondrial permeability transition pore (mPTP) opening (Yan *et al*, 2002). Under fasting conditions, deletion of HSF1 disrupts mitochondrial biogenesis by controlling acetylation of the nuclear transcription factor PGC1α (Qiao *et al*, 2017). A recent study has shown that HSF1 plays a role in mitochondria‐localized histone protein H4‐mediated mtDNA transcription (Sural *et al*, 2020). These findings indicate that constitutive HSF1 functions as a regulator of mitochondria. In the present study, we elucidated a novel mechanism of mitochondrial regulation by determining the direct association between HSF1 and mitochondria in HD.

Mitochondrial morphology is closely associated with neuronal cell survival in HD (Shirendeb *et al*, 2011; Hwang *et al*, 2015). Drp1 GTPase activity is triggered by mtHtt, which in turn accumulates in mitochondria, resulting in mitochondrial fragmentation (Shirendeb *et al*, 2012; Guo *et al*, 2014; Sawant *et al*, 2021). Hyperactivated Drp1 drives P53 translocation to mitochondria, which is repressed by the peptide inhibitor P110 (Guo *et al*, 2014). Downregulation of P53 abolishes the protective effects of P110 on mitochondria, suggesting that Drp1 cooperates with P53 to induce mitochondrial dysfunction (Guo *et al*, 2014). Our data indicate that Drp1 interacts with HSF1 and forces its mitochondrial localization. Interestingly, mtHSF1 sharply increased Drp1 phosphorylation at S616 in mitochondria, which was partly blocked by the CDK inhibitor roscovitine (Appendix Fig S1). Increases in CDK5‐mediated Drp1 phosphorylation at S616 in mitochondria accelerate mitochondrial fission (Jahani‐Asl *et al*, 2015). mtHSF1 may thus promote CDK5‐mediated Drp1 phosphorylation. Increasing lines of evidence have shown that mtDNA deletion contributes to the pathogenesis of HD (Horton *et al*, 1995; Disatnik *et al*, 2016). Deletion of SSBP1 causes rapid reductions in mtDNA levels, indicating that SSBP1 is a master regulator that maintains mtDNA content. A previous study has indicated that the binding of SSBP1 and HSF1 in the nucleus is required for chaperone expression under heat shock (Tan *et al*, 2015). Our results suggest that HSF1 interacts with SSBP1 on mitochondria and impairs SSBP1 oligomerization, which in turn reduces mtDNA content. The lysine at amino acid 188 (K188) of HSF1 is required for the interaction between HSF1 and SSBP1. It will be of interest to investigate whether the mtHSF1‐K188R could abolish the effect of HSF1 on mtDNA replication. As an interactor of HSF1, P53 directly regulates COX I in a manner dependent on its transcriptional activity (Qi *et al*, 2011). In addition, our proteomics data reveal that mtHSF1 may interact with DNA‐binding proteins such as Top1mt, Hif3a, Rps3, and Parp1. Thus, the role of HSF1 in controlling the mtDNA expression in HD should be more carefully investigated in future studies.

Although comprehensive mechanistic studies have been performed in animal models, species differences may have impeded further translational studies (Shi *et al*, 2017; Chiaradia & Lancaster, 2020). Thus, a dynamic human brain model is needed to facilitate human neurological disease research. Breakthroughs have been made in generating human brain organoids from hPSCs in recent years, providing powerful tools for studying the etiology of neurological disorders (Lancaster *et al*, 2013; Amin & Pasca, 2018). The majority of reported studies have performed in cerebral organoids (Lancaster *et al*, 2017; Gonzalez *et al*, 2018). However, we developed a novel method to generate striatal organoids for exploring the pathogenesis of HD in this study. Expression of mtHSF1 caused neurodegeneration in the 3D striatal organoids and in mice. Movement deficits were found in mice expressing mtHSF1. Our data indicate that mtHSF1 and HD pathology are closely related. To our knowledge, inhibitors of HSF1 (e.g., KRIBB11) block the induction of HSP27 and HSP (Yoon *et al*, 2011). Genetic inhibition of HSF1 cannot exclude the function of HSF1 in transcriptional activation. We developed the peptide inhibitor DH1, which reduces HSF1 translocation to mitochondria by competing with HSF1 for binding with Drp1. Importantly, treatment with DH1 improved neurotoxicity and animal behavior, indicating that blocking HSF1 translocation to mitochondria may slow the pathogenesis of HD. Indeed, our results reveal an unsuspected role of HSF1 in mitochondria independent of its transcriptional activity, which may provide a therapeutic target for HD.

## Materials and Methods

### Reagents and antibodies

3‐NP (N5636) was purchased from Sigma‐Aldrich. A protease inhibitor cocktail (P001) was obtained from NCM Biotech. Lipofectamine 2000 Transfection Reagent (11668019) was purchased from Invitrogen. Disuccinimidyl suberate (DSS, C100015‐0100) was purchased from Sangon Biotech. The primary and secondary antibodies are listed in Appendix Table S2.

### Cell culture

HEK293 cells were obtained from the American Type Culture Collection (ATCC). Drp1‐WT and Drp1‐KO MEFs were gifts from Dr. Hiromi Sesaki (Johns Hopkins University). These cells were maintained in Dulbecco's modified Eagle's medium (DMEM) supplemented with 10% (v/v) fetal bovine serum (FBS) and 1% (v/v) penicillin/streptomycin.

HD patient fibroblasts (GM04208, GM04222, and GM21756) purchased from the Coriell Institute (USA), and normal fibroblasts (Con1 [C12302] and Con2 [C‐12300] cells were purchased from Sigma‐Aldrich; Con3 [HDF] was gifted from Dr. Lixiang Ma’s lab) were maintained in Eagle's Minimum Essential Medium (MEM) with nonessential amino acids (NEAAs), 15% (v/v) FBS, and 1% (v/v) penicillin/streptomycin.

For primary neuron culture, cover slides were incubated with poly‐D‐lysine (100 µg/ml) and laminin (15 µg/ml) overnight at 37°C. Primary striatal neurons were isolated from embryonic day (E) 16‐E17 mouse brains and cultured on cover slides in neurobasal medium containing 2% B27 and 0.5 mM glutamate.

hPSCs, including H9 (WiCell Agreement No. 16‐W0060), IMR90‐4 (WiCell Agreement No. 17‐W0063), and HDUE003 (gift from Dr. Guangjin Pan's lab) iPSCs that were derived from urothelial cells collected from a HD patient with 75 CAG repeats, were maintained under feeder‐free conditions as previously described (Liu *et al*, 2013; Yuan *et al*, 2018). The cells were cultured on vitronectin‐coated plates in Essential 8 (E8) medium (Life Technologies) and passaged every 5–7 days with 1 ml of EDTA (STEMCELL Technologies). Half of the medium was replaced every day.

All of the above cells were kept at 37°C in a 5% CO_2_ incubator.

WT striatal ST HdhQ7/Q7 cells (CHDI‐9000073) and mutant striatal ST HdhQ111/Q111 cells (CHDI‐9000071) were purchased from the Coriell Institute. The cells were cultured with high‐glucose DMEM containing 10% (v/v) FBS, 1% (v/v) penicillin/streptomycin, and 400 μg/ml geneticin. The cells were grown in a 33°C incubator with 5% CO_2_.

### Cell death assay

For the cell death assay, the indicated vectors were transfected into cells with Lipofectamine 2000. The medium was harvested to measure LDH release using an LDH Cytotoxicity Detection Kit (Takara, MK401) following the manufacturer’s instructions. The cell lysates were collected, and cleaved PARP was tested by WB analysis with the indicated antibody. To examine nuclear fragmentation, cells in the outer layer of the organoids, which are almost neurons, were selected. The nucleus with obvious dot fragments and higher fluorescence intensity was defined as a fragmented nucleus.

### Isolation of mitochondria‐enriched fractions

Mitochondria‐enriched fractions were obtained as previously described (Guo *et al*, 2014, 2016). Briefly, cells in 10‐cm dishes that had reached approximately 90% confluence were washed with PBS and lysed with mitochondrial isolation buffer (250 mM sucrose, 20 mM HEPES‐NaOH [pH 7.9], 10 mM KCl, 1.5 mM MgCl2, 1 mM EDTA, 1 mM EGTA, protease inhibitor cocktail and phosphatase inhibitor cocktail) on ice for 30 min. Mouse brains were minced and homogenized in the lysis buffer and then placed on ice for 30 min. Collected cells or tissues were transferred to tubes and then disrupted 20 times by repeated aspiration through a 25‐gauge needle. The homogenates were centrifuged at 800 *g* for 10 min at 4°C. Subsequently, the supernatants were spun at 12,000 *g* for 20 min at 4°C. The pellets (mitochondria‐enriched fractions) were washed with mitochondrial isolation buffer and centrifuged at 12,000 *g* for 20 min at 4°C before being dissolved in mitochondrial isolation buffer containing 1% Triton X‐100.

### Real‐time PCR

Total RNA was isolated using RNA simple Total RNA Kit (DP419, Tiangen). cDNA was synthesized by reverse transcription using 0.5μg of total RNA with HiScript III RT SuperMix for qPCR (+gDNA wiper) (R323‐01, Vazyme), and was diluted 10‐fold for real‐time analysis. Quantitative real‐time PCR was followed by AceQ qPCR SYBR Green Master Mix (Q111‐02, Vazyme) on an Applied Biosystems™ QuantStudio™ 3 Real‐Time PCR System (Thermo Fisher Scientific). The results were normalized by GAPDH. The following primers were used for real‐time PCR: mouse HTT sense: 5′‐AGGGAGGAAGGAGCCAAAATC‐3′; mouse HTT antisense: 5′‐AGGGAGGAAGGAGCCAAAATC‐3′; mouse GAPDH sense: 5′‐TGGCCTTCCGTGTTCCTAC‐3′; and mouse GAPDH antisense: 5′‐GAGTTGCTGTTGAAGTCGCA‐3′.

### Plasmids and transfection

Full‐length HSF1 and HSF1‐ΔA/B plasmids were generated by inserting PCR‐amplified fragments into pcDNA3.1(+)‐Flag. Myc‐Drp1 was created by inserting PCR products into pCMV‐Myc. Flag‐mtHSF1 was constructed by fusing the mitochondrial targeting sequence with the N‐terminus of HSF1. Httex1‐Q23 and Httex1‐Q73 were obtained from the Coriell Institute. His‐Drp1 was subcloned into the pET28a vector. Cells were transfected with Lipofectamine 2000 (Invitrogen) following the manufacturer's instructions.

### shRNAs and viral packing

The pLKO.1‐puro control vector (SHC001) was purchased from Sigma‐Aldrich. sh‐HTT, sh‐HSF1, and sh‐SSBP1 were cloned by inserting the effective sequences listed below into the pLKO.1‐puro control vector.

sh‐HTT: sense, 5′‐CCGGCCTCCAGTACAAGACTTTATTCTCGAGAATAAAGTCTTGTACTGGAGGTTTTTG‐3′;

sh‐HSF1: sense, 5′‐CCGGGCTGCATACCTGCTGCCTTTACTCGAGTAAAGGCAGCAGGTATGCAGCTTTTTTT‐3′; and sh‐SSBP1: sense, 5′‐CCGGGCCAAGGCATACATCTGGAAACTCGAGTTTCCAGATGTATGCCTTGGCTTTTTTG‐3′.

The packaging plasmid pMDLg/pRRE and pRSV‐Rev and the envelope plasmid pCMV‐VSVG were obtained from Addgene. For lentivirus production, 5 μg of shRNA vector, 2.5 μg of pMDLg/pRRE, 1.25 μg of pRSV‐Rev, and 1.5 μg of pCMV‐VSVG were transfected into HEK293 cells with Lipofectamine 2000. The viral supernatant was harvested 72 h after transfection, filtered with a 0.45‐μm filter, and concentrated with 5× PEG. For viral infection, cells were incubated with lentivirus and polybrene (10 μg/ml). Forty‐eight hours later, the knockdown cell lines were selected with puromycin (4 μg/ml).

### Immunoprecipitation

Cells were harvested by lysis with HEPES buffer (20 mM HEPES [pH 7.2], 50 mM NaCl, and 0.5% Triton X‐100) on ice. The extracts were centrifuged at 12,400 *g* for 15 min at 4°C. One milligram of protein was incubated with the indicated primary antibodies or IgG overnight at 4°C and then incubated for 1 h with protein A/G beads (sc‐2003, Santa Cruz Biotechnology). The immunoprecipitates were washed with HEPES buffer three times and then immunoblotted with antibodies. Striatal cells were harvested with RIPA buffer (50 mM Tris [pH 7.4], 150 mM NaCl, 1% Triton X‐100, 1% sodium deoxycholate, and 0.1% SDS). The protein supernatant was incubated with biotin‐DH1(10 μM) or biotin‐TAT (10 μM) for 12 h and then with streptavidin beads for 1 h.

### Generation of striatal organoids from hPSCs

To generate striatal organoids, hPSCs were detached with dispase (Life Technologies) and washed with DMEM/F12 medium (Life Technologies). Then, the cells were transferred into flasks to form embryoid bodies (EBs) with half E8 medium and half neural induction medium (NIM; DMEM/F12, 1% N2, and 1% NEAAs, Life Technologies). The medium was supplemented with the SMAD inhibitor SB431542 and the BMP receptor inhibitor DMH1, and half of the medium was changed every day. On day 7, the suspended EBs were attached to 6‐well plates with NIM containing 10% FBS (Life Technologies). Neural tube‐like rosettes were observed on day 10. On day 16, the differentiating rosettes were gently blown off with a 1‐ml pipette and transferred into flasks with fresh NIM containing 2% B27 to form brain organoids. Sonic hedgehog (SHH, R&D) at 20–200 ng/ml was used from days 10–25 for striatum induction according to the methods described previously for our 2D culture system (Ma *et al*, 2012). The organoids were dissociated into single cells with TrypLE (Life Technologies), and approximately 3 × 10^4^ cells were seeded on Matrigel‐coated coverslips in a 24‐well plate. The striatal organoids were fixed with 4% paraformaldehyde in 1.5 ml EP tubes for 2–4 h at 4°C for immunohistological analysis. The organoids were cut into 10‐μm sections using a Leica Cryostat (CM1950).

### scRNA‐seq and data analysis

scRNA‐seq was performed on day 30 and day 60. To obtain a single‐cell suspension, 5–7 organoids were randomly selected into a 1.5‐ml EP tube with 1ml of TrypLE (Life Technologies) and incubated in a 37°C incubator for 40 min with gentle agitation every 5–8 min. The organoids were then washed with DPBS (Life Technologies) 3 times and gently dissociated into single cells with a 200‐µl pipette. Each single‐cell suspension was loaded onto a Chromium single‐cell controller (10x Genomics). The captured cells were lysed, and the released RNA was barcoded through reverse transcription in individual GEMs. scRNA‐seq libraries were constructed according to the manufacturer's instructions using a Single Cell 3′ Library and Gel Bead Kit V3 (10× Genomics). Normalization and clustering of single cells were performed with the R package Seurat 3.0. Unbiased clustering was performed by principal component analysis (PCA), and dimensionality reduction visualization was performed via t‐distributed stochastic neighbor embedding (TSNE) and UMAP.

### Viral injection into striatal organoids

Viral injection into the striatal organoids was performed from D42‐D46, and the viruses used in this study were ST‐98 (rAAV‐Ef1a‐EYFP‐WPRE‐pA) and CT‐12 (rAAV‐Ef1a‐HSF‐flag‐WPRE‐pA). The viral titer was 1 × 10^13^. Before injection, 10 organoids were transferred to droplets of 20 μl of medium in a 35‐mm dish. Every organoid was injected with 0.2 μl of virus suspension with a capillary tube (World Precision Instruments, TW100‐4) and incubated at 37°C and 5% CO_2_ for 30 min. Then, the organoids were transferred into a flask with fresh NIM containing 2% B27 and 1% penicillin/streptomycin.

### Immunohistochemistry and morphological analysis

Sections or attached cells were washed three times with PBS. The detailed procedures for different sections/cells are listed in Appendix Table S3. Images were randomly taken with a TS100 microscope (Nikon), an LSM 880 microscope with Airyscan (Carl Zeiss), or an N‐SIM Structured Illumination Microscope (Nikon). The data were analyzed with ImageJ and GraphPad.

To examine mitochondrial length, mitochondrial morphology was imaged by structured illumination microscopy (N‐SIM, Nikon), using 100x oil immersion lens. The neurons in the outer layer of the organoids were selected. For each image, about 50–100 mitochondria in a random selected (15 × 15 μm) area were analyzed. The freehand tool in ImageJ was used to manually trace the maximum length or the diameter of each mitochondrion.

To quantify neurite length, we employed Neuroanatomy (Fiji) software to trace DARPP32‐positive neurites. Tracings were color‐coded: red for primary neurite (extension directly from the soma), blue for secondary neurite (branching from a primary), and yellow for tertiary neurite (branching from a secondary).

To quantify the percentage of NeuN‐positive cells over total cells (% Neu+), fields in the outer layer of the organoids were randomly captured. Around 10 organoids (11 organoids in con vs 13 organoids in Flag‐mtHSF1) from three independent differentiation were randomly selected. GraphPad Prism version 9.0.0 was used for statistical analyses.

### Lysate preparation and WB analysis

Protein extracts were prepared in lysis buffer (50 mM Tris–HCl [pH 7.5], 150 mM NaCl, 1% Triton X‐100 and protease inhibitor). The protein concentrations were measured by BCA assay (P0009, Beyotime Biotechnology). The proteins were subjected to SDS–PAGE and incubated with the indicated antibodies.

### Measurement of mtDNA content

Total DNA was purified using a TIANamp Genomic DNA Kit (DP304, Tiangen Biotech Co., Ltd.). The nuclear DNA (GAPDH or Tert) and mtDNA (mtCO1, mtCO2, mtND2, and D‐loop) were analyzed in triplicate by qPCR under standard conditions using the AceQ qPCR SYBR Green Master Mix (Q121‐02, Vazyme). The mtDNA abundance was calculated using the delta Ct (ΔCt) of average Ct of mtDNA and nDNA (ΔCt = CtmtDNA‐CtnDNA) in the same well as 2^−ΔCT^. The following primers were used in this study: GAPDH sense, 5′‐GGACCTCATGGCCTACATGG‐3′; GAPDH antisense, 5′‐TAGGGCCTCTCTTGCTCAG‐3′; Tert sense, 5′‐CTAGCTCATGTGTCAAGACCCTCTT‐3′; Tert antisense, 5′‐GCCAGCACGTTTCTCTCGTT‐3′; D‐loop sense, 5′‐CCCTTCCCC ATTTGGTCT‐3′; D‐loop antisense, 5′‐TGGTTTCACGGAGGATGG‐3′; mtCO1 sense, 5′‐CTGAGCGGGAAT AGTGGGTA‐3′; mtCO1 antisense, 5′‐TGGGGCTCCGATTATTAGTG‐3′; mtCO2 sense, 5′‐TAGGGCACCAATGATACTGAAG‐3′; mtCO2 antisense, 5′‐CTTCTAGCAGTCGTAGTTCACC‐3′; mtND2 sense, 5′‐AACCCACG ATCAACTGAAGC‐3′; and mtND2 antisense, 5′‐TTGAGGCTGTTGCTTGTGTG‐3′.

### Peptide design and synthesis

The peptides were designed with a similar approach to those used for P110 (Qi *et al*, 2013) and HV3 (Guo *et al*, 2016). DH1 or DH2 was linked to the cell‐permeating peptide TAT to facilitate peptide passage across the cell membrane and BBB. The purity was assessed as >99% by HPLC analysis. The peptides were stored in the form of lyophilized powder and dissolved in sterile water for use.

### *In vitro* binding assay

For the *in vitro* binding study, purified His‐Drp1 and the recombinant protein GST‐HSF1 (500 ng each) were mixed in HEPES buffer for 1 h at RT. The mixture was incubated with an HSF1 antibody overnight at 4°C and then with protein A/G beads for 1 h. For DH1 analysis, GST‐tagged HSF1 was incubated with TAT or DH1 in TBST buffer (2.42 g Tris base, 8 g NaCl, H2O to 1,000 ml, 1/1,000 Tween‐20) at RT for 1 h and then with His‐tagged proteins (His‐Drp1 or His‐HSP90a, respectively). The mixture was incubated with glutathione beads (GE Healthcare, 17‐0756‐01) overnight at 4°C. The immunoprecipitates were then washed three times with GST binding buffer (PBS [pH 7.3], 140 mM NaCl, 2.7 mM KCl, 10 mM Na2HPO4, and 1.8 mM KH2PO4 [pH 7.3]) and subjected to WB analysis.

### Mass spectrometry analysis

Empty vector‐ or mtHSF1‐expressing striatal cells were lysed and centrifuged. The supernatant was incubated with an HSF1 antibody and then with protein A/G beads. The beads were washed with HEPES buffer three times. The samples were boiled and separated by SDS–PAGE, and then, the gel was sent to the mass spectrometry facility at the Nanjing Medical University for analysis.

### Mitochondrial respiration measurements

Control and mtHSF1‐expressing cells were seeded in XF96 culture plates at a density of 8,000 cells per well in 100 μl of medium. Mitochondrial respiration activity was measured using a Seahorse XF96 instrument (Agilent) as previously described (Zhao *et al*, 2019). Oligomycin (1 μg/ml), carbonyl cyanide 4‐(trifluoromethoxy) phenylhydrazone (FCCP, 1 μM), and rotenone and antimycin (0.5 μM each) were sequentially added to measure basal respiration, maximal respiration, and ATP production.

### Animals and stereotactic injection

All animals were maintained under a 12‐h light/dark cycle. The experiments were carried out according to the standard protocols and were approved by the Animal Care and Use Committee at the Nanjing Medical University (IACUC2010029). Male YAC128 mice were obtained from the Jackson Laboratory (B6.FVB‐Tg [YAC128] 53 Hay/ChdiJ; JAX stock number: 027432). The mice were anesthetized and injected with 3 μl of rAAV‐Ef1a‐mito‐HSF‐Flag‐WPRE‐pA at two sites in the striatum (bregma: AP: 0.5 mm, ML: 2/−2 mm, DV: −3.5 mm). Microinjection was performed by using a stereotactic instrument (RWD, China). The mice were placed on a prewarmed blanket until they were fully awake. The animals were perfused and postfixed with 4% paraformaldehyde (PFA, Sigma). Coronal sections of 10 μm/30 μm were obtained using Leica microtome (SM2010R) or Leica Cryostat (CM1950).

### Systemic peptide treatment in HD mice

All randomization and peptide treatments were performed by an experimenter not associated with the behavioral and neuropathological analyses. Hemizygous YAC128 male mice (Tg) and their age‐matched WT littermates (6 months old) were implanted with a 42‐day osmotic pumps (Alzet, Cupertino CA, 2006) containing the TAT control peptide or the DH1 peptide, which was delivered to each mouse at a rate of 3 mg/kg/day. The pump was implanted subcutaneously in the back of each 6‐month‐old mouse between the shoulders. The pump was replaced every 6 weeks until the treatments were terminated by the age of 9 months, and the brain tissue was harvested for analysis.

### Behavioral analysis in HD mice

All behavioral analyses were conducted by an experimenter who was blinded to the genotypes and treatment groups. Locomotor activity was assessed in YAC128 mice and age‐matched WT littermates at the age of 9 months using an open‐field test. The mice were individually placed in a corner of an open field (50 cm × 50 cm × 50 cm) and allowed to explore freely for 10 min. The movement of the mouse was tracked via video tracking software (ANY‐maze, Stoelting). The distance traveled and time spent in the central area of the open field were analyzed. The motor coordination and balance of WT mice that were injected with AAV‐mtHSF1 were tested on an accelerating rotarod (RWD, YLS‐4C) at the ages of 24 and 28 weeks, respectively. For training, mice were given three 180‐s trials per day at a fixed speed of 5 rpm for three consecutive days. During the testing phase, the rotarod accelerated from 5 to 40 rpm over 90 s; the maximum score was 180 s. The rotarod score was calculated as the average of the scores in three trials per day (with 2 h of rest between trials) for 3 consecutive days. The behavioral data were analyzed with Student's *t*‐test or one‐way ANOVA.

### Transmission electron microscopy

Mice were perfused with 4% PFA. First, the striata were acquired quickly after the brain samples were removed from the skulls and fixed in 2.5% glutaraldehyde at 4°C for 2 h. Then, the samples were washed four times in PBS. After that, they were postfixed in 1% osmic acid at 4°C for 2 h and then incubated in the 2% uranyl acetate aqueous solution for next 2 h. Following dehydration in a conventional acetone gradient from 50 to 100% at 4°C for 15 min, the samples were placed in a mixture of 100% dehydrating agent and the same amount of Epon 812 embedding agent at RT for 2 h. Subsequently, the samples were soaked in pure embedding agent at RT overnight. When the embedding agent had solidified, the blocks were cut into 50‐ to 80‐nm ultrathin sections. After staining with uranyl acetate and lead citrate, the ultrathin sections were examined under a JEOL JEM‐1400 Flash TEM microscope. The length of mitochondria was measured by the NIH ImageJ software.

### Immunoelectron microscopy

HD striatal organoids at D50 were fixed in 0.1% glutaraldehyde in 4% PFA; then, they were embedded with 10% gelatin and infiltrated with 2.3 M sucrose for 48 h. The embedded HD striatal organoids were frozen for ultrathin sectioning (Leica FC7) at a thickness of 70 nm. A nickel mesh was used to transfer the sections to carbon‐coated nickel grids on a microscope slide. The sections were blocked with 0.1% glycine (w/v) in PBS and 1% BSA (w/v) in PBS. After blocking, the sections were incubated with or without an anti‐HSF1 antibody in incubation buffer containing a blocking reagent (e.g., 0.1% BSA [w/v]) in PBS and then incubated with 10 nm gold‐conjugated goat anti‐rabbit IgG as a secondary antibody (Abcam, ab27234). Finally, the sections were imaged under an FEI Tecnai Spirit TEM.

### Statistical analysis

The data were analyzed by Student's *t*‐test for comparison of two groups or ANOVA followed by Tukey’s multiple comparison test for comparison of multiple groups. Each study was performed with at least three independent biological replications. The data are expressed as the means ± SEMs. Statistical significance was considered achieved when the value of *P* was < 0.05.

## Author contributions

**Xing Guo:** Supervision; Funding acquisition; Investigation; Writing—original draft; Project administration; Writing—review & editing. **Chunyue Liu:** Investigation. **Zixing Fu:** Investigation. **Shanshan Wu:** Methodology. **Xiaosong Wang:** Investigation. **Chu Chu:** Investigation. **Yuan Hong:** Investigation. **Yueqing Jiang:** Resources; Methodology; Project administration. **Yang Wu:** Investigation; Methodology. **Yongbo Song:** Methodology; Project administration. **Yan Liu:** Funding acquisition; Writing—original draft; Project administration; Writing—review & editing. **Shengrong Zhang:** Investigation. **Shengqi Chen:** Investigation. **Wenbo Wu:** Investigation.

In addition to the CRediT author contributions listed above, the contributions in detail are:

ZXF, XSW, SRZ, WBW, SQC, and YQJ performed all experiments in cell cultures. CYL and SSW maintained mice, injected AAVs, performed DH1 treatments and biochemical analyses of animal brains, and conducted animal behavioral analyses. YW prepared AAVs. YBS purified the His‐Drp1 protein. YH analyzed the single‐cell sequencing data. SSW and CC differentiated human striatal organoids *in vitro*, performed viral injection experiments, and performed immunostaining analysis. XG and YL conceived, designed, and supervised all the studies and wrote the manuscript.

## Disclosure and competing interests statement

The authors declare that they have no conflict of interest.

## Supporting information



Appendix

Expanded View Figures PDF

Source Data for Expanded View and Appendix

Source Data for Figure 1

Source Data for Figure 2

Source Data for Figure 3

Source Data for Figure 4

Source Data for Figure 5

Source Data for Figure 6

Source Data for Figure 7

## Data Availability

The scRNA‐seq data have been deposited to the GEO database with accession number GSE198927 (https://www.ncbi.nlm.nih.gov/geo/query/acc.cgi?acc=GSE198927).
