# Supplementary material for: Mitochondrial HSF1 triggers mitochondrial dysfunction and neurodegeneration in Huntington's disease
Source: EMBO Mol Med. 2022 Jun 7;14(7):e15851. doi: 10.15252/emmm.202215851 (PMC9260212; doi:10.15252/emmm.202215851)
Supplement: Supplementary file 1 — Appendix [file EMMM-14-e15851-s010.pdf]

Table of Content

1.Appendix Figure S1

2.Appendix Figure Legand

3.Appendix Table S1

4.Appendix Table S2

5.Appendix Table S3

1. Appendix Figure S1

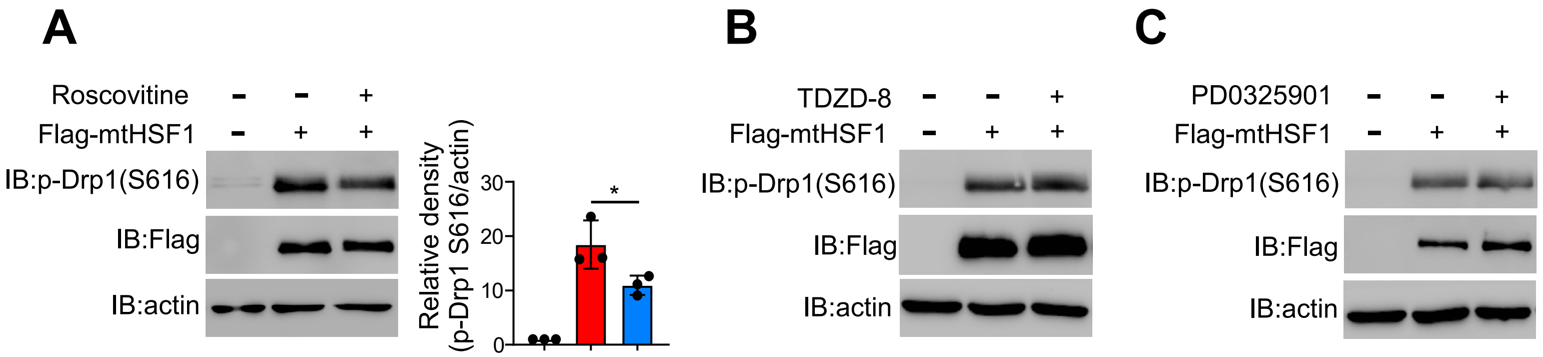

2. Appendix Figure Legand

Appendix Fig. S1:mtHSF1-induced Drp1 phosphorylation at S616 in mitochondria was partly blocked by the CDK inhibitor roscovitine.

A-C: mtHSF1-induced Drp1 phosphorylation at S616 were measured after treatment with roscovitine(20μM, 24h and showed by scatterplot. n=3 biological replicates), TDZD-8(20μM, 24h), PD0325901(800μM, 24h).

The data are the means ± SEMs. One-way ANOVA followed by Tukey's multiple comparison test was used. *P < 0.05.

3. Appendix Table S1

UniProtKB ID	Protein names	Gene names	Molecular function
Q921K2	Poly [ADP-ribose] polymerase	Parp1	DNA-binding transcription activator activity
Q8K1R3	Polyribonucleotide nucleotidyltransferase 1	Pnpt1	polyribonucleotide nucleotidyltransferase activity
O35129	Prohibitin-2	Phb2	Amide binding; sphingolipid binding
P62908	40S ribosomal protein S3	Rps3	Class I DNA endonuclease activity; DNA binding
Q9Z110	Delta-1-pyrroline-5-carboxylate synthase (P5CS)	Aldh18a1	Glutamate 5-kinase activity; glutamate-5-semialdehyde dehydrogenase activity
P10107	Annexin A1 (p35)	Anxa1	Calcium-dependent phospholipid binding; calcium-dependent protein binding
P68040	Receptor of activated protein C kinase 1	Rack1	Cysteine-type endopeptidase activator activity involved in apoptotic process
Q9CYR0	Single-stranded DNA-binding protein (MtSSB)	Ssbp1	Single-stranded DNA binding
Q8BFR5	Elongation factor Tu	Tufm	Translation elongation factor activity; GTPase activity
F6RN86	4-aminobutyrate aminotransferase	Abat	Pyridoxal phosphate binding; transaminase activity
F7D3P8	ATP synthase subunit O	Atp5o	Proton-transporting ATP synthase activity, rotational mechanism
P62897	Cytochrome c	Cycs	Electron transporter
Q0VBL6	Hypoxia-inducible factor 3-alpha	Hif3a	DNA binding; DNA-binding transcription factor activity
Q76I25	HIG1 domain family member 1C	Higd1c	Mitochondrial respirasome assembly
Q8R5K4	Nucleolar protein 6	Nol6	RNA binding
O54724	Caveolae-associated protein 1	Cavin1	rRNA primary transcript binding
Q8R4U6	DNA topoisomerase I	Top1mt	DNA binding; DNA topoisomerase type I activity

4. Appendix Table S2

Antibodies	Antigen	Source	Identifier	Dilution
Anti-HSF1	rabbit	proteintech	51034-1-AP	1:500
Anti-HSF1	rabbit	millipore	ABE1044	1:1000
Anti-HSF1	rabbit	abcam	ab2923	1:1000
Anti-VDAC1	rabbit	abcam	Ab15895	1:2000
Anti-HSP60	mouse	santa cruz	sc-13115	1:1000
Anti-DYKDDDDK Tag(Flag)	mouse	CST	8146S	1:1000
Anti-DYKDDDDK Tag(Flag)	rabbit	CST	14793S	1:1000
Anti-DYKDDDDK Tag(Flag)	rat	Thermo Fisher Scientific	MA1-142-A555	1:100
Anti-Mcl1	rabbit	proteintech	16225-1-AP	1:1000
Anti-TFAM	rabbit	abcam	ab131607	1:1000
Anti-ATAD3A	rabbit	Novus	H00055210-D01P	1:2000
Anti-Tom20	rabbit	abcam	ab78547	1:1000
Anti-Tom20	mouse	santa cruz	sc-136211	1:100
Anti-Huntingtin Protein Antibody, a.a. 181-810, clone 1HU-4C8	mouse	millipore	MAB2166	1:1000
Anti-Polyglutamine-Expansion Diseases Marker Antibody, clone 5TF1-1C2	mouse	millipore	MAB1574	1:1000
Anti-actin	mouse	ZSGB-BIO	TA-09	1:2000
Anti-GFP	mouse	Invitrogen	14-6674-82	1:1000

Anti-GFP	rabbit	Chemicon	ab3080	1:1000
Anti-GFP	chicken	Millipore	ab16901	1:1000
Anti-NeuN	mouse	arigo	arg52283	1:1000
Anti-DARPP32	rabbit	abcam	Ab40801	1:1000
Anti-Caspase3	rabbit	proteintech	19677-1-AP	1:1000
Anti-DNA	mouse	Progen	61014	1:100
Anti-SSBP1	rabbit	proteintech	12212-1-AP	1:1000
Anti-SSBP1	rabbit	santa cruz	sc-67101	1:300
Anti-DRP1	mouse	BD bioscience	611113	1:1000
Anti-COX2	rabbit	proteintech	55070-1-AP	1:1000
Anti-HSF2	rabbit	Abclonal	A12264	1:1000
Anti-HSF4	rabbit	proteintech	18797-1-AP	1:1000
Anti-Clpp	rabbit	abcam	ab124822	1:2000
Anti-PGC1α	rabbit	Novus	NBP104676	1:1000
Anti-Cleaved PARP (Asp214) Antibody	rabbit	CST	9541S	1:1000
Anti-HSP90	rabbit	santa cruz	sc-7947	1:500
Anti-HSP70	rabbit	CST	4872T	1:1000
Anti-cMyc	mouse	santa cruz	sc-40	1:1000
Anti-Matrin3	rabbit	proteintech	12202-2-AP	1:1000
Anti-ATPB	rabbit	proteintech	17247-1-AP	1:1000
Anti-Phospho-DRP1 (Ser616) (D9A1)	rabbit	CST	4494S	1:1000
Phospho-DRP1 (Ser637)	rabbit	CST	4867S	1:1000
Anti-MFN2	rabbit	proteintech	12186-1-AP	1:1000

Anti-OPA1	rabbit	proteintech	27733-1-AP	1:1000
Normal Rabbit IgG	rabbit	CST	2729S	
Mouse (E5Y6Q) mAb IgG2a Isotype Control	mouse	CST	61656S	
Anti-Sox2	goat	R&D	AF2018	1:1000
Anti-CTIP2	rat	abcam	ab18465	1:200
Anti-Ki67	rabbit	ZYMED	180191Z	1:500
Anti-GSH2	rabbit	Millipore	ABN162	1:500
Anti-NANOG	goat	R&D	AF1997	1:1000
Anti-β-III Tubulin	rabbit	Covance	PRB-435P	1:1000
Anti-β-III Tubulin	mouse	Sigma-Aldrich	T8660	1:1000
Anti-FOXG1	rabbit	Abcam	ab18259	1:1000
Anti-MeiS2	goat	Santa Cruz	sc-10599	1:100
Anti-MAP2	mouse	Sigma	M1406	1:1000
Anti-GAD67	mouse	Millipore	MAB5406	1:1000
HRP-linked a-rabbit IgG	rabbit	zen Bioscience	511203	1:5000
HRP-linked a-mouse IgG	mouse	zen Bioscience	511103	1:5000
Hoechst33258		Thermo Fisher Scientific	H1399	1:2000
Alexa 488, goat anti-rabbit Ig G	rabbit	invitrogen	A11034	1:500-1:1000
Alexa 488，donkey anti-mouse Ig G (H+L)	mouse	invitrogen	A21202	1:500-1:1000
Alexa Fluor 488, donkey anti-Rabbit IgG (H+L)	rabbit	Thermo Fisher Scientific	A21206	1:1000

488 Goat anti-chicken	chicken	Jackson	103-545-155	1:1000
Alexa 546，donkey anti-rabbit Ig G (H+L)	rabbit	invitrogen	A10040	1:500-1:1000
Alexa Fluor 546, goat anti-Rat IgG (H+L)	rat	Thermo Fisher Scientific	A11081	1:1000
Alexa Fluor 546, donkey anti-Mouse IgG	mouse	Thermo Fisher Scientific	A10036	1:1000
Alexa Fluor 546, donkey anti-Goat IgG (H+L)	goat	Thermo Fisher Scientific	A11056	1:1000
Alexa 555，goat anti-mouse Ig G (H+L)	mouse	invitrogen	A21422	1:500-1:1000
Alexa 647，Donkey anti-Mouse IgG (H+L)	mouse	invitrogen	A-31571	1:500-1:1000
Alexa 647，Donkey anti-Rabbit IgG (H+L)	rabbit	invitrogen	A-31573	1:500-1:1000
Alexa Fluor 647, donkey anti-Goat IgG (H+L)	goat	Thermo Fisher Scientific	A21447	1:1000

5. Appendix Table S3

	**Permeabilization and block**	**Primary antibody**	**Secondary antibody**	**Mount**
HdhQ7 / HdhQ111 cells (Figs 2B, EV5A, and C)	0.1% Triton X-100 (Biolink, TB0198) for 5 min<br><br>0.05% Triton X-100 and 1% BSA (Sigma, A1933-25G) for 1h	0.05% Triton X-100 and 1% BSA overnight at 4°C	0.05% Triton X-100 and 1% BSA for 1h	Fluoromount-G (Southern Biotech, 0100-01)
Primary neurons (Fig 5A)	0.1% Triton X-100 for 5 min<br><br>0.05% Triton X-100 and 1% BSA for 1h	0.05% Triton X-100 and 1% BSA overnight at 4℃	0.05% Triton X-100 and 1% BSA for 30 min	Fluoromount-G
Mice brain sections (Figs 2E, 5E, 5G, and EV5E)	0.3% Triton X-100 for 30 min<br><br>0.3% Triton X-100 and 10% FBS (Gibco, 16140071) for 2-4h	0.3% Triton X-100 and 10% FBS overnight at 4℃	0.3% Triton X-100 and 10% FBS for 1-2h	Fluoromount-G
Organoids (Figs 1F, 1H, 2I, 2J, 3J, 3K, 5C, 7B, 7C, 7D, 7F, EV2A, EV2B, and EV2C)	1% Triton X-100 and 5% Donkey Serum (Millipore, S30) for 1h	0.1% Triton X-100 and 5% Donkey Serum overnight at 4℃	5% Donkey Serum for 1h	Fluoromount-G
Organoid-dissociated neurons (Fig 7E) and iPSCs (Fig EV1D)	0.2% Triton X-100 for 10min<br><br>10% Donkey Serum for 1h	0.1% Triton X-100 and 5% Donkey Serum overnight at 4℃	5% Donkey Serum for 30 min	Fluoromount-G
