# Supplementary material for: Mitochondrial HSF1 triggers mitochondrial dysfunction and neurodegeneration in Huntington's disease
Source: EMBO Mol Med. 2022 Jun 7;14(7):e15851. doi: 10.15252/emmm.202215851 (PMC9260212; doi:10.15252/emmm.202215851)
Supplement: Supplementary file 2 — Expanded View Figures PDF [file EMMM-14-e15851-s008.pdf]

Expanded View Figures

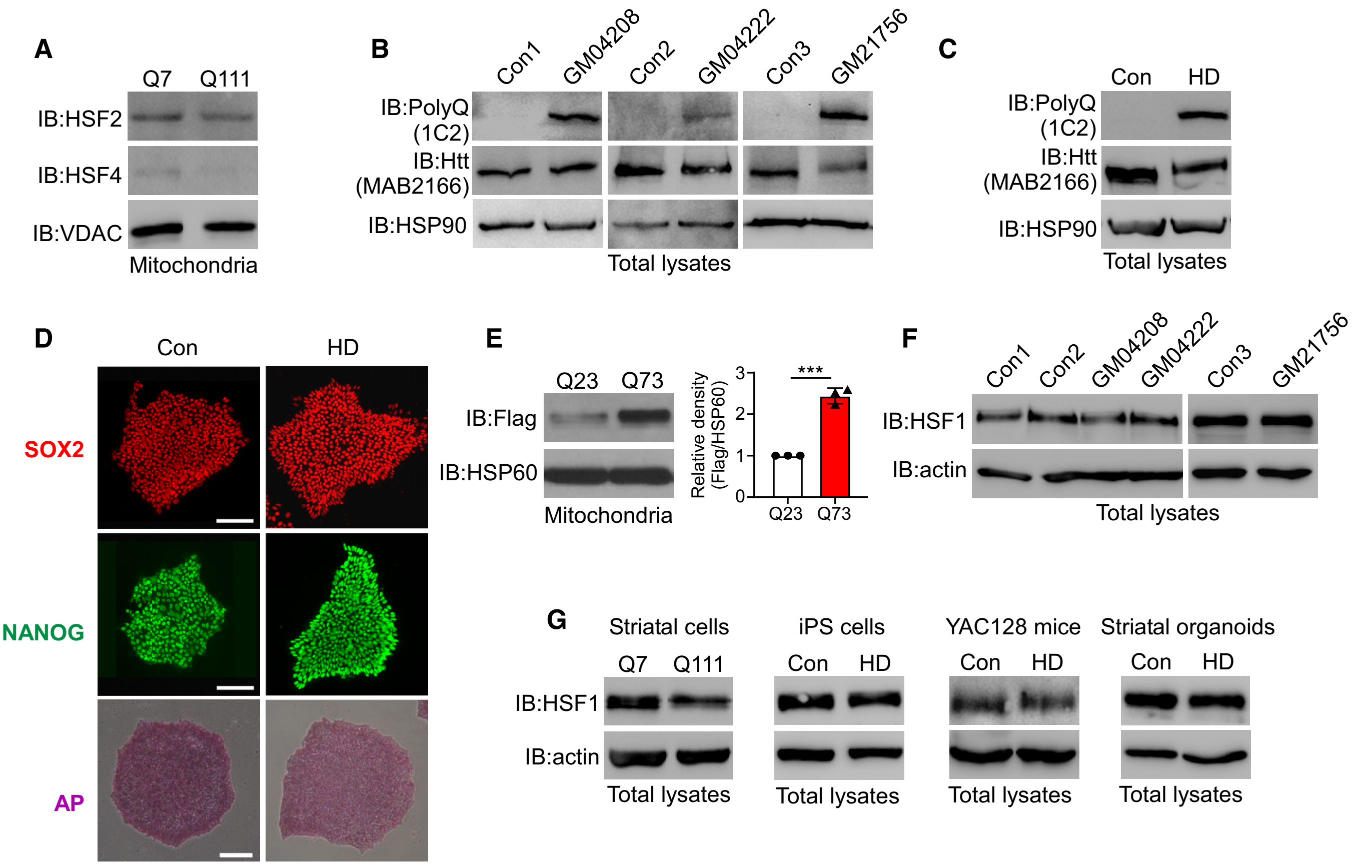

Figure EV1. HSF1 associates with mitochondria.

A Mitochondria-enriched fractions were isolated from HdhQ7 or HdhQ111 cells. HSF2 and HSF4 protein levels in the mitochondria were detected by WB analysis (n = 3 biological replicates).

B, C Total lysates were harvested from control iPSCs, HD iPSCs, control fibroblasts, and HD patient fibroblasts. Immunoblot analysis of htt was performed with 1C2 and MAB2166 (n = 3 biological replicates).

D Characterization of hiPSCs by immunostaining for the pluripotency markers SOX2 and NANOG and staining for alkaline phosphatase (AP) (n = 2 biological replicates). The scale bar represents 100 μm.

E Flag-HSF1 was transfected with Myc-Q23 or Myc-Q73 into HEK293 cells. Mitochondria-localized HSF1 was examined by WB analysis (n = 3 biological replicates). The data are the means ± SEMs; unpaired Student's t-test was used. ***$P < 0.001$.

F Total HSF1 levels in fibroblasts were measured by WB analysis (n = 2 biological replicates).

G Total HSF1 protein levels were tested in the indicated samples by immunoblotting (n = 3 biological replicates).

Source data are available online for this figure.

 © 2022 The Authors

Figure EV2. Construction of mtHSF1 and the procedure for human striatal organoid differentiation.

A Schematic of the protocol for differentiating human striatum-like organoids from hPSCs and immunostaining for the LGE progenitor marker GSH2 and the MSN marker DARPP32 at D30, D45, and D60. The scale bar represents 100 μm.
B Identification of telencephalon and striatum markers by immunostaining. The scale bar represents 100 μm.
C Confirmation of maturation of striatal organoids by robust MAP2 staining and sparse GSH2 and KI67 staining. The scale bar represents 100 μm.
D UMAP visualization of single-cell RNA expression in striatal organoids (n = 11,127 cells).
E Cell-type compositions of human striatal organoids at D30 and D60.
F UMAP plots showing the gene expression patterns of representative marker genes for each cell type. The relative expression level is indicated by the color from gray to red.
G Correlation with BrainSpan dataset of the developing human brain (PCW 8–19).
H Volcano and violin plots for cell type-specific genes differentially expressed in neuronal (left) cells and neural progenitor cells (right) from striatal organoids at D30 and D60.
I Mitochondria-enriched fractions isolated from HdhQ111 cells were exposed to proteinase K for 20 min at 3 μg/ml. Protein levels were detected with the indicated antibodies.
J The htt gene expression was measured by real-time PCR (n = 3 biological replicates).

Data information: The data are the means ± SEMs; unpaired Student's t-test was used. *$P < 0.05$ and ****$P < 0.0001$.
Source data are available online for this figure.

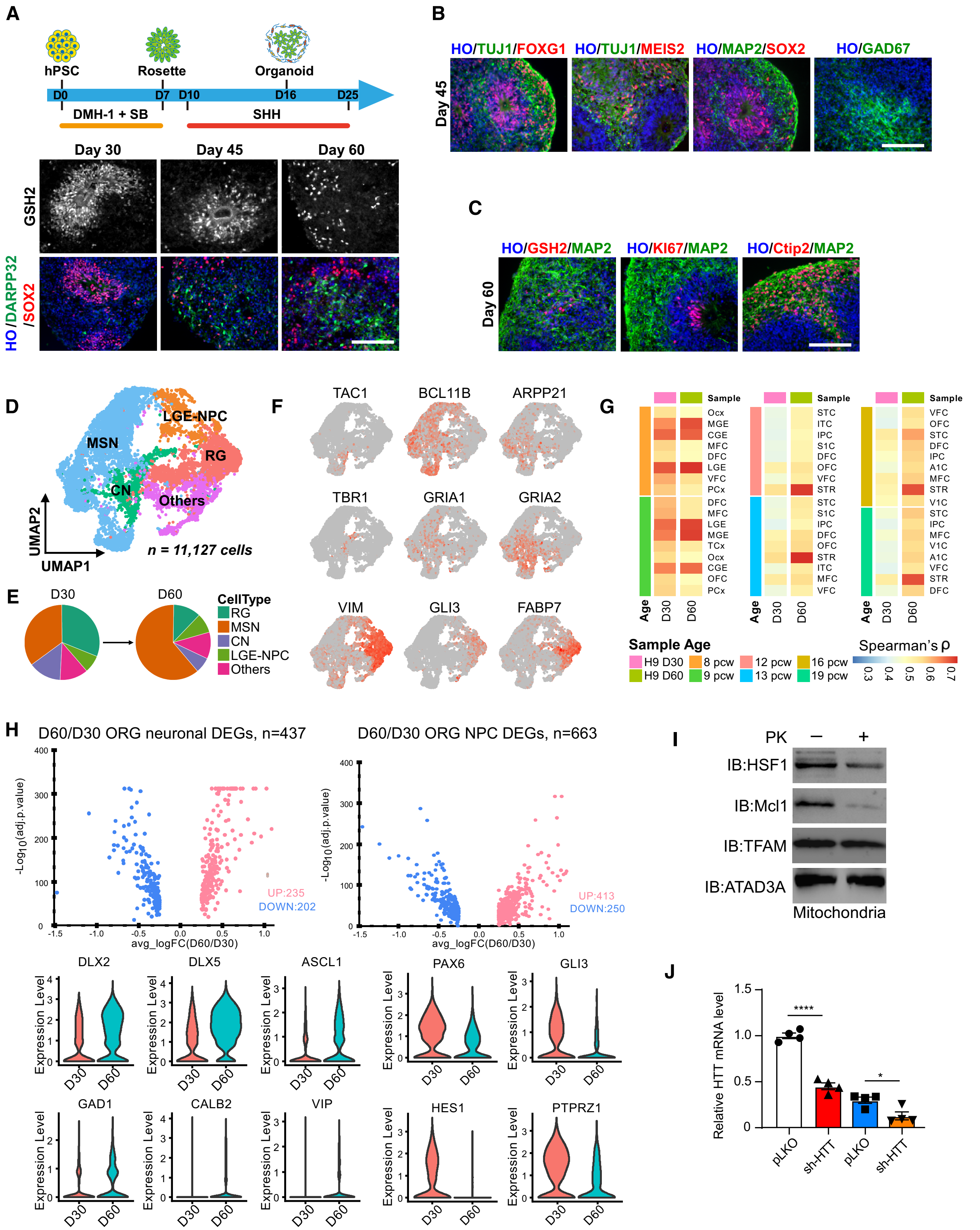

Figure EV2.

 © 2022 The Authors

Figure EV3. HSF1-induced mitochondrial dysfunction is mediated by Drp1 and SSBP1.

A An empty vector or Flag-mtHSF1 was transfected into HEK293 cells. Isolated mitochondria-enriched fractions were analyzed by WB analysis ($n = 2$ biological replicates).

B Flag-mtHSF1 was transfected into HdhQ7 cells. Drp1 protein levels were determined in the mitochondria-enriched fractions by immunoblotting ($n = 3$ biological replicates).

C Myc-Q23 or Myc-Q73 was transfected into HdhQ7 cells. The p-Drp1 S616 levels was examined by WB analysis ($n = 3$ biological replicates). The data are the means ± SEMs; unpaired Student's *t*-test was used. ****P* < 0.001.

D, E The protein levels of p-Drp1(S616) were determined in mitochondria-enriched fractions or cytosolic fractions by WB analysis ($n = 3$ biological replicates).

F AAV-Con or AAV-mtHSF1 was injected into WT mice. The sublocalization of mtHSF1 was determined with anti-HSF1 or anti-Flag antibodies ($n = 2$ biological replicates).

G SSBP1 protein levels in HdhQ7 or HdhQ111 cells were determined by WB analysis ($n = 3$ biological replicates).

H Flag-mtHSF1 was transfected into HdhQ7 cells. The total levels of SSBP1 were tested by immunoblotting ($n = 3$ biological replicates).

I Patient fibroblasts and control fibroblasts were harvested and subjected to immunoprecipitation with an anti-HSF1 antibody. The immunoprecipitates were analyzed by WB analysis ($n = 2$ biological replicates).

J Left: total protein from WT or Drp1-KO MEFs was immunoblotted with an anti-HSF1 antibody. Right: nuclear fractions of WT or Drp1-KO MEFs were collected and analyzed by immunoblotting ($n = 2$ biological replicates).

K Flag-HSF1 was transfected with Myc-Drp1 or an empty vector into HEK293 cells. HSF1 was analyzed in the mitochondria-enriched fractions by immunoblotting ($n = 3$ biological replicates).

Data information: The data are the means ± SEMs; unpaired Student's *t*-test was used. ***P* < 0.01.
Source data are available online for this figure.

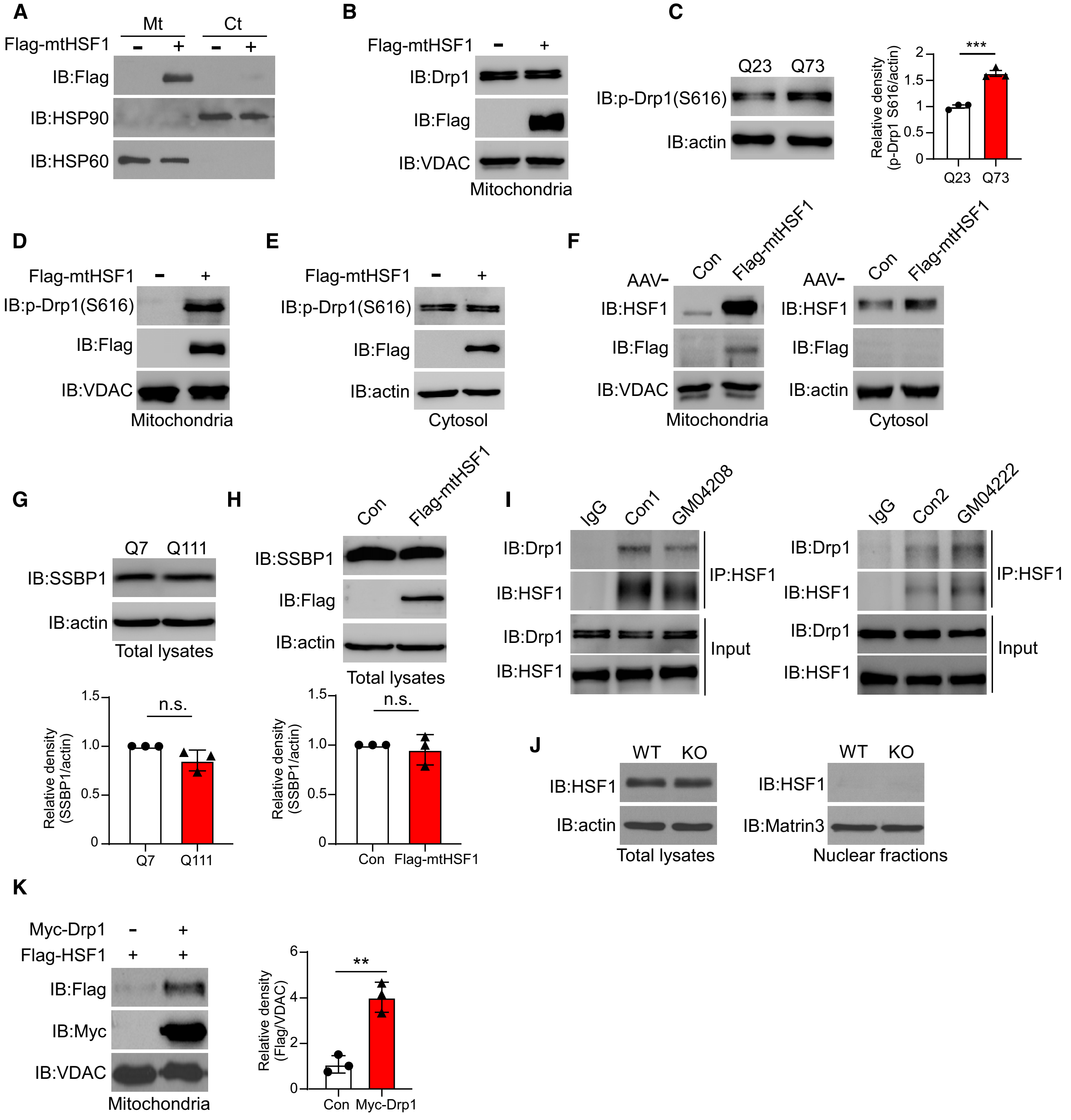
A
Mt
Ct
Flag-mtHSF1
IB:Flag
IB:HSP90
IB:HSP60
B
Flag-mtHSF1
IB:Drp1
IB:Flag
IB:VDAC
Mitochondria
C
Q23 Q73
IB:p-Drp1(S616)
IB:actin
Relative density (p-Drp1 S616/actin)
***
D
Flag-mtHSF1
IB:p-Drp1(S616)
IB:Flag
IB:VDAC
Mitochondria
E
Flag-mtHSF1
IB:p-Drp1(S616)
IB:Flag
IB:actin
Cytosol
F
AAV-
Con
Flag-mtHSF1
IB:HSF1
IB:Flag
IB:VDAC
Mitochondria
IB:actin
Cytosol
G
Q7 Q111
IB:SSBP1
IB:actin
Total lysates
n.s.
Relative density (SSBP1/actin)
H
Con
Flag-mtHSF1
IB:SSBP1
IB:Flag
IB:actin
Total lysates
I
IgG
Con1
GM04208
Con2
GM04222
IB:Drp1
IB:HSF1
IP:HSF1
Input
J
WT KO
IB:HSF1
IB:actin
Total lysates
IB:Matrin3
Nuclear fractions
K
Myc-Drp1
Flag-HSF1
IB:Flag
IB:Myc
IB:VDAC
Mitochondria
**
Relative density (Flag/VDAC)
Con Myc-Drp1

Figure EV3.

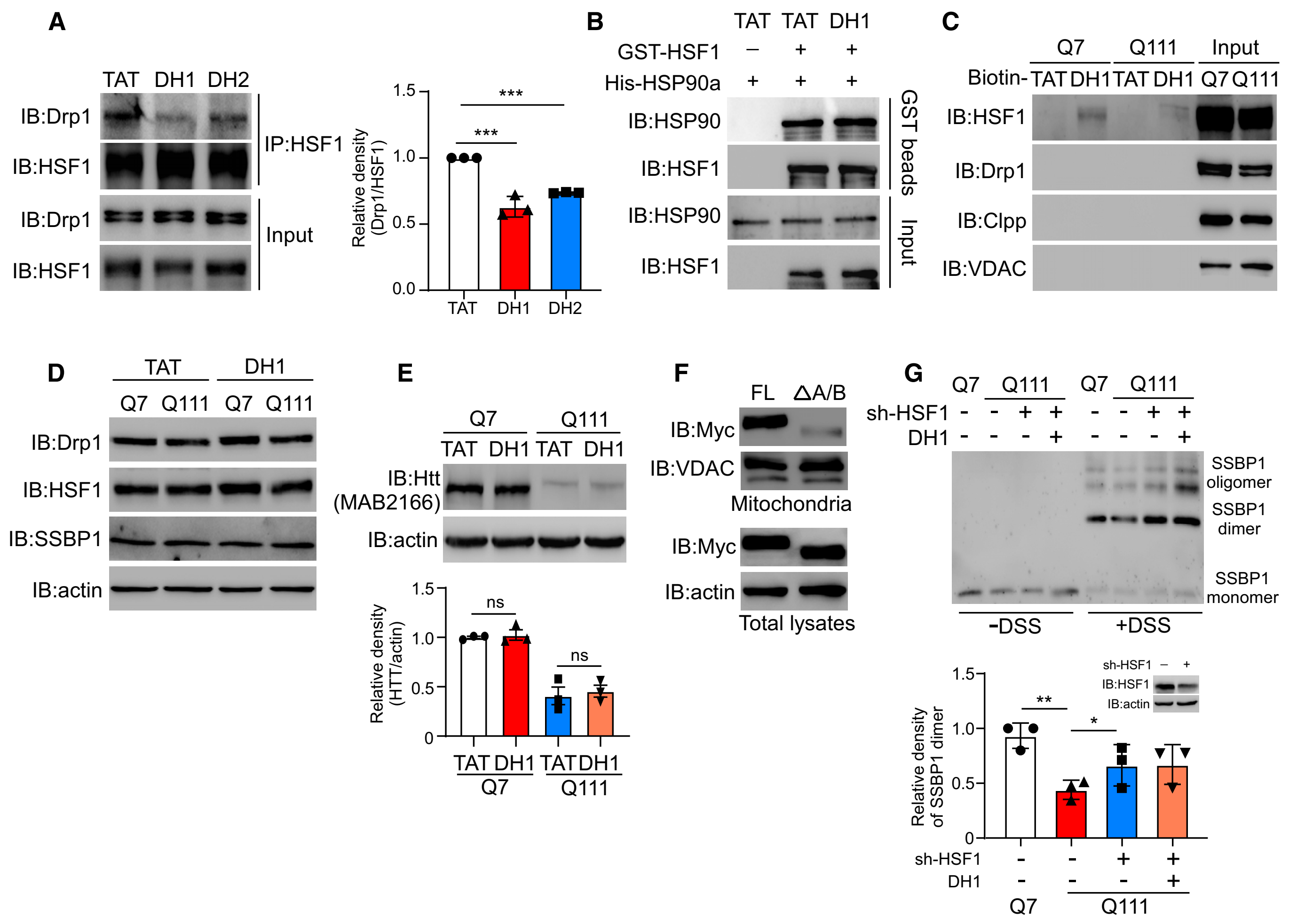

Figure EV4. DH1 reduces binding of Drp1 and HSF1.

A HdhQ111 cells were treated with TAT, DH1, or DH2 (1 μM, 3 days). Total proteins were extracted and subjected to immunoprecipitation with an anti-HSF1 antibody. The immunoprecipitates were analyzed by WB analysis (n = 3 biological replicates). The data are the means ± SEMs; one-way ANOVA followed by Tukey's multiple comparison test was used. ***$P < 0.001$.

B The recombinant protein GST-HSF1 was incubated with DH1 or TAT and then mixed with His-HSP90a. The immunoprecipitates were analyzed by WB analysis (n = 2 biological replicates).

C Cell extracts were collected from HdhQ7 or HdhQ111 cells, treated with biotin-labeled TAT or DH1 (10 μM), and incubated with streptavidin beads. The immunoprecipitates were analyzed by WB analysis (n = 2 biological replicates).

D, E HdhQ7 or HdhQ111 cells were treated with TAT or DH1 (1 μM, 3 days). Immunoblot analysis was performed to examine the total levels of Drp1, HSF1, HTT, and SSBP1 (n = 3 biological replicates).

F Flag-HSF1 or Flag-HSF1ΔA/B was transfected into HdhQ7 cells. Isolated mitochondria-enriched fractions were analyzed by immunoblotting (n = 3 biological replicates).

G HSF1-knockdown cells or control cells were treated with DH1 or TAT (1 μM, 3 days). SSBP1 oligomers were examined by immunoblotting (n = 3 biological replicates).

Data information: The data are the means ± SEMs; one-way ANOVA followed by Dunnett's multiple comparisons test was used for (A); Tukey's multiple comparison test was used for (E and G). *$P < 0.05$, **$P < 0.01$, and ***$P < 0.001$.
Source data are available online for this figure.

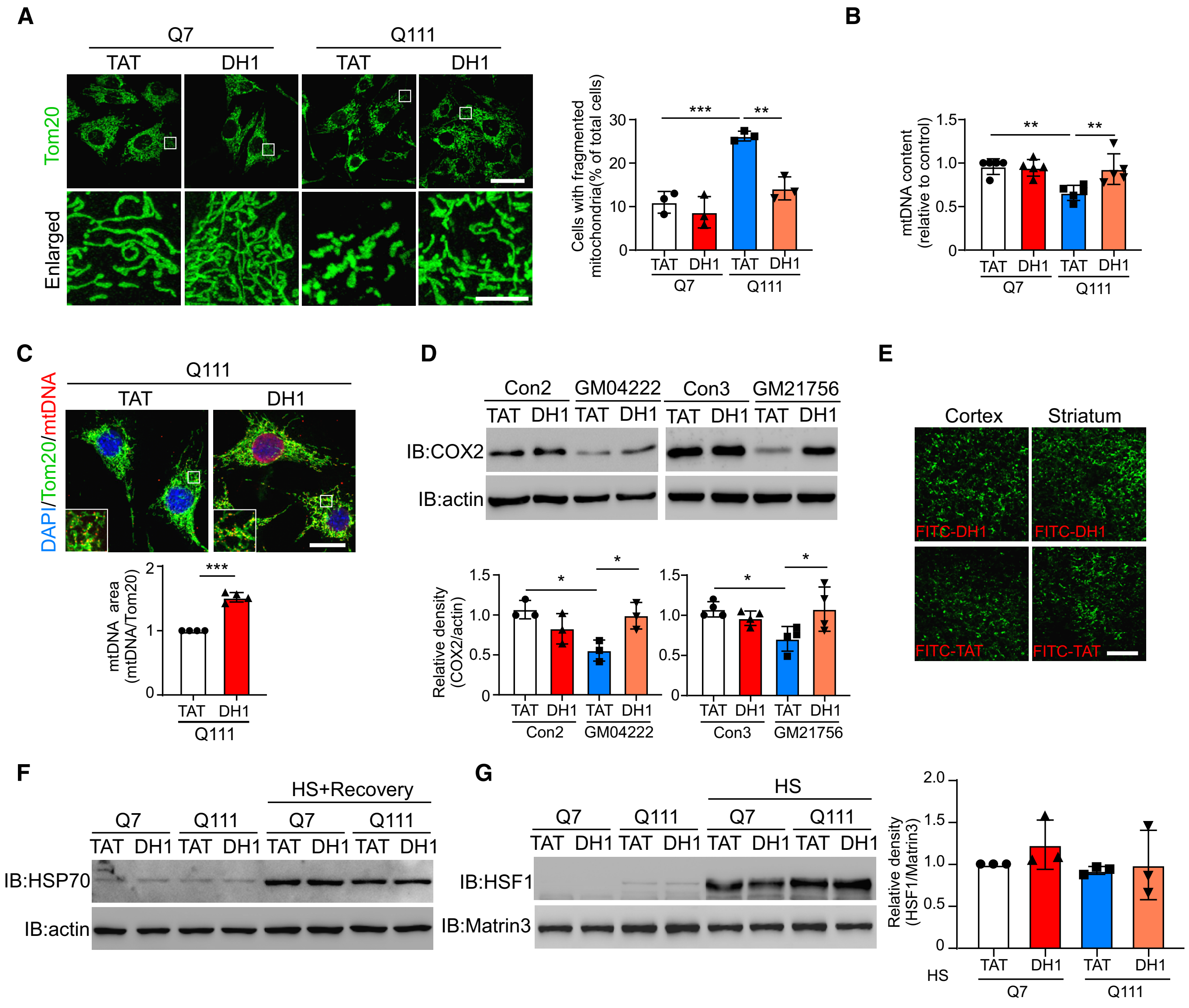

Figure EV5. DH1 improves mitochondrial function.

A HdhQ7 or HdhQ111 cells were treated with TAT or DH1 for 3 days (1 μM). The cells were stained with an anti-TOM20 antibody. Mitochondrial morphology was observed by microscopy. Scatterplot with bar shows the percentage of cells with fragmented mitochondria ($n = 3$ biological replicates). The scale bar represents 40 μm, and the enlarged image scale bar is 4 μm.

B, C HdhQ7 or HdhQ111 cells were treated with TAT or DH1 for 5 days (1 μM). mtDNA content was measured by qPCR ($n = 5$ biological replicates) or immunostaining with an anti-DNA antibody ($n = 4$ biological replicates) and is shown by scatterplot. The scale bar represents 20 μm.

D Patient fibroblasts and control fibroblasts were treated with TAT or DH1 for 3 days (1 μM). COX2 protein levels were tested by WB analysis (n = 3–4 biological replicates).

E FITC-conjugated DH1 or TAT was injected intraperitoneally into WT mice (3 mg/kg, each) for 5 days. Brain sections were observed by microscopy. The scale bar is 100 μm.

F HdhQ7 or HdhQ111 cells were treated with TAT or DH1 for 3 days (1 μM). The cells were cultured at 42°C for 1 h and then allowed to recover for 3 h. HSP70 levels was examined by immunoblotting ($n = 2$ biological replicates).

G TAT- or DH1-treated cells were cultured at 42°C for 1 h. Nuclear fractions were isolated and measured by WB analysis ($n = 3$ biological replicates).

Data information: The data are the means ± SEMs from at least three independent biological experiments; one-way ANOVA followed by Tukey's multiple comparison test was used. *$P < 0.05$, **$P < 0.01$, and ***$P < 0.001$.
Source data are available online for this figure.

 © 2022 The Authors
