# Supplementary figures and images for: Mitochondrial HSF1 triggers mitochondrial dysfunction and neurodegeneration in Huntington's disease
Source: EMBO Mol Med. 2022 Jun 7;14(7):e15851. doi: 10.15252/emmm.202215851 (PMC9260212; doi:10.15252/emmm.202215851)

Appendix Figure S1A

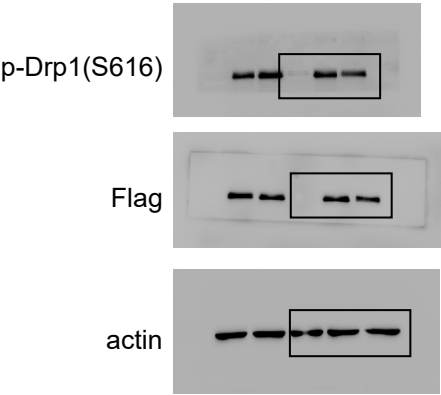

Appendix Figure S1B

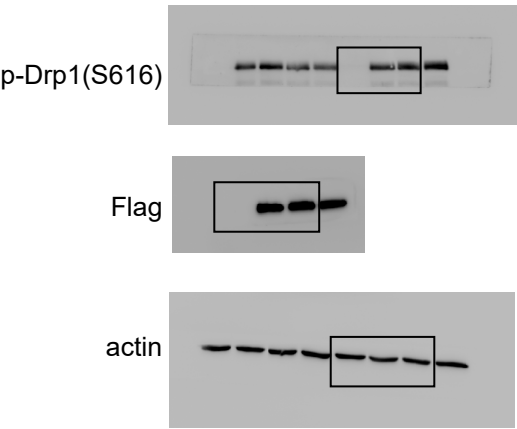

Appendix Figure S1C

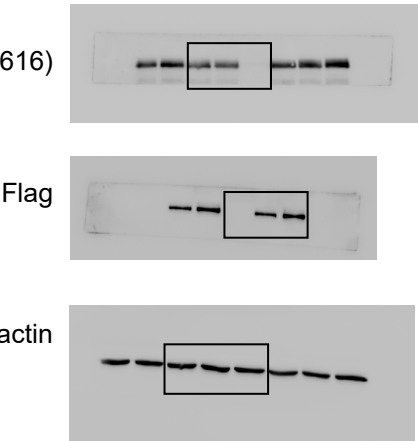

Supplement: Supplementary file 3 — Source Data for Expanded View and Appendix [file EMMM-14-e15851-s002.zip › Source Data For Appendix Figure S1.pdf]

Figure 1A

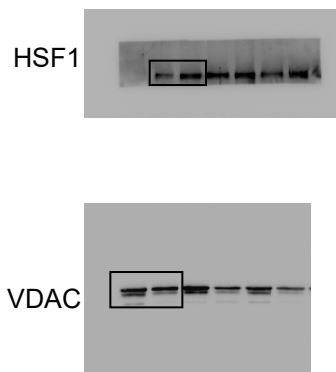

Figure 1B

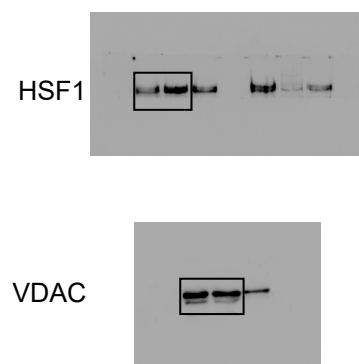

Figure 1C

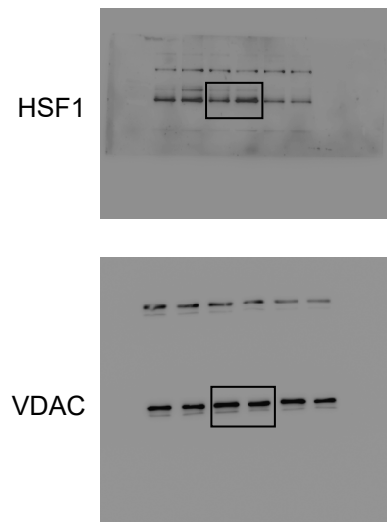

Figure 1D

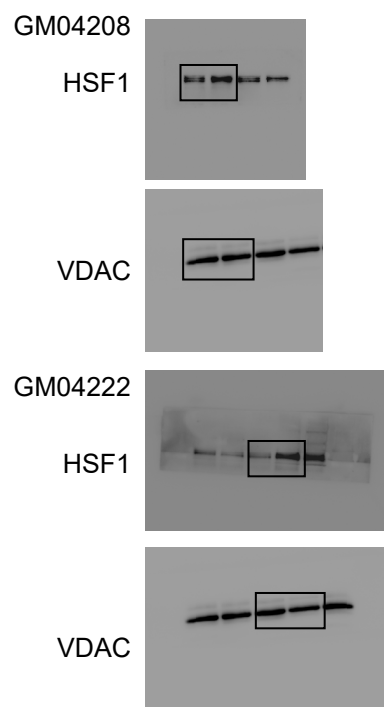

GM21756

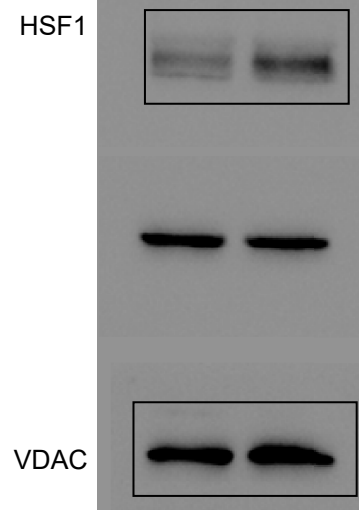

Figure 1E

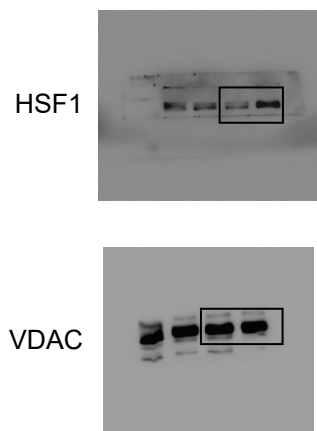

Figure 1J

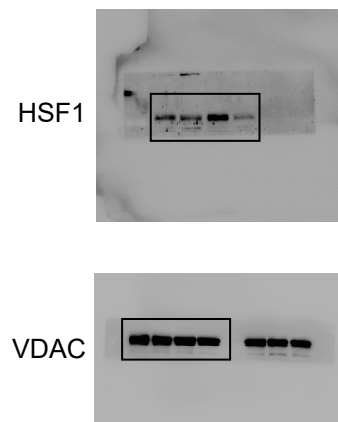

HTT

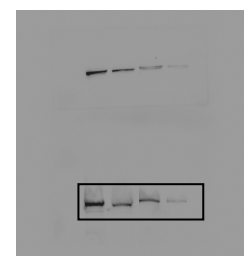

actin

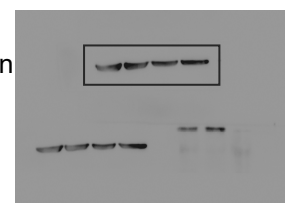

Supplement: Supplementary file 4 — Source Data for Figure 1 [file EMMM-14-e15851-s005.pdf]

Figure 2C

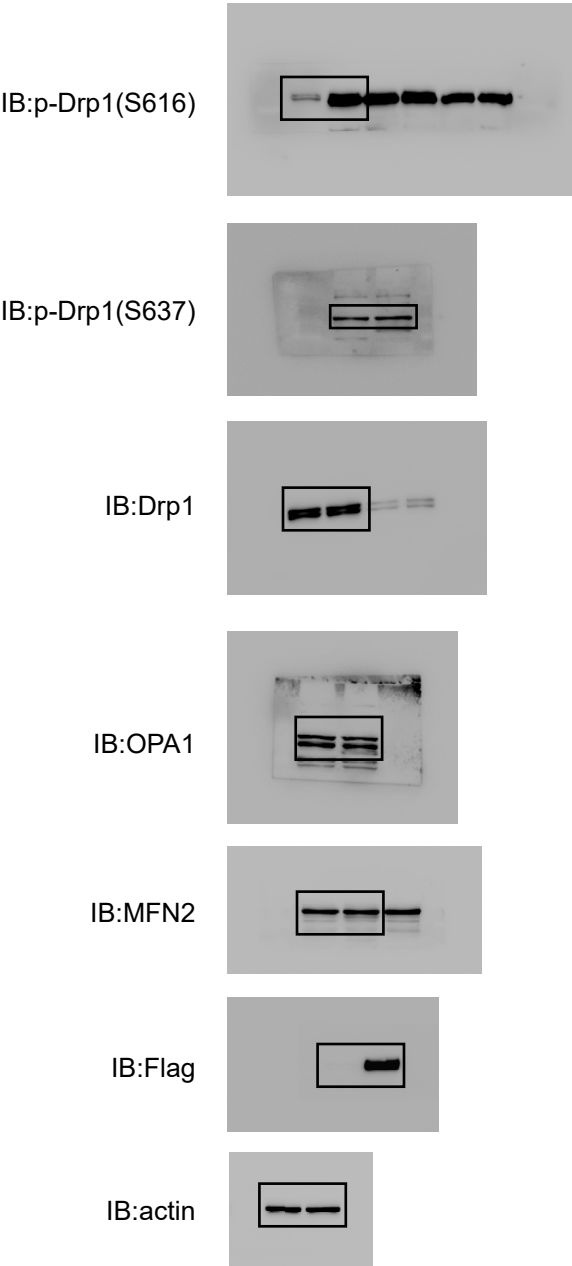

Figure 2F

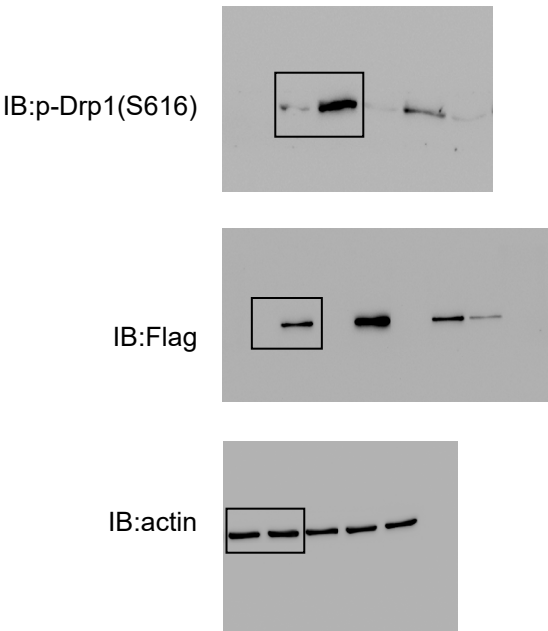

Supplement: Supplementary file 5 — Source Data for Figure 2 [file EMMM-14-e15851-s009.pdf]

Figure 3E

c-PARP

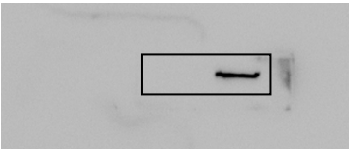

HSF1

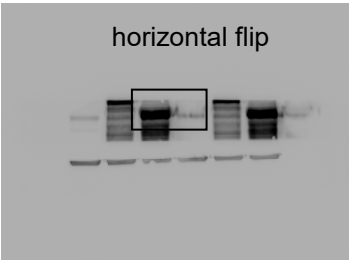

actin

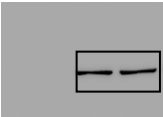

Figure 3H

Caspase3

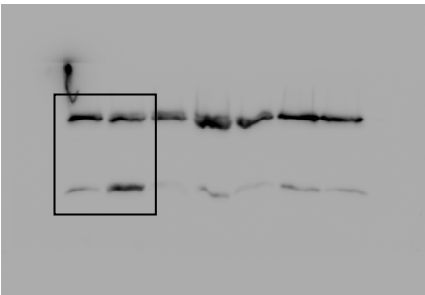

actin

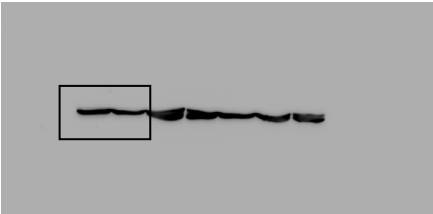

Supplement: Supplementary file 6 — Source Data for Figure 3 [file EMMM-14-e15851-s003.pdf]

Figure 4B

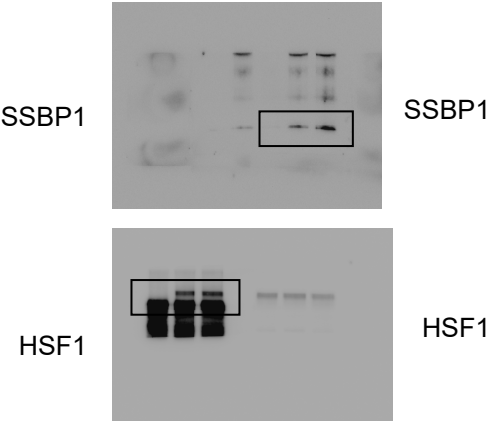

Figure 4C

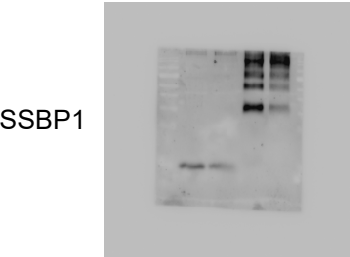

Figure 4D

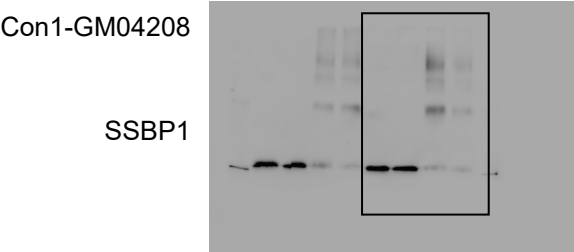

Figure 4E

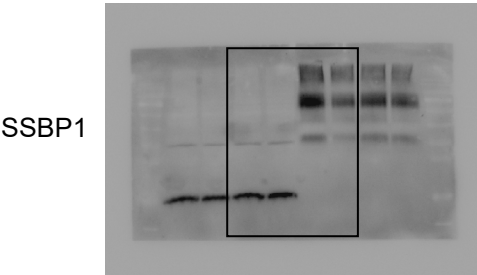

Con2-GM04222

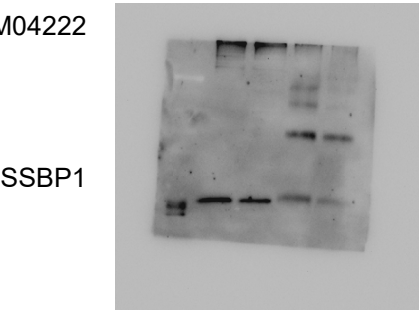

Figure 4F

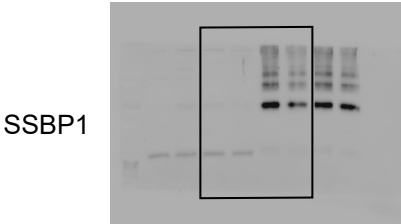

Figure 4H

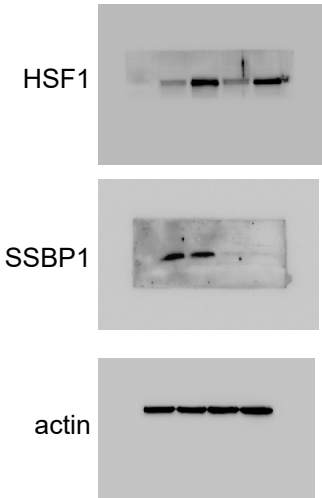

Figure 4G

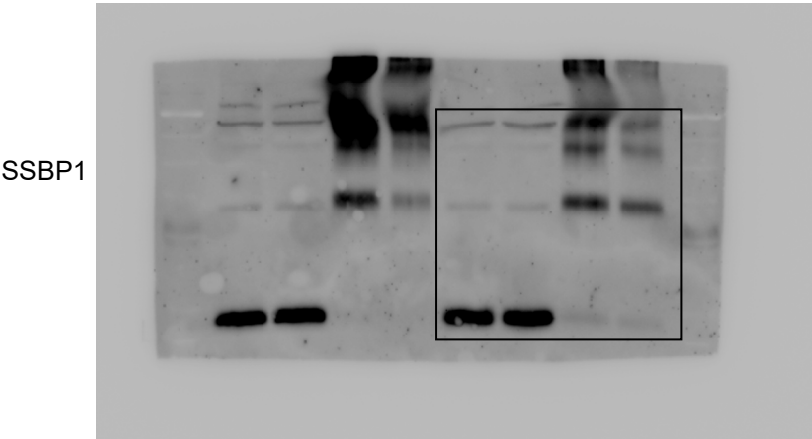

Supplement: Supplementary file 7 — Source Data for Figure 4 [file EMMM-14-e15851-s011.pdf]

Figure 5F

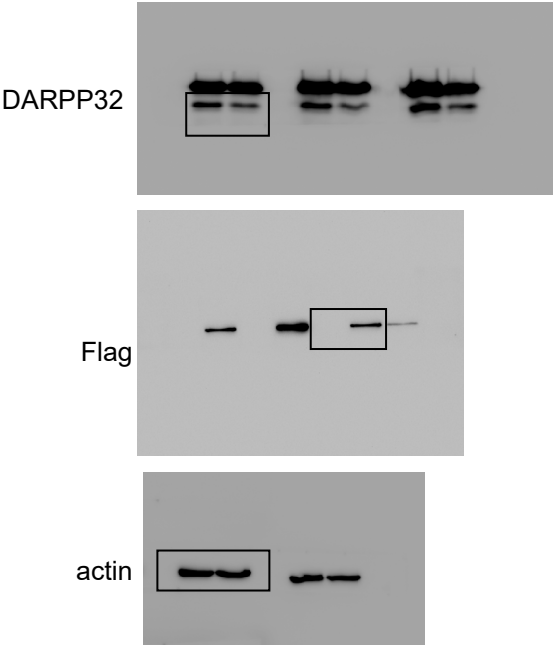

Supplement: Supplementary file 8 — Source Data for Figure 5 [file EMMM-14-e15851-s006.pdf]

Figure 6A

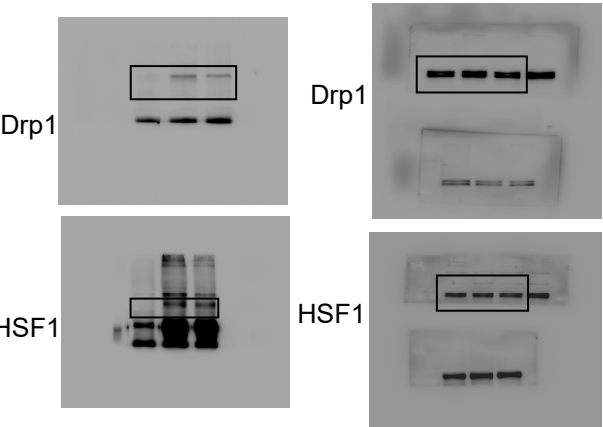

Figure 6B

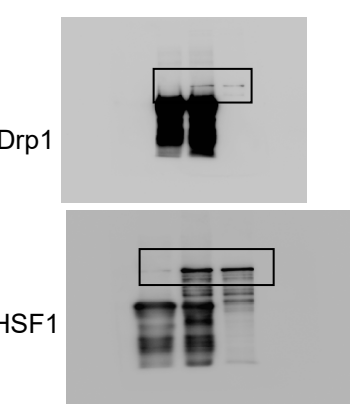

Figure 6C

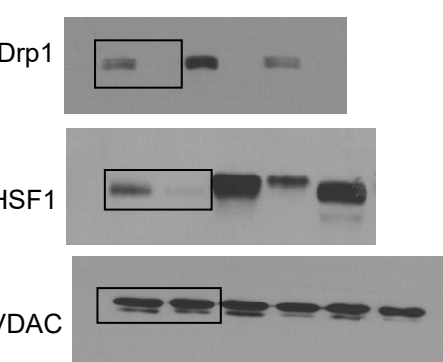

Figure 6F

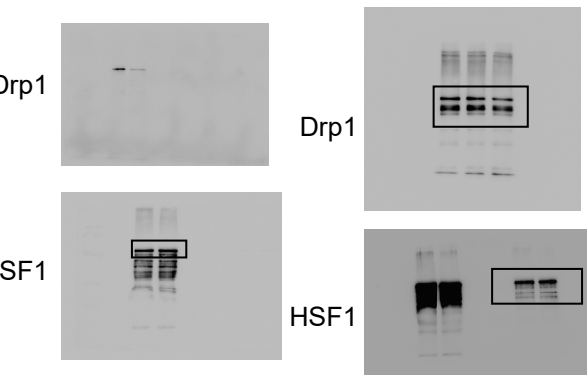

Figure 6G

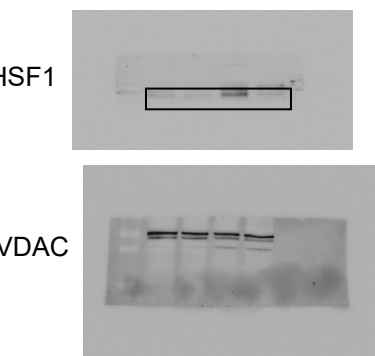

Figure 6H

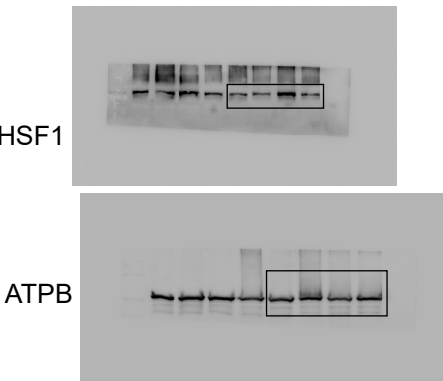

Figure 6I

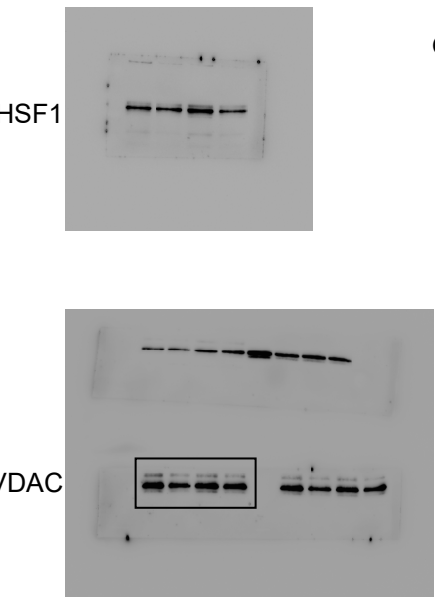

Figure 6J

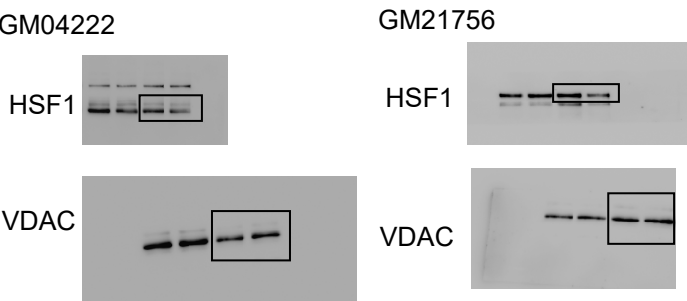

Figure 6K

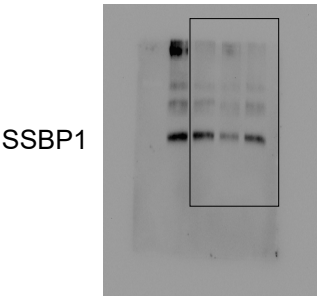

Figure 6L

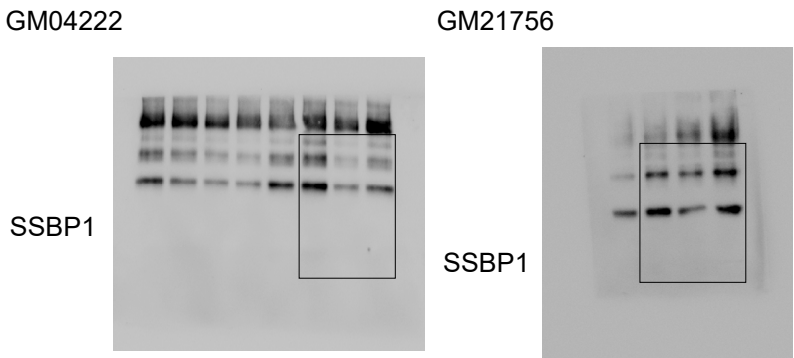

Supplement: Supplementary file 9 — Source Data for Figure 6 [file EMMM-14-e15851-s001.pdf]

Figure 7H

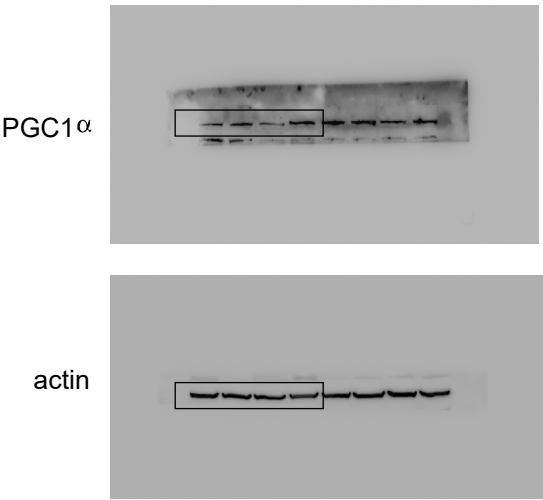

Figure 7I

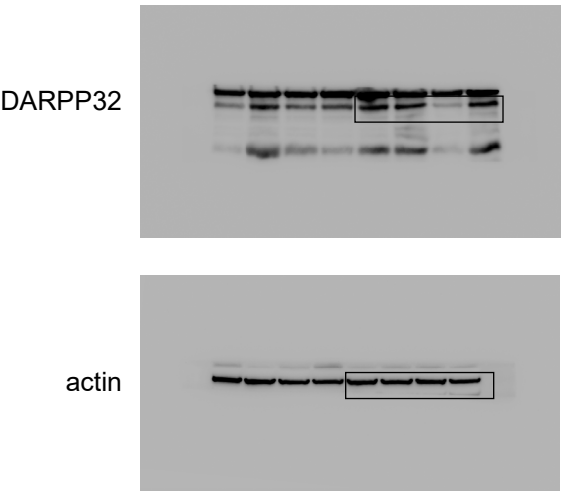

Supplement: Supplementary file 10 — Source Data for Figure 7 [file EMMM-14-e15851-s007.pdf]
