# Supplementary material for: Mitochondrial HSF1 triggers mitochondrial dysfunction and neurodegeneration in Huntington's disease
Source: EMBO Mol Med. 2022 Jun 7;14(7):e15851. doi: 10.15252/emmm.202215851 (PMC9260212; doi:10.15252/emmm.202215851)
Supplement: Supplementary file 11 [file EMMM-14-e15851-s004.pdf]

EMBO Molecular Medicine

Mitochondrial HSF1 triggers mitochondrial dysfunction and neurodegeneration in Huntington's disease

Chunyue Liu, Zixing Fu, Shanshan Wu, Xiaosong Wang, Shengrong Zhang, Chu Chu, Yuan Hong, Wenbo Wu, Shengqi Chen, Yueqing Jiang, Yang Wu, Yongbo Song, Yan Liu, and Xing Guo
DOI: 10.15252/emmm.202215851

Corresponding author(s): Xing Guo (guox@njmu.edu.cn) , Yan Liu (yanliu@njmu.edu.cn)

Review Timeline:

Submission Date:	8th Feb 22
Editorial Decision:	10th Mar 22
Revision Received:	13th Apr 22
Editorial Decision:	3rd May 22
Revision Received:	10th May 22
Accepted:	10th May 22

Editor: Jingyi Hou

Transaction Report:

(Note: With the exception of the correction of typographical or spelling errors that could be a source of ambiguity, letters and reports are not edited. Depending on transfer agreements, referee reports obtained elsewhere may or may not be included in this compilation. Referee reports are anonymous unless the Referee chooses to sign their reports.)

Thank you again for submitting your work to EMBO Molecular Medicine. We have now heard back from the three referees who agreed to evaluate your manuscript. As you will see from the reports below, the referees are overall supportive and think the study is interesting. Still, they raise a series of concerns, which we would ask you to address in a revision of the manuscript.

The referees' recommendations are relatively straightforward, so there is no need to reiterate their comments. All issues raised by the referees need to be satisfactorily addressed. Please feel free to contact me in case you would like to discuss in further detail any of the issues raised by the referees.

We would welcome the submission of a revised version within three months for further consideration. Please note that EMBO Molecular Medicine strongly supports a single round of revision. As acceptance or rejection of the manuscript will depend on another round of review, your responses should be as complete as possible.

EMBO Molecular Medicine has a "scooping protection" policy, whereby similar findings that are published by others during review or revision are not a criterion for rejection. Should you decide to submit a revised version, I do ask that you get in touch after three months if you have not completed it to update us on the status.

We are aware that many laboratories cannot function at full efficiency during the current COVID-19/SARS-CoV-2 pandemic and have therefore extended our "scooping protection policy" to cover the period required for a full revision to address the experimental issues. Please let me know should you need additional time, and also if you see a paper with related content published elsewhere.

Please read below for important editorial formatting and consult our author's guidelines for proper formatting of your revised article for EMBO Molecular Medicine.

I look forward to receiving your revised manuscript. Use this link to login to the manuscript system and submit your revision:
https://embomolmed.msubmit.net/cgi-bin/main.plex

***** Reviewer's comments *****

Referee #1 (Remarks for Author):

In this manuscript, Liu et.al. report a novel molecular pathway of HD pathogenesis: HSF1 mislocalizes to the mitochondria and disturbs mitochondrial functions via Drp1 and SSBP1. Based on this, the authors also performed the proof-of-concept study to validate a therapeutic strategy of treating HD by inhibiting HSF1 mislocalization using a peptide inhibitor. The overall study is thorough and of high importance. The data in general is also of high quality. I recommend publish this paper after minor revisions. Some detailed suggestions to further improve the manuscript are listed below:

1. The functional involvement of Drp1 and SSBP1 may need further validation if the authors want to claim them as the major mediators of HSF1. Meanwhile, this is probably outside of the scope of this particular study because the major focus is HSF1. The current data already demonstrates them as possible mediators downstream. The authors may consider discussing other possible pathways and/or further functional validation of Drp1 and SSBP1, for example, by point mutagenesis of the phosphorylation site.
2. The description in the methods part needs to be improved. For example, how did the authors measure the length of mitochondria? which microscope did they use? And what is the age of the mice in each experiment?
3. A graphic abstract or a diagram to summarize the molecular mechanism would be helpful to understand the content of this study.
4. Figure 1. The authors should show the HTT proteins in the patients' fibroblasts.
5. It is desirable that the authors confirm the increased HSF1 translocation to mitochondria by immunofluorescence.
6. It is desirable to provide higher-quality images in Fig.1F.
7. The authors should provide quantitative real-time PCR data in Fig.1G.
8. The authors should indicate the number of mitochondria used for statistical quantification in Fig.2G.
9. The author should clarify where the Drp1 phosphorylation occurs? Is it in the cytoplasm or in mitochondria? Is the level of phosphorylated Drp1 increased by expressing the CAG repeats?
10. The DAPI staining is missing in Fig.2E.
11. The authors should show the mtDNA content in mtHSF1 expressed striatal organoids.
12. The authors should confirm that the cells with fragmented nuclei are neurons in Fig.3I and 7F. Immunostaining for Tuj1 or NeuN would be helpful.
13. Please explain the principle used by the authors in designing the inhibition peptide of HSF1-Drp1.

Referee #2 (Remarks for Author):

The paper by Liu et al. explores a new role of HSF1 in the mitochondrial dysfunction and neurodegeneration in HD.
The use of multiple in vitro and in vivo models, in combination with different and complementary techniques, makes this study a complete and outstanding work that uncover how mtHSF1 can contribute to mitochondrial dysfunction in HD.

There are some minor issues to be considered.

Figure 1
1) Immunofluorescence of the endogenous HSF1, in combination with a mitochondria marker (Tom20 or others), should be performed, at least in wt and HD cellular models, to confirm the accumulation of HSF1 in mitochondria as shown by western blot analyses.

2) The authors checked HSF1 levels in total lysate from wt and HD fibroblasts and they found no differences. What about the other models? Although the focus of this study is the distribution of HSF1 in mitochondria, further data indicating altered translocation of this protein in HD models may be required.

3) In figure 1B, the authors used different proteins (HSP60 or VDAC) to normalize mtHSF1 in different samples. Why? Is only a technical issue?

4) Supplementary figure 1D is not clear. In which cells the authors over-expressed muHTT?

5) In the methods, the protocol to purify mitochondria from tissues is missing. Only the protocol from cells is described. Furthermore, did the author perform a quality check of the mitochondria purification?

Figure 2
6) In figure 2F, I suppose that the WB analyses were performed in the striatum of mice. Please, confirm it.

7) In the striatal organoids, what is the % of MSNs? What is the % of DARPP32+NeuN+ cells? Are there differences between control and HD organoids?

Figure 3
8) Figure 3A is not clear to not experts. Additional infos about how DNA content is quantified by real time PCR for specific genes should be included in the legend and in the Method section.

9) In figure 3I, which AAV was used? Did the authors perform a double viral infection with Flag-mtHSF1 and EGFP? The method about how the authors quantified nuclear fragmentation in striatal organoids is not described.

Figure 5
10) The method to quantify neurite length is missing.
11) The method to quantify % Neu+ cells is missing.
12) Please verify the consistency in AAV control between Methods (AAV-EYFP) and the legend in figure 5C (GFP).

General comments:
The first comment is related to the findings described in this work compared to HSF1 data in the literature. HSF1's role is related to heat shock response, which is impaired in HD mouse models. However, HSF1 level is not altered in a consistent way in all HD models. For example, recently, the basal or induced levels of HSF1 in the brain of zQ175 mice is similar to Wt mice (Gomez-Parades et al., Scientific Reports 2021). A comment about this issue should be included in the discussion.

The second comment is the relationship between HSF1 and cancer. HSF1 also promotes cancer development and progression. How the findings found in HD models are related (or not) with HSF1's role in cancers?

Uncropped blots should be shown in Supplementary material.

In the legends, several infos are missing, such as the type of graph, the number of biological or technical replicates..

Referee #3 (Comments on Novelty/Model System for Author):

In the current manuscript, authors studied the impact of heat shock transcription factor 1 (HSF1) in relation to mitochondria. They tested their concept using HD cell cultures, YAC129 mice, human striatal organoids derived from iPSc. Interestingly they found HSF1 in the mitochondria. They provided compelling evidence to support their conclusion. Methods are acceptable, overall, it is a well done study. Concerns,
1. authors missed citing seminal articles on mitochondrial dysfunction, mitochondrial dynamics and biogenesis done by Shirendeb et al 2011 HMG and Shirendeb et al 2012 and most recently review article by Sawant et al 2021
2. It is unclear, if DH1 reduced full-length mutant huntingtin, please show the data, id experiments/blots are done.

Referee #3 (Remarks for Author):

In the current manuscript, authors studied the impact of heat shock transcription factor 1 (HSF1) in relation to mitochondria. They tested their concept using HD cell cultures, YAC129 mice, human striatal organoids derived from iPSc. Interestingly they found HSF1 in the mitochondria. They provided compelling evidence to support their conclusion. Methods are acceptable, overall, it is a well done study. Concerns,

1. authors missed citing seminal articles on mitochondrial dysfunction, mitochondrial dynamics and biogenesis done by Shirendeb et al 2011 HMG and Shirendeb et al 2012 and most recently review article by Sawant et al 2021
2. It is unclear, if DH1 reduced full-length mutant huntingtin, please show the data, id experiments/blots are done.

Response letter

RE: Mitochondrial HSF1 triggers mitochondrial dysfunction and neurodegeneration in Huntington's disease (manuscript reference EMM-2022-15851-T)

Response to the comments from reviewers

REFEREE #1:

In this manuscript, Liu et.al. report a novel molecular pathway of HD pathogenesis: HSF1 mislocalizes to the mitochondria and disturbs mitochondrial functions via Drp1 and SSBP1. Based on this, the authors also performed the proof-of-concept study to validate a therapeutic strategy of treating HD by inhibiting HSF1 mislocalization using a peptide inhibitor. The overall study is thorough and of high importance. The data in general is also of high quality. I recommend publish this paper after minor revisions. Some detailed suggestions to further improve the manuscript are listed below:

Response: We appreciate the reviewer's positive comment on the value of the study.

Comment 1: 1. The functional involvement of Drp1 and SSBP1 may need further validation if the authors want to claim them as the major mediators of HSF1. Meanwhile, this is probably outside of the scope of this particular study because the major focus is HSF1. The current data already demonstrates them as possible mediators downstream. The authors may consider discussing other possible pathways and/or further functional validation of Drp1 and SSBP1, for example, by point mutagenesis of the phosphorylation site.

Response: We thank the reviewer for this comment. Following the reviewer's suggestion, the following sentences were added in discussion in the revised manuscript.

Discussion- page16

Our results suggest that HSF1 interacts with SSBP1 on mitochondria and impairs SSBP1 oligomerization, which in turn reduces mtDNA content. The lysine at amino-acid 188 (K188) of HSF1 is required for the interaction between HSF1 and SSBP1. It will be of interest to investigate whether the mtHSF1-K188R could abolish the effect of HSF1 on mtDNA replication. As an interactor of HSF1, P53 directly regulates COX I in a manner dependent on its transcriptional activity (Qi et al, 2011). In addition, our proteomics data reveals that mtHSF1 may interact with DNA binding proteins such as Top1mt, Hif3a, Rps3, and Parp1. Thus, the role of HSF1 in controlling the mtDNA expression in HD should be more carefully investigated in future studies.

Comment 2: The description in the methods part needs to be improved. For example, how did the authors measure the length of mitochondria? which microscope did they use? And what is the age of the mice in each experiment?

Response: Thanks for the helpful suggestions. We implanted a 42-day osmotic pumps (Alzet, Cupertino CA,2006) containing the TAT control peptide or the DH1 peptide into Hemizygous YAC128 male mice (Tg) and their age-matched WT littermates at 6 months

(Please see methods section). AAV-Con and AAV-mtHSF1 were injected into the striata of WT mice at 8 weeks of age. Mitochondrial morphology and neural function were analyzed after 3 weeks **(Please see figure legend section, Fig 2D).** In this revision, we added more details about measurement of mitochondrial length. The following sentences were added in the revised manuscript.

Methods section-page 25

"To examine mitochondrial length, mitochondrial morphology was imaged by Structured Illumination Microscopy (N-SIM, Nikon), using 100x oil immersion. The neurons in the out layer of the organoids were selected. For each image, about 50-100 mitochondria in a random selected (15μm x 15μm) area were analyzed. The freehand tool in Image J was used to manually trace the maximum length or the diameter of each mitochondrion."

Comment 3: A graphic abstract or a diagram to summarize the molecular mechanism would be helpful to understand the content of this study.

Response: In the revised manuscript, we provided a synopsis showing the detailed mechanism of mitochondrial dysfunction induced by mitochondrial HSF1.

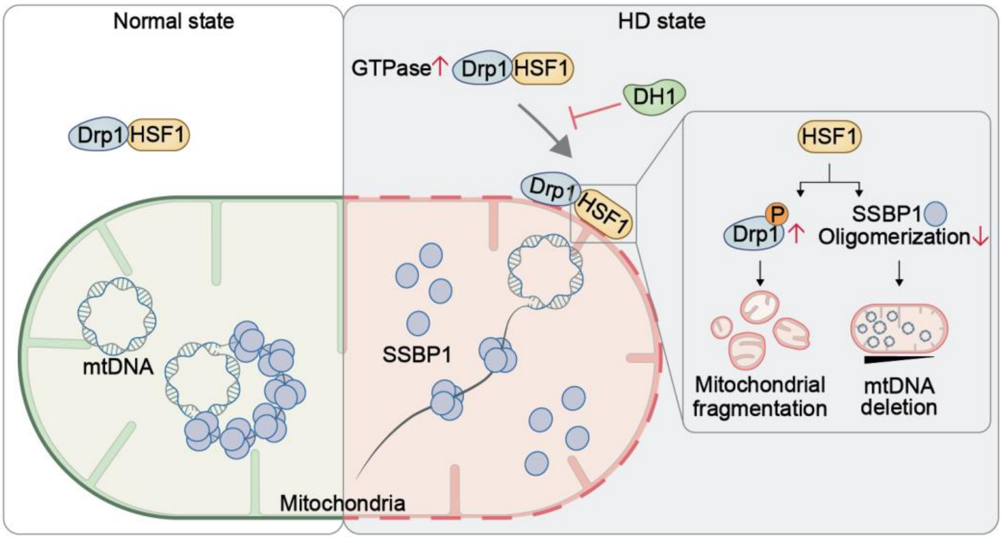

Mislocalization of proteins contributes to mitochondrial dysfunction and subsequent neurodegeneration. In this study, we show that inhibition of mtHSF1 corrects mitochondrial function and slows neurodegeneration, suggesting that mtHSF1 might be a therapeutic target to treat HD.

- HSF1 mislocalizes to mitochondria in various HD models.
- mtHSF1 induces mitochondrial dysfunction by disrupting dynamic balance and mtDNA replication.
- mtHSF1 causes neurodegeneration and HD-like behavior

- HD–associated neuropathology and behavioral deficits are improved by peptide inhibitor DH1.

Comment 4: Figure 1. The authors should show the HTT proteins in the patients' fibroblasts.

Response: Based on the reviewer's suggestion, we provided new data about the HTT proteins in the patients' fibroblasts by anti-1C2 and anti-HTT antibodies **(Please see Fig EV1B in the revised manuscript).**

Comment 5: It is desirable that the authors confirm the increased HSF1 translocation to mitochondria by immunofluorescence.

Response: Based on the reviewer's comment, we now provide new immunostaining images of HSF1/Tom20 in WT or HD striatal organoids **(Please see Fig 1H in the revised manuscript).** We found that in HD striatal organoids, there were more HSF1 accumulated in mitochondria than that in WT striatal organoids, further demonstrating HSF1 translocation to mitochondria in HD. To make the manuscript become more logical, we moved Fig 2H and I to Fig 1. The figure and the following sentences were added in the revised manuscript.

Result section-page 6

"HD striatal organoids exhibited increased colocalization of HSF1 and mitochondria compared with WT striatal organoids (Fig 1H)."

Figure legend section -page 33

"H. Representative images and scatterplot confirming the increased HSF1 (green) translocation to mitochondria (Tom20, red). The data were obtained from 3 independent biological experiments. Con: n=15 organoids; HD: n=14 organoids. Two-tailed unpaired t-test; Mean ± s.e.m. Images were taken using Structured Illumination Microscopy (SIM). Scale bar represents 5 μm."

Comment 6: It is desirable to provide higher-quality images in Fig.1F.

Response: Based on the reviewer's suggestion, we performed immunogold electron microscopy to examine the HSF1 in mitochondria in HD striatal organoids **(Please see Fig 1I in the revised manuscript).**

Comment 7: The authors should provide quantitative real-time PCR data in Fig.1G.

Response: Based on the reviewer's suggestion, we performed real-time PCR to determine the gene expression of huntingtin. We found that the mRNA level of huntingtin was significantly downregulated in striatal cells by shRNA targeting huntingtin **(Please see Fig EV2J in the revised manuscript).**

Methods section-page 20

Real-time PCR

Total RNA was isolated using RNA simple Total RNA Kit (DP419, TIANGEN). cDNA was synthesized by reverse transcription using 0.5µg of total RNA with HiScript III RT SuperMix for qPCR (+gDNA wiper) (R323-01, Vazyme), and was diluted 10-fold for real-time analysis. Quantitative real-time PCR reaction was followed by AceQ qPCR SYBR Green Master Mix (Q111-02, Vazyme) on an Applied Biosystems™ QuantStudio™ 3 Real-Time PCR System (Thermo Fisher Scientific). The results were normalized by GAPDH. The following primers were used for real-time PCR: mouse HTT-sense:5'-AGGGAGGAAGGAGCCAAAATC-3'; mouse HTT-antisense:5'-AGGGAGGAAGGAGCCAAAATC-3'; mouse GAPDH-sense:5'-TGGCCTTCCGTGTTCCTAC-3'; mouse GAPDH-antisense:5'-GAGTTGCTGTTGAAGTCGCA-3'.

Expanded View Figure Legend section -page 2

"J. The htt gene expression was measured by real-time PCR. (n=3 biological replicates). The data are the means ± SEMs; unpaired Student's t-test was used. *P < 0.05, ****P < 0.0001."

Comment 8: The authors should indicate the number of mitochondria used for statistical quantification in Fig.2G.

Response: We added the number of mitochondria analyzed in different group in our revised manuscript

Figure legend section -page 35

"G. Mitochondrial morphology was analyzed by electron microscopy (EM) in mice injected with AAV-Con or AAV-mtHSF1. The scale bar presents 4 µm, and the enlarged image scale bar is 400 nm. (n=3 mice/group). AAV-Con group, n=268 mitochondria were measured; AAV-Flag-mtHSF1 group, n=179 mitochondria were measured."

Comment 9: The author should clarify where the Drp1 phosphorylation occurs? Is it in the cytoplasm or in mitochondria? Is the level of phosphorylated Drp1 increased by expressing the CAG repeats?

Response: We sincerely thank the reviewer for such insightful comments. That's important points. We provided new data showing that mtHSF1 induced p-Drp1 S616 occurs in mitochondria but not cytosol **(Please see Fig EV3D and E in the revised manuscript)**. In addition, we performed WB analysis to determine the p-Drp1 S616 levels in Q23 or Q73 expressing cells. We found that the protein levels of p-Drp1 S616 were increased in Q73 expressing cells compared with controls **(please see Fig EV3C in the revised manuscript).** The figures and the following sentences were added in the revised manuscript.

Result section-page 7

"Overexpression of Httex1-Q73 promotes the phosphorylation of Drp1 at the S616 residue (p-Drp1 S616), which is a key step for Drp1 activation and mitochondrial fission

(Fig EV3C). Strikingly, the p-Drp1 S616 levels were elevated in mitochondria in Flag-mtHSF1-expressing cells (Figs 2C, EV3D and E)."

Expanded View Figure Legend section -page 3-4

"C. Myc-Q23 or Myc-Q73 were transfected into HdhQ7 cells. the p-Drp1 S616 levels was examined by WB analysis. (n=3 biological replicates). The data are the means ± SEMs; unpaired Student's t-test was used. ***P < 0.001."
"D-E. The protein levels of p-Drp1(S616) were determined in mitochondria-enriched fractions or cytosolic fractions by WB analysis. (n=3 biological replicates)."

Comment 10: The DAPI staining is missing in Fig.2E.

Response: We apologize for missing DAPI staining in Fig.2E. In the revised manuscript, we added the DAPI staining **(please see Fig 2E in the revised manuscript).**

Comment 11: The authors should show the mtDNA content in mtHSF1 expressed striatal organoids.

Response: Thanks for the helpful suggestions. In this revision, we measured the mtDNA content in mtHSF1 expressed striatal organoids by immunostaining assay. We found that, consistent with our findings in cell cultures and mouse brains, mtHSF1 expressed striatal organoids exhibited a decreased copy number of mtDNA **(please see Fig 3J in the revised manuscript).** The figures and the following sentences were added in the revised manuscript.

Result section-page 8

"Similarly, decreased mtDNA copy numbers were observed in mtHSF1-expressing mice and human striatal organoids (Fig 3F, G, I and J)."

Figure legend section -page 36

"J. Representative images and scatterplot showing the number of mtDNA (purple) colocalized with mitochondria (Tom20, green) in striatal organoids injected with AAV-mtHSF1. The data were obtained from 3 independent biological experiments. Con: n=33 cells from 10 organoids; Flag-mtHSF1: n=34 cells from 10 organoids. Two-tailed unpaired t-test was used. Images were captured by Structured Illumination Microscopy (SIM). Scale bar represents 5 μm."

Comment 12: The authors should confirm that the cells with fragmented nuclei are neurons in Fig.3I and 7F. Immunostaining for Tuj1 or NeuN would be helpful.

Response: We thank the valuable suggestions. It was reported that nuclear fragmentation is the typical biochemical index of cell apoptosis (Zhang et al., Mol Cancer. 2010 Aug 6; 9: 211). In this revision, we updated Fig. 3I and 7F by neural maker Tuj1 co-immunostaining with DAPI **(please see Fig 3K and 7F in the revised manuscript).**. The figures and the following sentences were modified in the revised manuscript.

Figure legend section-page 37

"K. The typical nuclear fragmentation morphology of AAV-Con (GFP) or AAV-mtHSF1-injected striatal organoids was measured. The arrows (yellow) mark fragmented nuclei. Two-tailed unpaired t-test was used. The data were obtained from 3 independent biological experiments. Con: n=14 organoids; Flag-mtHSF1: n=15 organoids. The scale bar represents 20 μm."

Comment 13: Please explain the principle used by the authors in designing the inhibition peptide of HSF1-Drp1.

Response: Two non-related proteins that interact in an inducible manner often have shared short sequences of homology that could represent binding sites of both inter- and intra-molecular interactions (1-4). A peptide corresponding to that sequence can serve as decoys for one of the proteins, preventing the binding of that protein to the target protein (1-3, 5). Although peptides represent only a part of the interaction surface, previous studies including ours showed that short peptides derived from the interaction sites between two proteins act as highly specific inhibitors and are effective agents in basic research and in animal models of human diseases, such as neurological disorders, cardiovascular diseases, cancer, and diabetes (5-14). My previous lab has used the rationally designed short peptide inhibitor P110 which is a peptide inhibitor that interferes with the mitochondrial fission protein Drp1 and its binding protein, Fis1 (14). We used P110 as a pharmacological tool to identify the role of these proteins in mitochondrial signal transduction in HD (15) and other disease models (6-8). Other peptides were generated to study the functional consequences of protein kinase C [PKC(16, 17)] by the Mochly-Rosen lab. These peptides have been used by many laboratories for *in vitro* and *in vivo* testing of a variety of species including humans. Thus, we have had considerable success in designing, synthesizing, and characterizing peptide inhibitors that are highly specific for the targeted protein-protein interaction.

1. Ron D, and Mochly-Rosen D. An autoregulatory region in protein kinase C: the pseudoanchoring site. *Proceedings of the National Academy of Sciences of the United States of America.* 1995;92(2):492-6.
2. Qvit N, and Mochly-Rosen D. Highly Specific Modulators of Protein Kinase C Localization: Applications to Heart Failure. *Drug discovery today Disease mechanisms.* 2010;7(2):e87-e93.
3. Souroujon MC, and Mochly-Rosen D. Peptide modulators of protein-protein interactions in intracellular signaling. *Nature biotechnology.* 1998;16(10):919-24.
4. Kheifets V, and Mochly-Rosen D. Insight into intra- and inter-molecular interactions of PKC: design of specific modulators of kinase function. *Pharmacol Res.* 2007;55(6):467-76.
5. Churchill EN, Qvit N, and Mochly-Rosen D. Rationally designed peptide regulators of protein kinase C. *Trends Endocrinol Metab.* 2009;20(1):25-33.
6. Guo X, Sesaki H, and Qi X. Drp1 stabilizes p53 on the mitochondria to trigger necrosis under oxidative stress conditions in vitro and in vivo. *Biochem J.* 2014;461(1):137-46.
7. Su YC, and Qi X. Inhibition of excessive mitochondrial fission reduced aberrant autophagy and neuronal damage caused by LRRK2 G2019S mutation. *Hum Mol Genet.* 2013;22(22):4545-61.
8. Disatnik MH, Ferreira JC, Campos JC, Gomes KS, Dourado PM, Qi X, and Mochly-Rosen D. Acute inhibition of excessive mitochondrial fission after myocardial infarction prevents long-term cardiac dysfunction. *J Am Heart Assoc.* 2013;2(5):e000461.

9. Inagaki K, Chen L, Ikeno F, Lee FH, Imahashi K, Bouley DM, Rezaee M, Yock PG, Murphy E, and Mochly-Rosen D. Inhibition of delta-protein kinase C protects against reperfusion injury of the ischemic heart in vivo. *Circulation.* 2003;108(19):2304-7.
10. Qi X, Inagaki K, Sobel RA, and Mochly-Rosen D. Sustained pharmacological inhibition of deltaPKC protects against hypertensive encephalopathy through prevention of blood-brain barrier breakdown in rats. *The Journal of clinical investigation.* 2008;118(1):173-82.
11. Palaniyandi SS, Sun L, Ferreira JC, and Mochly-Rosen D. Protein kinase C in heart failure: a therapeutic target? *Cardiovascular research.* 2009;82(2):229-39.
12. Kim J, Choi YL, Vallentin A, Hunrichs BS, Hellerstein MK, Peehl DM, and Mochly-Rosen D. Centrosomal PKCbetaII and pericentrin are critical for human prostate cancer growth and angiogenesis. *Cancer Res.* 2008;68(16):6831-9.
13. Budas GR, Churchill EN, and Mochly-Rosen D. Cardioprotective mechanisms of PKC isozyme-selective activators and inhibitors in the treatment of ischemia-reperfusion injury. *Pharmacol Res.* 2007;55(6):523-36.
14. Qi X, Qvit N, Su YC, and Mochly-Rosen D. A novel Drp1 inhibitor diminishes aberrant mitochondrial fission and neurotoxicity. *J Cell Sci.* 2013;126(Pt 3):789-802.
15. Guo X, Disatnik MH, Monbureau M, Shamloo M, Mochly-Rosen D, and Qi X. Inhibition of mitochondrial fragmentation diminishes Huntington's disease-associated neurodegeneration. *J Clin Invest.* 2013;123(12):5371-88.
16. Souroujon MC, and Mochly-Rosen D. Peptide modulators of protein-protein interactions in intracellular signaling. *Nat Biotechnol.* 1998;16(10):919-24.
17. Chen L, Wright LR, Chen CH, Oliver SF, Wender PA, and Mochly-Rosen D. Molecular transporters for peptides: delivery of a cardioprotective epsilonPKC agonist peptide into cells and intact ischemic heart using a transport system, R(7). *Chem Biol.* 2001;8(12):1123-9.

REFEREE #2:

The paper by Liu et al. explores a new role of HSF1 in the mitochondrial dysfunction and neurodegeneration in HD. The use of multiple in vitro and in vivo models, in combination with different and complementary techniques, makes this study a complete and outstanding work that uncover how mtHSF1 can contribute to mitochondrial dysfunction in HD.

Response: We appreciate the reviewer's positive comments on our current works. According to the concerns, we have revised the manuscript and the responses are listed below point by point.

Figure 1

Comment 1: Immunofluorescence of the endogenous HSF1, in combination with a mitochondria marker (Tom20 or others), should be performed, at least in wt and HD cellular models, to confirm the accumulation of HSF1 in mitochondria as shown by western blot analyses.

Response: Based on the reviewer's comment, we now provide new immunostaining images of HSF1/Tom20 in WT or HD striatal organoids **(Please see Fig 1H in the revised manuscript).** We found that in HD striatal organoids, there were more HSF1 accumulated in mitochondria than that in WT striatal organoids, further demonstrating

HSF1 translocation to mitochondria in HD. To make the manuscript become more logical, we moved Fig 2H and I to Fig 1. The figure and the following sentences were added in the revised manuscript.

Result section-page 6

"HD striatal organoids exhibited increased colocalization of HSF1 and mitochondria compared with WT striatal organoids (Fig 1H)."

Figure legend section -page 33

"H. Representative images and scatterplot confirming the increased HSF1 (green) translocation to mitochondria (Tom20, red). The data were obtained from 3 independent biological experiments. Con: n=15 organoids; HD: n=14 organoids. Two-tailed unpaired t-test; Mean ± s.e.m. Images were taken using Structured Illumination Microscopy (SIM). Scale bar represents 5 μm."

Comment 2: The authors checked HSF1 levels in total lysate from wt and HD fibroblasts and they found no differences. What about the other models? Although the focus of this study is the distribution of HSF1 in mitochondria, further data indicating altered translocation of this protein in HD models may be required.

Response: We sincerely thank the reviewer for such insightful comments. In the original manuscript, we tested the HSF1 total protein levels in striatal cells (Fig EV1G, left panel), fibroblasts (Fig EV1F) and iPS cells (Fig EV1G, second panel). Following the reviewer's suggestion, we determined the total protein level of HSF1 in mouse model and striatal organoids. Consistently, we observed that the total protein levels of HSF1 were not different between the HD models (YAC128 mice or striatal organoids) and their controls **(Please see Fig EV1G in the revised manuscript).** The figure and the following sentences were added in the revised manuscript.

Expanded View Figure Legend section -page 1

"G. Total HSF1 protein levels were tested in the indicated samples by immunoblotting. (n=3 biological replicates)."

Comment 3: In figure 1B, the authors used different proteins (HSP60 or VDAC) to normalize mtHSF1 in different samples. Why? Is only a technical issue?

Response: We thank the reviewer for this comment. We use HSP60 or VDAC as loading control in this experiment. we now provided a new data using VDAC as loading control. **(Please see Fig 1B in the revised manuscript).**

Comment 4: Supplementary figure 1D is not clear. In which cells the authors over-expressed muHTT?

Response: We thank the reviewer for this comment. Htt containing 23Q or 73Q repeats were co-expressed with Flag-HSF1 in HEK 293 cells **(Please see Expanded View Figure legend 1E).**

Comment 5: In the methods, the protocol to purify mitochondria from tissues is missing. Only the protocol from cells is described. Furthermore, did the author perform a quality check of the mitochondria purification?

Response: We thank the reviewer for pointing out the missing. Detailed information about the mitochondrial purification from tissues have been added in the revised manuscript. In addition, we repeated most of experiments in Fig. 1 to exclude the

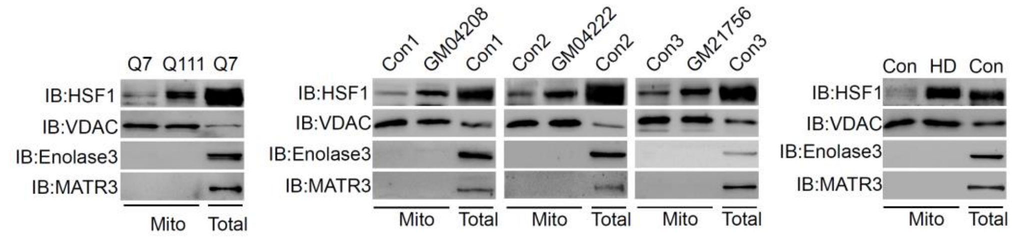

cytoplasmic and/or nuclear contamination in our mitochondrial fractions. Matrin3, a nuclear matrix protein containing both DNA and RNA binding domains, was used as a marker of nuclear protein. Enolase3, a cytosolic protein was used as a marker of cytosol. Based on these new data, we believe that there is no cytoplasmic and/or nuclear contamination in our mitochondrial fractions **(please see the figure below)**. The following sentences were added in the revised manuscript.

Methods section-page 20

"Mouse brains were minced and homogenized in the lysis buffer and then placed on ice for 30 min. Collected cells or tissue were transferred to tubes and then disrupted 20 times by repeated aspiration through a 25-gauge needle."

Figure 2

Comment 6: In figure 2F, I suppose that the WB analyses were performed in the striatum of mice. Please, confirm it.

Response: We thank the reviewer for the comment. AAV-Con and AAV-Flag-mtHSF1 were injected into the striatum. We harvested the total proteins from striata and measured the p-Drp1 S616 levels **(please see the methods section and the legend of Fig 2F).**

Comment 7: In the striatal organoids, what is the % of MSNs? What is the % of DARPP32+NeuN+ cells? Are there differences between control and HD organoids?

Response: We appreciate for the valuable comments. In the WT striatal organoids, the percentage of DARPP32 (a marker for MSN) positive cells in total cells was 33.66% ± 1.92% **(please see the figure below)**. We found all the DARPP32 positive cells are positive for NeuN, indicating the percentage of DARPP32+NeuN+ cells in total cells was also 33.66% ± 1.92%. Furthermore, we also quantify the percentage of DARPP32 positive cells in HD organoids (Fig 7D), which was significantly lower than control.

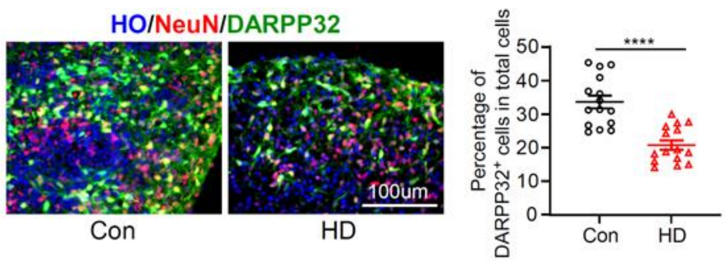

Representative images and quantitative results showing MSNs marker DAARPP32 (green) co-staining with neuronal marker NeuN (red). Data has been repeated from 3 biological experiments. n=15 organoids. Two-tailed unpaired t-test; Mean ± SEMs. Images were taken using a Nicon positive fluorescence microscope. Scale bar represents 100 μm.

Figure 3

Comment 8: Figure 3A is not clear to not experts. Additional infos about how DNA content is quantified by real time PCR for specific genes should be included in the legend and in the Method section.

Response: We appreciate for the valuable comments. In this revision, we added more information about the measurement of mtDNA content. The following sentences were added in the revised manuscript.

Methods section-page 26

Measurement of mtDNA content

Total DNA was purified using a TIANamp Genomic DNA Kit (DP304, Tiangen Biotech Co., Ltd.). The nuclear DNA (GAPDH or Tert) and mtDNA (mt-CO1, mt-CO2, mt-ND2, and D-loop) were analyzed in triplicate by qPCR under standard conditions using the AceQ qPCR SYBR Green Master Mix (Q121-02, Vazyme). The mtDNA abundance were calculated using the delta Ct (ΔCt) of average Ct of mtDNA and nDNA (ΔCt = CtmtDNA-CtnDNA) in the same well as $2^{-\Delta CT}$.

Figure legend section -page 36

"A. The mtDNA content was examined with primers of D-loop, mtCO1, mtCO2 and mtND2 by qPCR in control or Flag-mtHSF1-expressing striatal cells. (n=3 biological replicates)."

Comment 9: In figure 3I, which AAV was used? Did the authors perform a double viral infection with Flag-mtHSF1 and EGFP? The method about how the authors quantified nuclear fragmentation in striatal organoids is not described.

Response: We thank the reviewer for this comment. In this study, we used the AAV serotype 9 (rAAV9), which is widely used in the neuroscience research and the SMA gene therapy. We added more information about the quantification of nuclear fragmentation. The following sentences were added in the revised manuscript.

Methods section-page 19

“To examine nuclear fragmentation, cells in the out layer of the organoids, which are almost neurons, were selected. The nucleus with obvious dot fragments and higher fluorescence intensity was defined as a fragmented nucleus.”

Figure 5

Comment 10: The method to quantify neurite length is missing.

Response: We apologize for the missing information. In this revision, we provided relevant descriptions in methods section. The following sentences were added in the revised manuscript.

Methods section-page 25

“To quantify neurites length, we employed Neuroanatomy (from Fiji) software to trace DARPP32-positive neurites. Tracings were color coded: red for primary neurite (extension directly from the soma), blue for secondary neurite (branching from a primary), yellow for tertiary neurite (branching from a secondary).”

Comment 11: The method to quantify % Neu+ cells is missing.

Response: We apologize for the missing information. In this revision, we provided relevant descriptions in methods section. The following sentences were added in the revised manuscript.

Methods section-page 25

“To quantify the percentage of NeuN positive cells over total cells (% Neu+), fields in the out layer of the organoids were randomly captured. Around 10 organoids (11 organoids in con vs 13 organoids in Flag-mtHSF1) from three independent differentiation were randomly selected. GraphPad Prism version 9.0.0 was used for statistical analyses.”

Comment 12: Please verify the consistency in AAV control between Methods (AAV-EYFP) and the legend in figure 5C (GFP).

Response: We thank the reviewer for this comment. In this study, we used anti-GFP (Invitrogen, 14-6674-82; Chemicon, ab3080; Millipore, ab16901). All these antibodies can recognize YFP well.

General comments:

The first comment is related to the findings described in this work compared to HSF1 data in the literature. HSF1's role is related to heat shock response, which is impaired in HD mouse models. However, HSF1 level is not altered in a consistent way in all HD models. For example, recently, the basal or induced levels of HSF1 in the brain of zQ175 mice is similar to Wt mice (Gomez-Parades et al., Scientific Reports 2021). A comment about this issue should be included in the discussion.

Response: We appreciate for the valuable comments. The following sentences were added in the discussion section.

Discussion- page15

“The malfunction of heat shock response has been reported in various HD models. However, recent studies including ours show that the total protein levels of HSF1 are not changed between HD models and their controls, suggesting that mtHtt suppressed heat shock response is not caused by impairing the stability of HSF1(Gomez-Paredes et al, 2021; Labbadia et al, 2011). mtHtt disrupts the binding affinity of HSF1 and the promoters of downstream genes, which may contribute to the pathogenesis of HD (Labbadia et al, 2011).

The second comment is the relationship between HSF1 and cancer. HSF1 also promotes cancer development and progression. How the findings found in HD models are related (or not) with HSF1's role in cancers?

Response: Thanks for the valuable comments. In this study, we reported that recruitment of HSF1 to mitochondria caused mitochondrial fragmentation and mtDNA deletion by activating Drp1 phosphorylation and suppressing SSBP1 oligomers formation. Accumulated evidence reveals that HSF1 abundance are significantly increased in various cancers (1). It is possible that excessive HSF1 was aberrantly recruited to mitochondria through Drp1/HSF1 interactions, which in turn results in mitochondrial fragmentation and mtDNA deletion in cancer. Interestingly, decreases in mtDNA content have been observed in a range of cancers, including breast cancer, bladder urothelial carcinoma, hepatocellular carcinoma and non–small cell lung cancer (NSCLC) (2, 3). In addition, phosphorylation of Drp1 on Serine 616 and mitochondrial fragmentation are increased in human pancreatic cancer, nasopharyngeal carcinoma, hepatocellular carcinoma (4-6). These lines of evidence suggest that mtHSF1 may be involved in regulating mitochondrial function and cancer development. However, other mechanisms may exist, which remain to be further investigated.

1. Mendillo ML, Santagata S, Koeva M, Bell GW, Hu R, Tamimi RM, et al. HSF1 drives a transcriptional program distinct from heat shock to support highly malignant human cancers. Cell. 2012;150(3):549-62.

2. Reznik E, Miller ML, EħenbabaoДџlu Y, Riaz N, Sarungbam J, Tickoo SK, et al. Mitochondrial DNA copy number variation across human cancers. eLife. 2016;5.

3. Lai YH, Liu HY, Huang CY, Chau YP, Wu S. MitochondrialвЂ‘DNAвЂ‘associated TLR9 signalling is a potential serological biomarker for nonвЂ‘small cell lung cancer. Oncology reports. 2019;41(2):999-1006.

4. Kashatus JA, Nascimento A, Myers LJ, Sher A, Byrne FL, Hoehn KL, et al. Erk2 phosphorylation of Drp1 promotes mitochondrial fission and MAPK-driven tumor growth. Molecular cell. 2015;57(3):537-51.

5. Xie L, Shi F, Li Y, Li W, Yu X, Zhao L, et al. Drp1-dependent remodeling of mitochondrial morphology triggered by EBV-LMP1 increases cisplatin resistance. Signal transduction and targeted therapy. 2020;5(1):56.

6. Yi T, Luo H, Qin F, Jiang Q, He S, Wang T, et al. LncRNA LL22NC03-N14H11.1 promoted hepatocellular carcinoma progression through activating MAPK pathway to induce mitochondrial fission. Cell death & disease. 2020;11(10):832.

Uncropped blots should be shown in Supplementary material.

Response: We appreciate for the valuable comments. All uncropped gels are submitted as Appendix figures 2-11.

In the legends, several infos are missing, such as the type of graph, the number of biological or technical replicates.

Response: We appreciate for the valuable comments. The type of graph and number of biological replicates were added in the figure legend section. All detailed information was summarized at the bottom of each figure legend.

REFEREE #3:

In the current manuscript, authors studied the impact of heat shock transcription factor 1 (HSF1) in relation to mitochondria. They tested their concept using HD cell cultures, YAC128 mice, human striatal organoids derived from iPSc. Interestingly they found HSF1 in the mitochondria. They provided compelling evidence to support their conclusion. Methods are acceptable, overall, it is a well done study.

Response: We thank the reviewer for the constructive criticism and have made appropriate revisions according to the comments.

Comment 1: authors missed citing seminal articles on mitochondrial dysfunction, mitochondrial dynamics and biogenesis done by Shirendeb et al 2011 HMG and Shirendeb et al 2012 and most recently review article by Sawant et al 2021.

Response: We are so sorry for the missed articles and thank the reviewer for pointing out that. In the revised manuscript, we cite these references in the discussion.

Discussion section-page 15-16

Mitochondrial morphology is closely associated with neuronal cell survival in HD (Hwang et al, 2015; Shirendeb et al, 2011). Drp1 GTPase activity is triggered by mtHtt, which in turn accumulates in mitochondria, resulting in mitochondrial fragmentation (Guo et al, 2014; Shirendeb et al, 2012; Sawant et al, 2021).

Comment 2: It is unclear, if DH1 reduced full-length mutant huntingtin, please show the data, id experiments/blots are done.

Response: We thank the reviewer for this comment. Following the reviewer's suggestion, we determined the protein level of full-length mutant huntingtin in the mouse striatal cells treated with either DH1 or control peptide TAT. Treatment with DH1 had no effect on the protein levels of full-length mutant huntingtin in HdhQ111 cells **(Please see Fig EV4E in the revised manuscript).** The figure and the following sentences were added in the revised manuscript.

Result section-page 13

"Treatment with DH1 reduced HSF1 translocation to mitochondria in HD striatal cells, YAC128 mice, human striatal organoids, and two lines of patient fibroblasts (Fig 6G, H,

I and J), whereas the total protein levels of HSF1, Drp1 and HTT were not affected by the presence of DH1 (Fig EV4D and E).

Expanded View Figure Legend section -page 5

“D-E. HdhQ7 or HdhQ111 cells were treated with TAT or DH1 (1 μM, 3 days). Immunoblot analysis was performed to examine the total levels of Drp1, HSF1, HTT and SSBP1. (n=3 biological replicates).

Thank you for the submission of your revised manuscript to EMBO Molecular Medicine. We have now received the enclosed report from the two referees who were asked to re-assess it. As you will see, the referees are now supportive, and I am pleased to inform you that we will be able to accept your manuscript pending the following editorial-level amendments:

***** Reviewer's comments *****

Referee #1 (Comments on Novelty/Model System for Author):

The authors have addressed all the concerns and I recommend the publication of this manuscript.

Referee #1 (Remarks for Author):

The authors have addressed all the concerns and I recommend the publication of this manuscript.

Referee #2 (Comments on Novelty/Model System for Author):

The authors have improved the description of the methods as requested by all the referees. Technical quality and novelty are both high with impact in the HD field. The models used are adequate.

Referee #2 (Remarks for Author):

I have gone through the point-by-point response letter. The authors have answered to Referees' concerns and I appreciate the efforts made by the authors to improve the manuscript. New experiments have been performed and text and figures have been modified accordingly the Referees' suggestions. In my opinion, the resubmitted manuscript is suitable for publication.

Response letter

RE: Mitochondrial HSF1 triggers mitochondrial dysfunction and neurodegeneration in Huntington's disease (manuscript reference EMM-2022-15851-T)

Response to the comments from reviewers

REFEREE #1:

The authors have addressed all the concerns and I recommend the publication of this manuscript.

Response: We thank the reviewers for accepting our revised manuscript.

REFEREE #2:

I have gone through the point-by-point response letter. The authors have answered to Referees' concerns and I appreciate the efforts made by the authors to improve the manuscript. New experiments have been performed and text and figures have been modified accordingly the Referees' suggestions. In my opinion, the resubmitted manuscript is suitable for publication.

Response: We thank the reviewers for accepting our revised manuscript. We especially would like to thank the reviewers for their critical valuable comments which have helped us to improve our manuscript substantially.

Accepted **10th May 2022**

Please find enclosed the final reports on your manuscript. We are pleased to inform you that your manuscript is accepted for publication and is now being sent to our publisher to be included in the next available issue of EMBO Molecular Medicine.

EMBO Press Author Checklist

Corresponding Author Name: Guo Xing; Liu Yan	
Journal Submitted to: EMBO Molecular Medicine	
Manuscript Number: EMM-2022-15851-T	

USEFUL LINKS FOR COMPLETING THIS FORM
The EMBO Journal - Author Guidelines
EMBO Reports - Author Guidelines
Molecular Systems Biology - Author Guidelines
EMBO Molecular Medicine - Author Guidelines

Reporting Checklist for Life Science Articles (updated January 2022)

This checklist is adapted from Materials Design Analysis Reporting (MDAR) Checklist for Authors. MDAR establishes a minimum set of requirements in transparent reporting in the life sciences (see Statement of Task: 10.31222/osf.io/9sm4x). Please follow the journal's guidelines in preparing your manuscript.

Please note that a copy of this checklist will be published alongside your article.

Abridged guidelines for figures

1. Data

The data shown in figures should satisfy the following conditions:

- the data were obtained and processed according to the field's best practice and are presented to reflect the results of the experiments in an accurate and unbiased manner.
- ideally, figure panels should include only measurements that are directly comparable to each other and obtained with the same assay.
- plots include clearly labeled error bars for independent experiments and sample sizes. Unless justified, error bars should not be shown for technical replicates.
- if n<5, the individual data points from each experiment should be plotted. Any statistical test employed should be justified.
- Source Data should be included to report the data underlying figures according to the guidelines set out in the authorship guidelines on Data Presentation.

2. Captions

Each figure caption should contain the following information, for each panel where they are relevant:

- a specification of the experimental system investigated (eg cell line, species name).
- the assay(s) and method(s) used to carry out the reported observations and measurements.
- an explicit mention of the biological and chemical entity(ies) that are being measured.
- an explicit mention of the biological and chemical entity(ies) that are altered/varied/perturbed in a controlled manner.
- the exact sample size (n) for each experimental group/condition, given as a number, not a range;
- a description of the sample collection allowing the reader to understand whether the samples represent technical or biological replicates (including how many animals, litters, cultures, etc.).
- a statement of how many times the experiment shown was independently replicated in the laboratory.
- definitions of statistical methods and measures:
  - common tests, such as t-test (please specify whether paired vs. unpaired), simple χ2 tests, Wilcoxon and Mann-Whitney tests, can be unambiguously identified by name only, but more complex techniques should be described in the methods section;
  - are tests one-sided or two-sided?
  - are there adjustments for multiple comparisons?
  - exact statistical test results, e.g., P values = x but not P values < x;
  - definition of 'center values' as median or average;
  - definition of error bars as s.d. or s.e.m.

Please complete ALL of the questions below.
Select "Not Applicable" only when the requested information is not relevant for your study.

Materials

Newly Created Materials	Information included in the manuscript?	In which section is the information available? (Reagents and Tools Table, Materials and Methods, Figures, Data Availability Section)
New materials and reagents need to be available; do any restrictions apply?	Not Applicable	

Antibodies	Information included in the manuscript?	In which section is the information available? (Reagents and Tools Table, Materials and Methods, Figures, Data Availability Section)
For **antibodies** provide the following information:<br>- Commercial antibodies: RRID (if possible) or supplier name, catalogue number and or/clone number<br>- Non-commercial: RRID or citation	Yes	Appendix Table S2

DNA and RNA sequences	Information included in the manuscript?	In which section is the information available? (Reagents and Tools Table, Materials and Methods, Figures, Data Availability Section)
**Short novel DNA or RNA including primers, probes**: provide the sequences.	Yes	Material and Methods

Cell materials	Information included in the manuscript?	In which section is the information available? (Reagents and Tools Table, Materials and Methods, Figures, Data Availability Section)
**Cell lines:** Provide species information, strain. Provide accession number in repository **OR** supplier name, catalog number, clone number, and/**OR** RRID.	Yes	Material and Methods
**Primary cultures:** Provide species, strain, sex of origin, genetic modification status.	Yes	Material and Methods
Report if the cell lines were recently **authenticated** (e.g., by STR profiling) and tested for mycoplasma contamination.	Not Applicable	

Experimental animals	Information included in the manuscript?	In which section is the information available? (Reagents and Tools Table, Materials and Methods, Figures, Data Availability Section)
**Laboratory animals or Model organisms:** Provide species, strain, sex, age, genetic modification status. Provide accession number in repository **OR** supplier name, catalog number, clone number, **OR** RRID.	Yes	Material and Methods
**Animal observed in or captured from the field:** Provide species, sex, and age where possible.	Not Applicable	
Please detail **housing and husbandry conditions**.	Yes	Material and Methods

Plants and microbes	Information included in the manuscript?	In which section is the information available? (Reagents and Tools Table, Materials and Methods, Figures, Data Availability Section)
**Plants:** provide species and strain, ecotype and cultivar where relevant, unique accession number if available, and source (including location for collected wild specimens).	Not Applicable	
**Microbes:** provide species and strain, unique accession number if available, and source.	Not Applicable	

Human research participants	Information included in the manuscript?	In which section is the information available? (Reagents and Tools Table, Materials and Methods, Figures, Data Availability Section)
If collected and within the bounds of privacy constraints report on age, sex and gender or ethnicity for all study participants.	Not Applicable	

Core facilities	Information included in the manuscript?	In which section is the information available? (Reagents and Tools Table, Materials and Methods, Figures, Data Availability Section)
If your work benefited from core facilities, was their service mentioned in the acknowledgments section?	Not Applicable	

Design

Study protocol	Information included in the manuscript?	In which section is the information available? (Reagents and Tools Table, Materials and Methods, Figures, Data Availability Section)
If study protocol has been **pre-registered, provide DOI in the manuscript**. For clinical trials, provide the trial registration number **OR** cite DOI.	Not Applicable	
Report the **clinical trial registration number** (at ClinicalTrials.gov or equivalent), where applicable.	Not Applicable	

Laboratory protocol	Information included in the manuscript?	In which section is the information available? (Reagents and Tools Table, Materials and Methods, Figures, Data Availability Section)
Provide DOI OR other citation details if **external detailed step-by-step protocols** are available.	Not Applicable	

Experimental study design and statistics	Information included in the manuscript?	In which section is the information available? (Reagents and Tools Table, Materials and Methods, Figures, Data Availability Section)
Include a statement about **sample size** estimate even if no statistical methods were used.	Yes	Figures
Were any steps taken to minimize the effects of subjective bias when allocating animals/samples to treatment (e.g. **randomization procedure**)? If yes, have they been described?	Yes	Material and Methods
Include a statement about **blinding** even if no blinding was done.	Not Applicable	
Describe **inclusion/exclusion criteria** if samples or animals were excluded from the analysis. Were the criteria pre-established?<br><br>If sample or data points were omitted from analysis, report if this was due to attrition or intentional exclusion and provide justification.	Not Applicable	
For every figure, are **statistical tests** justified as appropriate? Do the data meet the assumptions of the tests (e.g., normal distribution)? Describe any methods used to assess it. Is there an estimate of variation within each group of data? Is the variance similar between the groups that are being statistically compared?	Yes	Figures

Sample definition and in-laboratory replication	Information included in the manuscript?	In which section is the information available? (Reagents and Tools Table, Materials and Methods, Figures, Data Availability Section)
In the figure legends: state number of times the experiment was **replicated** in laboratory.	Yes	Figures
In the figure legends: define whether data describe **technical or biological replicates**.	Yes	Figures

Ethics

Ethics	Information included in the manuscript?	In which section is the information available? (Reagents and Tools Table, Materials and Methods, Figures, Data Availability Section)
Studies involving **human participants**: State details of **authority granting ethics approval** (IRB or equivalent committee(s), provide reference number for approval.	Not Applicable	
Studies involving **human participants**: Include a statement confirming that **informed consent** was obtained from all subjects and that the experiments conformed to the principles set out in the WMA Declaration of Helsinki and the Department of Health and Human Services Belmont Report.	Not Applicable	
Studies involving **human participants:** For publication of **patient photos**, include a statement confirming that consent to publish was obtained.	Not Applicable	
Studies involving experimental **animals**: State details of **authority granting ethics approval** (IRB or equivalent committee(s), provide reference number for approval. Include a statement of compliance with ethical regulations.	Yes	Material and Methods
Studies involving **specimen and field samples:** State if relevant **permits** obtained, provide details of authority approving study; if none were required, explain why.	Not Applicable	

Dual Use Research of Concern (DURC)	Information included in the manuscript?	In which section is the information available? (Reagents and Tools Table, Materials and Methods, Figures, Data Availability Section)
Could your study fall under dual use research restrictions? Please check biosecurity documents and list of **select agents and toxins** (CDC): https://www.selectagents.gov/sat/list.htm	Not Applicable	
If you used a select agent, is the security level of the lab appropriate and reported in the manuscript?	Not Applicable	
If a study is subject to dual use research of concern regulations, is the name of the **authority granting approval and reference number** for the regulatory approval provided in the manuscript?	Not Applicable	

Reporting

The MDAR framework recommends adoption of discipline-specific guidelines, established and endorsed through community initiatives. Journals have their own policy about requiring specific guidelines and recommendations to complement MDAR.

Adherence to community standards	Information included in the manuscript?	In which section is the information available? (Reagents and Tools Table, Materials and Methods, Figures, Data Availability Section)
State if relevant guidelines or checklists (e.g., **ICMJE, MIBBI, ARRIVE, PRISMA**) have been followed or provided.	Not Applicable	
For **tumor marker prognostic studies,** we recommend that you follow the **REMARK** reporting guidelines (see link list at top right). See author guidelines, under 'Reporting Guidelines'. Please confirm you have followed these guidelines.	Not Applicable	
For **phase II and III randomized controlled trials**, please refer to the **CONSORT** flow diagram (see link list at top right) and submit the CONSORT checklist (see link list at top right) with your submission. See author guidelines, under 'Reporting Guidelines'. Please confirm you have submitted this list.	Not Applicable	

Data Availability

Data availability	Information included in the manuscript?	In which section is the information available? (Reagents and Tools Table, Materials and Methods, Figures, Data Availability Section)
Have **primary datasets** been deposited according to the journal's guidelines (see 'Data Deposition' section) and the respective accession numbers provided in the Data Availability Section?	Yes	Data Availability
Were **human clinical and genomic datasets** deposited in a public access-controlled repository in accordance to ethical obligations to the patients and to the applicable consent agreement?	Not Applicable	
Are **computational models** that are central and integral to a study available without restrictions in a machine-readable form? Were the relevant accession numbers or links provided?	Not Applicable	
If publicly available data were reused, provide the respective **data citations in the reference list**.	Not Applicable	
