# Supplementary material for: Mitochondrial HSF1 triggers mitochondrial dysfunction and neurodegeneration in Huntington's disease
Source: EMBO Mol Med. 2022 Jun 7;14(7):e15851. doi: 10.15252/emmm.202215851 (PMC9260212; doi:10.15252/emmm.202215851)

Expanded View Figure 1A

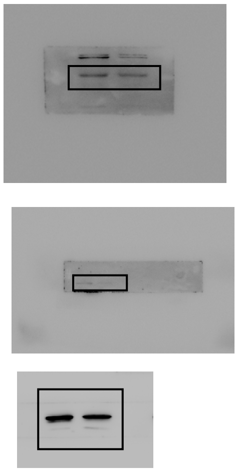

Expanded View Figure 1B

GM04208

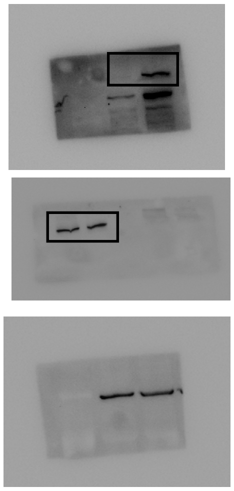

GM04222

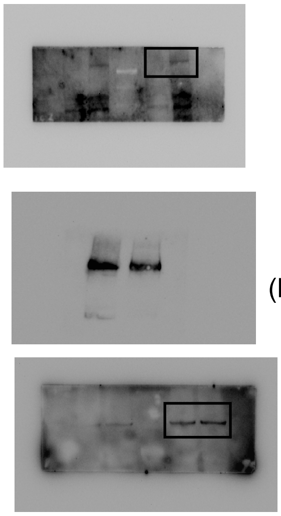

GM21756

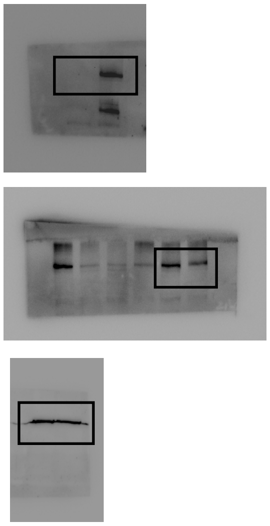

Expanded View Figure 1C

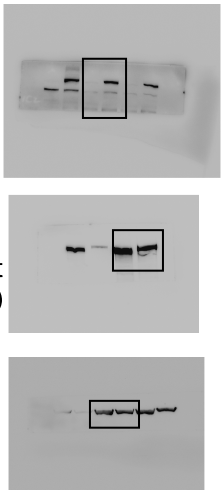

Expanded View Figure 1E

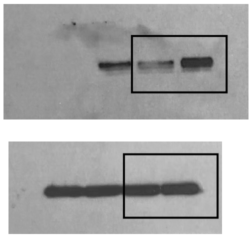

Expanded View Figure 1F

GM04208/GM04222

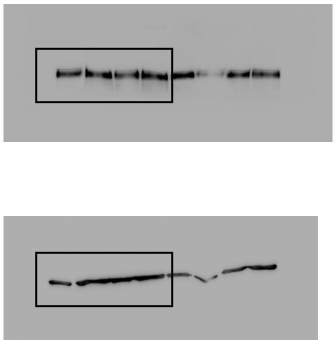

GM21756

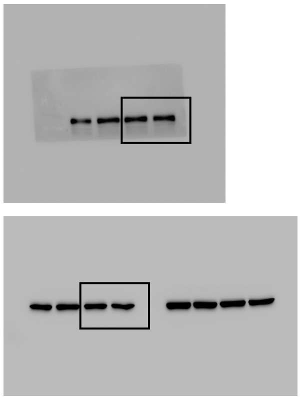

Expanded View Figure 1G

Striatal cells

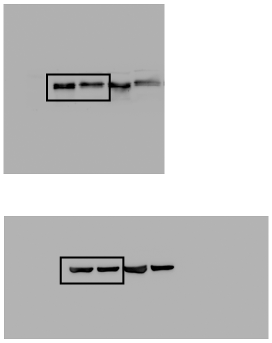

iPS cells

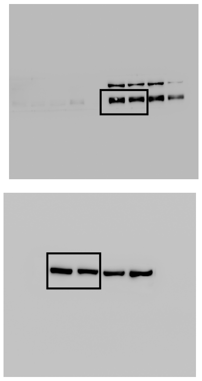

YAC128 mice

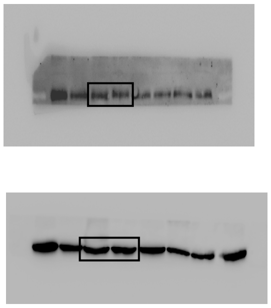

Striatal organoids

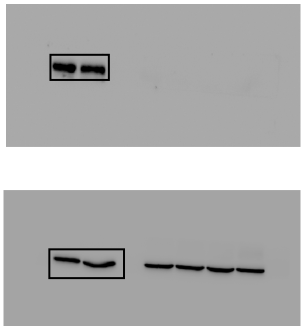

Supplement: Supplementary file 3 — Source Data for Expanded View and Appendix [file EMMM-14-e15851-s002.zip › Source Data for EV Figures.zip!/Source Data For Figure EV1.pdf]
