# Supplementary material for: Mitochondrial HSF1 triggers mitochondrial dysfunction and neurodegeneration in Huntington's disease
Source: EMBO Mol Med. 2022 Jun 7;14(7):e15851. doi: 10.15252/emmm.202215851 (PMC9260212; doi:10.15252/emmm.202215851)

Expanded View Figure 3A

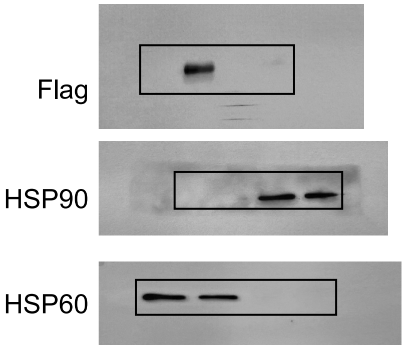

Expanded View Figure 3B

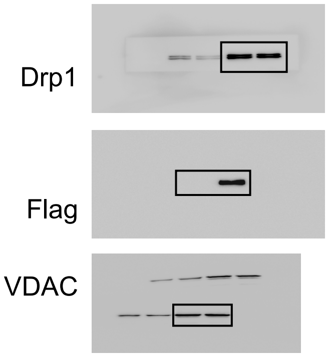

Expanded View Figure 3C

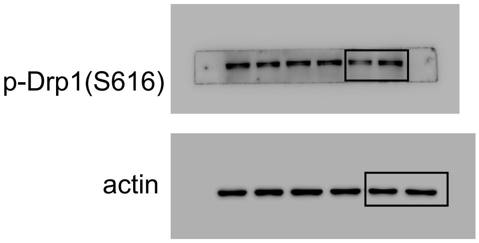

Expanded View Figure 3D

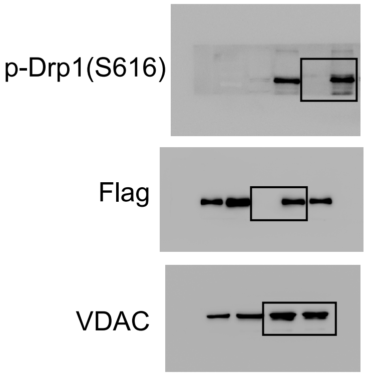

Expanded View Figure 3E

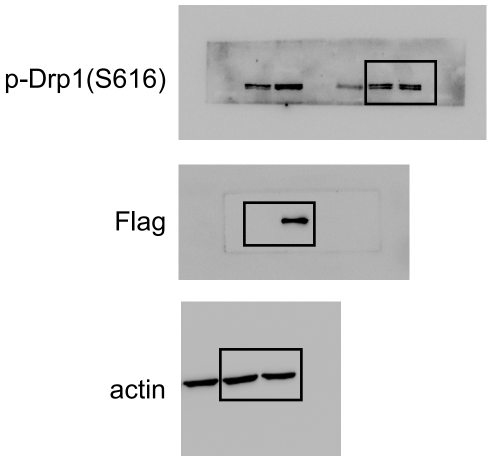

Expanded View Figure 3F

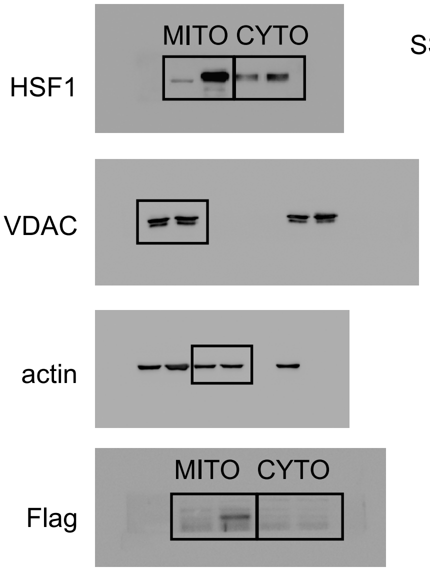

Expanded View Figure 3G

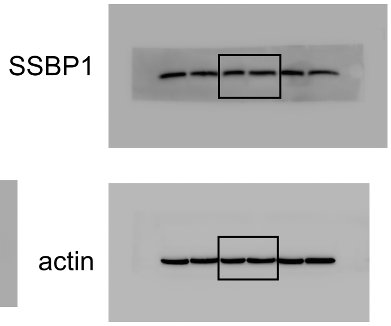

Expanded View Figure 3H

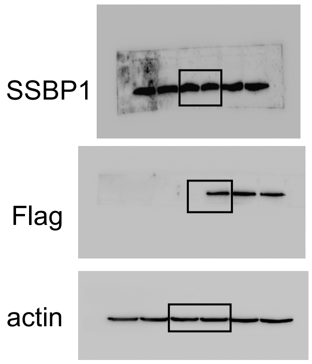

Expanded View Figure 3I

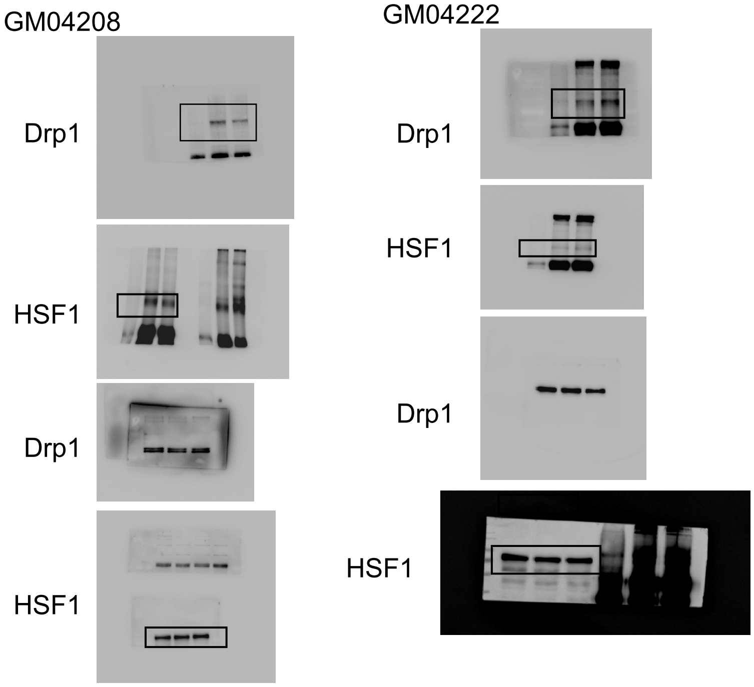

Expanded View Figure 3J

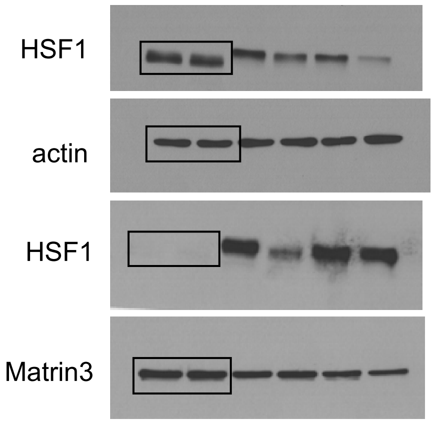

Expanded View Figure 3K

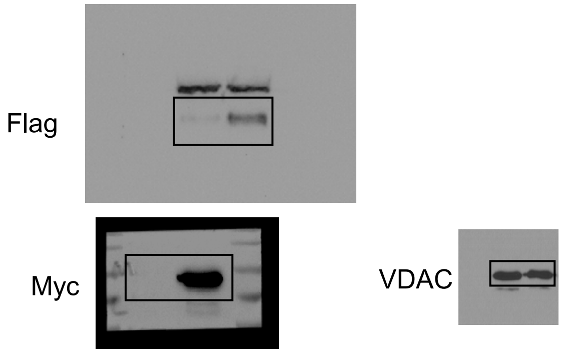

Supplement: Supplementary file 3 — Source Data for Expanded View and Appendix [file EMMM-14-e15851-s002.zip › Source Data for EV Figures.zip!/Source Data For Figure EV3.pdf]
