# Supplementary material for: Mitochondrial HSF1 triggers mitochondrial dysfunction and neurodegeneration in Huntington's disease
Source: EMBO Mol Med. 2022 Jun 7;14(7):e15851. doi: 10.15252/emmm.202215851 (PMC9260212; doi:10.15252/emmm.202215851)

Expanded View Figure 2I

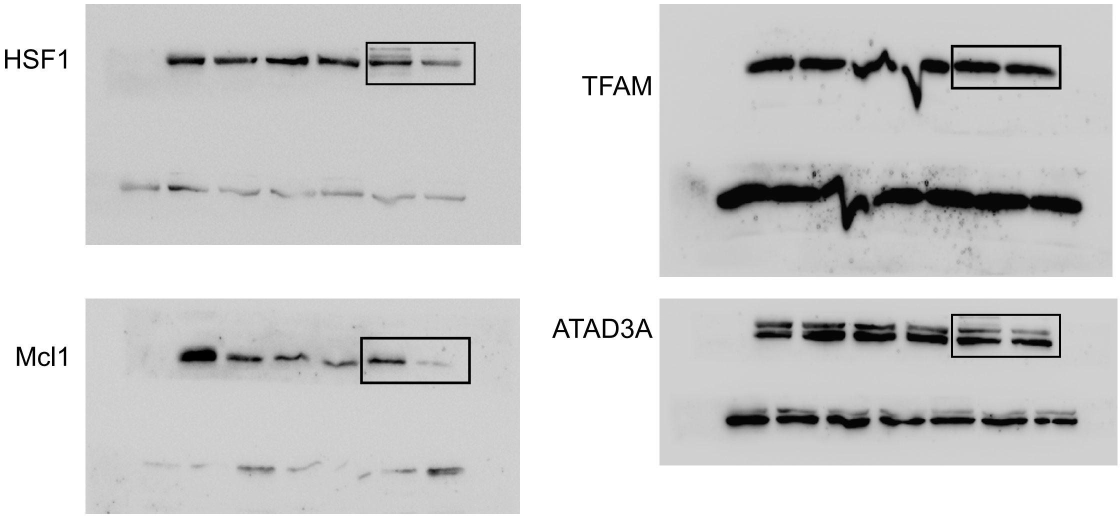

Supplement: Supplementary file 3 — Source Data for Expanded View and Appendix [file EMMM-14-e15851-s002.zip › Source Data for EV Figures.zip!/Source Data For Figure EV2.pdf]
